# Sequence-based genome-wide association study of individual milk mid-infrared wavenumbers in mixed-breed dairy cattle

**DOI:** 10.1186/s12711-021-00648-9

**Published:** 2021-07-20

**Authors:** Kathryn M. Tiplady, Thomas J. Lopdell, Edwardo Reynolds, Richard G. Sherlock, Michael Keehan, Thomas JJ. Johnson, Jennie E. Pryce, Stephen R. Davis, Richard J. Spelman, Bevin L. Harris, Dorian J. Garrick, Mathew D. Littlejohn

**Affiliations:** 1 Research and Development, Livestock Improvement Corporation, Private Bag 3016, Hamilton, 3240 New Zealand; 2 School of Agriculture, Massey University, Ruakura, Hamilton, 3240 New Zealand; 3 School of Applied Systems Biology, La Trobe University, Bundoora, VIC 3083 Australia; 4 Agriculture Victoria, AgriBio, Centre for AgriBioscience, Bundoora, VIC 3083 Australia

## Abstract

**Background:**

Fourier-transform mid-infrared (FT-MIR) spectroscopy provides a high-throughput and inexpensive method for predicting milk composition and other novel traits from milk samples. While there have been many genome-wide association studies (GWAS) conducted on FT-MIR predicted traits, there have been few GWAS for individual FT-MIR wavenumbers. Using imputed whole-genome sequence for 38,085 mixed-breed New Zealand dairy cattle, we conducted GWAS on 895 individual FT-MIR wavenumber phenotypes, and assessed the value of these direct phenotypes for identifying candidate causal genes and variants, and improving our understanding of the physico-chemical properties of milk.

**Results:**

Separate GWAS conducted for each of 895 individual FT-MIR wavenumber phenotypes, identified 450 1-Mbp genomic regions with significant FT-MIR wavenumber QTL, compared to 246 1-Mbp genomic regions with QTL identified for FT-MIR predicted milk composition traits. Use of mammary RNA-seq data and gene annotation information identified 38 co-localized and co-segregating expression QTL (eQTL), and 31 protein-sequence mutations for FT-MIR wavenumber phenotypes, the latter including a null mutation in the *ABO* gene that has a potential role in changing milk oligosaccharide profiles. For the candidate causative genes implicated in these analyses, we examined the strength of association between relevant loci and each wavenumber across the mid-infrared spectrum. This revealed shared association patterns for groups of genomically-distant loci, highlighting clusters of loci linked through their biological roles in lactation and their presumed impacts on the chemical composition of milk.

**Conclusions:**

This study demonstrates the utility of FT-MIR wavenumber phenotypes for improving our understanding of milk composition, presenting a larger number of QTL and putative causative genes and variants than found from FT-MIR predicted composition traits. Examining patterns of significance across the mid-infrared spectrum for loci of interest further highlighted commonalities of association, which likely reflects the physico-chemical properties of milk constituents.

**Supplementary Information:**

The online version contains supplementary material available at 10.1186/s12711-021-00648-9.

## Background

Fourier-transform mid-infrared (FT-MIR) spectroscopy is a high-throughput and inexpensive method for predicting milk composition. The FT-MIR methodology determines the presence of specific chemical bonds in milk by measuring the absorbance of infrared light as the light interacts with molecules in the sample. Data from FT-MIR spectroscopy comprises a spectrum of absorbance values across the mid-infrared range that are readily available through routine milk testing. This technology is widely used to estimate the concentrations of major milk components such as fat and protein for incorporation into milk payment and animal evaluation systems. Over the last decade, there has been increased interest in using FT-MIR data to predict other milk composition and novel traits. Applications of FT-MIR spectroscopy as a phenotyping tool have been widely studied and reviewed [1–4]. Recent research includes studies of milk composition traits that are relevant to manufacturing traits [5–7], individual fatty acids and milk proteins [8, 9], and indirect traits that are related to energy status [10, 11], pregnancy and fertility [12–14], methane emissions [15–17] and bovine tuberculosis [18].

Successful utilisation of FT-MIR data as a phenotyping tool depends on the strength of the phenotypic correlation between the predicted trait, and the trait as measured by a benchmarked standard; and successful incorporation of FT-MIR predicted traits into breeding programmes further depends on the heritability of the FT-MIR predicted trait, and the genetic correlation between the FT-MIR prediction and the benchmarked trait [19]. Studies have reported moderate to high heritability estimates for a range of FT-MIR predicted traits, including fatty acids [20–22], milk proteins [9, 23], cheese-making and milk-coagulation properties [24–26], and lactoferrin concentrations [27, 28]. Studies of individual FT-MIR spectra wavenumbers show that across most of the mid-infrared region, absorbances of individual FT-MIR spectra wavenumbers are moderately to highly heritable [29–32]. This suggests that there is potential for achieving genetic gain through the direct use of FT-MIR spectra for selection, rather than selection on FT-MIR predicted milk composition traits, which are themselves a function of the absorbance spectra at various wavenumbers.

Although there have been many genome-wide association studies (GWAS) for FT-MIR predicted milk composition traits such as fat, protein, and lactose concentrations [33–37], and individual fatty acid and protein fractions [38–40], there are comparatively few studies reporting GWAS results for individual FT-MIR wavenumber phenotypes [41–43]. Two such GWAS were conducted on medium density SNP-chip (~ 50 k markers) genotypes for a subset of wavenumbers, which were identified either by clustering analysis [41], or by using phenotypic correlation structures and heritability estimates within each breed [43]. A third study explored relationships between FT-MIR wavenumber phenotypes and a subset of SNPs that had previously been implicated in a GWAS of milk composition and fatty acid traits [42]. Across these studies, a number of FT-MIR wavenumber QTL were identified. Most of the detected genomic regions had been previously reported in studies of major milk composition traits, but new regions with potential links to milk contents such as phosphorus, orotic acid or citric acid were identified [41]. Thus, these findings have demonstrated that it is possible to identify genomic regions that are specifically related to individual FT-MIR wavenumber phenotypes.

Previous studies have examined the effects of variants in individual genes and their encoded proteins on FT-MIR wavenumber phenotypes [32, 42]. Wang et al*.* [32] observed that the *DGAT1* K232A polymorphism had highly significant effects on wavenumbers associated with carboxylic and ester C=O bond stretching, triglyceride ester linkage C-O stretching and alkyl C-H stretching. In that same study, a polymorphism in the *CSN3* gene had effects on wavenumbers that coincided with amide II, amide III and phosphate bands, and a polymorphism in the *PAEP* gene had effects on wavenumbers in a mid-infrared band that was attributed to C-N stretching [32]. Similar effects were also observed by Benedet et al*.* [42], with an additional absorption band associated with unsaturated fatty acids that was reported for a polymorphism near *CSN3*. Across those studies, association patterns varied widely for loci in different genes, with *DGAT1* having highly significant effects across many wavenumbers, while *PAEP* had significant effects across fewer wavenumbers that were concentrated within a small number of spectral bands. Assessing association patterns across the mid-infrared spectrum for a wider range of loci could improve our understanding of the impact that different genes have on the molecular structure of milk. Moreover, comparing these association patterns could provide insights into commonalities in the way genes influence milk composition and how these impacts are detected.

The purpose of the current study was to investigate the underlying genetics of milk composition, by conducting GWAS on 895 individual FT-MIR wavenumber phenotypes, and comparing these results to GWAS conducted on three FT-MIR predicted major milk composition traits. We report the use of a much larger sample (*N* = 38,085) than previous such studies and at a higher genomic resolution, with imputed whole-genome sequence consisting of 17,873,880 variants. We further report molecular dissection of these signals through the use of variant annotation information and a large mammary RNA-seq resource, identifying candidate causative genes and variants for a substantial number of loci. Finally, we evaluated patterns of significance across the mid-infrared range for different loci, highlighting clusters of QTL that are broadly defined by the biochemical properties of the molecules that they encode.

## Methods

### Study population, animals and milk samples

In total, 100,571 FT-MIR spectra records from individual milk test samples for 38,085 multi-breed and crossbred cows across 1645 herds were included for analysis. This dataset was a subset of a wider set of 2,044,094 FT-MIR spectra records analysed on six Bentley FTS (Chaska, MN, USA) instruments as part of routine milk testing conducted by Livestock Improvement Corporation (LIC), over the period from September 2017 to May 2018 [44]. Records were included in the present study if they passed outlier removal based on the Mahalanobis distance between each spectrum and the average within-instrument spectra for each analyser, and had imputed sequence available for the cow from which the milk sample was taken. The pedigree-based breed composition of cows comprised 11,235 cows with ≥ 14/16 Holstein (HOL) or Friesian (FR) genetics; 5374 cows with ≥ 14/16 Jersey (JE) genetics; 19,915 crossbred cows with HOL-FR (≥ 3/16) and JE (≥ 3/16) genetics only; 17 cows with ≥ 14/16 Ayrshire (AY) genetics; and 1544 cows from other breeds or crosses. Individual FT-MIR wavenumbers were subjected to piecewise direct standardization [45], with standardization coefficients evaluated from 16 weeks of reference sample calibration data collected across six Bentley instruments as in Tiplady et al*.* [44].

### Pre-adjustment of individual FT-MIR wavenumber and predicted milk composition phenotypes

Prior to conducting GWAS, adjusted cow phenotypes were generated for 895 individual FT-MIR wavenumbers and three FT-MIR predicted milk composition traits. Adjusted phenotypes were generated from one or more test-day samples on the same cow by fitting repeated measures models in ASReml-R [46], comprising:

1 $$y_{{ijkl}} = \mu + parity_{j} + dim_{k} + HD_{l} + \sum \alpha _{m} brd_{{im}} + \sum \delta _{n} het_{{in}} + anml_{i} + e_{{ijkl}} ,$$

where $y_{{ijkl}}$ is a test-day phenotype (e.g. absorbance for one wavenumber) for the $i$ th individual in parity class $j$ within the days in milk class $k$ and the herd-by-test date group $l$; $\mu$ is the overall mean; $parity_{j}$ is the fixed effect for parity $j$ (5 classes: 1, 2, 3, 4, ≥ 5); $dim_{k}$ is the fixed effect for the days in milk class $k$ (9 intervals of 30 days each from the start of lactation); $HD_{l}$ is the fixed effect for the herd by test day class $l$; $\alpha _{m}$ are breed linear regression coefficients for Holstein (HOL), Friesian (FR) and Jersey (JE) proportions and $brd_{{im}}$ are the corresponding breed proportions for individual $i$; $\delta _{n}$ are heterosis linear regression coefficients between breeds (FRxJE, FRxHOL, JExHOL, FRxAY, JExAY, AYxHOL) and $het_{{in}}$ are the corresponding heterosis proportions for individual $i$, according to sire and dam breed proportions; $anml_{i}$ is the random animal effect with $anml_{i}$ ~ $N\left( {0,{\mathbf{I}}\sigma _{{anml}}^{2} } \right)$; and $e_{{ijkl}}$ is the random error effect with $e_{{ijkl}} \sim N\left( {0,{\mathbf{I}}\sigma _{e}^{2} } \right)$, where ${\mathbf{I}}$ is an identity matrix and $\sigma _{{anml}}^{2}$ and $\sigma _{e}^{2}$ are the variances of the independent and identically distributed animal and error variances, respectively. Adjusted phenotypes were evaluated for individual $i$ as $y$ minus all the relevant fixed effects averaged over all observations for a cow, or equivalently, the sum of the prediction of $anml_{i}$ and the average of the predicted error terms for all test-day records for the animal, i.e. $\hat{y}_{{i\left( {adj} \right)}} = anml_{i} + \bar{e}_{{ij.}}$.

### Genotypes and imputation

Animals were genotyped on Illumina BovineHD (HD; *N* = 138; ~ 777 k SNP), Illumina BovineSNP50k (50 k; *N* = 4087; ~ 53 k SNP), and/or custom GeneSeek Genomic Profiler LDv3 BeadChip (GGP; *N* = 33,976; ~ 26 k SNP) panels, with the resultant genotypes imputed to sequence density as part of a wider set of 153,357 animals, as described by Jivanji et al*.* [47]. More detailed descriptions of SNP-chip data handling and imputation criteria are given below, and as a summary, this process consisted of step-wise imputation of animals to whole-genome sequence genotypes via references of GGP, 50 k and HD genotypes. Whole-genome sequences for 565 animals had been mapped and called from the UMD3.1 *Bos taurus* reference genome using BWA-MEM (v0.78-r455) [48], and GATK (v3.2) [49] respectively, as previously described [35, 36, 47]. The pedigree-based breed composition of sequenced animals comprised 138 Holstein-Friesians, 99 Jerseys, 316 Holstein–Friesian $\times$ Jersey crossbreeds and 12 from other breeds or crosses. Only variants located on *Bos taurus* autosomes were considered, and phasing with genotype probabilities was undertaken using Beagle 4.0 [50]. Variants were filtered to remove those for which the allelic ${R}^{2}$, defined as the estimated squared correlation between the most likely allele dosage and the true allele dosage [51] for missing genotypes was less than 0.95. This resulted in a sequence reference comprising 19,659,361 segregating variants spanning all 29 bovine autosomes.

#### SNP-chip imputation references

The reference sets for SNP chip panels used at each imputation step were generated based on a uniform set of criteria. Genotypes were eligible for inclusion in a reference if the sample call rate was ≥ 0.95, and the proportion of Mendelian inconsistences observed between parent–offspring pairs of genotypes was lower than 0.005. The 50 k reference included eligible Illumina BovineSNP50 BeadChip genotypes for all males, and females that were a dam of a genotyped sire or had at least five recorded progeny (46,621 SNPs; 10,786 animals). The GGP reference included eligible GGP LD BeadChip genotypes for all males, and females that had recorded progeny (20,846 SNPs; 11,872 animals). Additional 50 k reference SNPs that were not on the GGP panel were also included as a background scaffold, resulting in a reference with 57,493 SNPs across 11,872 animals. The HD reference included all available Illumina BovineHD BeadChip genotypes, predominantly from widely-used sires and/or sequenced animals (*N* = 3389), with 675,321 SNPs remaining after eligibility filters were applied.

For all references, SNPs that were monomorphic or had a batch call rate lower than 0.9 were excluded. Quality checks were made to ensure that allele frequencies in the reference population reflected those in the wider population. That is, for SNPs with a count of more than 1000 minor alleles in the overall population, the relationship between the minor allele frequency (MAF) in the reference population (MAF_ref_) and the MAF in the overall population (MAF_overall_) satisfied the criteria: |MAF_ref_-MAF_overall_|/MAF_ref_ < 0.4. This resulted in the removal of 12 SNPs from the Illumina BovineSNP50 BeadChip, and three SNPs from the GGP LDv3 BeadChip. In addition, for all references, SNPs that were in common with sequence variants with more than 30 × depth coverage were removed if the concordance between genotype and sequencing calls was ≤ 0.7. Likewise, for GGP and 50 k references, any SNPs that were shared with the BovineHD panel were removed if the concordance between genotype calls from each panel was ≤ 0.7; and for the HD reference, any SNPs that were shared with the BovineSNP50 panel were removed if the genotypic concordance between panels was ≤ 0.7.

#### Imputation

All imputation steps were carried out ignoring pedigree information using Beagle 4.0 [50]. Imputation of animals to GGP, 50 k and HD references was carried out using default parameters, except for window sizes which were adjusted to ensure that whole chromosomes were imputed as one window. After each imputation step, SNPs with an allelic ${R}^{2}$ < 0.7 were removed. Imputation to the sequence level was carried out by using default parameters except for window sizes which were set at 50,000 SNPs. The overall median imputation allelic ${R}^{2}$ for the wider set of 153,357 animals was 0.986, the same value for the set of 38,085 animals included in this study.

### Genome-wide association studies

Separate GWAS were conducted using the Bolt-LMM software [52] for each of the 898 pre-adjusted phenotypes that included the 895 FT-MIR wavenumber phenotypes and three FT-MIR predicted milk composition traits, namely, fat, lactose and protein concentrations (FP, LP, and PP). In total, 17,873,880 imputed sequence variants were included in each GWAS after applying a MAF threshold of 0.1%, based on allele frequencies in the study population of 38,085 animals. Mixed model association statistics were evaluated under an infinitesimal model (as defined by the Bolt-LMM software) to assess the additive effect of each SNP. A genomic relationship matrix (GRM) based on a subset of 43,851 SNPs was simultaneously fitted to account for population structure. That subset of SNPs was derived by filtering the 50 k SNP-chip imputation reference (previously described) to exclude SNPs with a MAF lower than 0.1%. To avoid proximal contamination, a leave-one-segment-out (LOSO) approach was used in the GWAS, with segments of 5 Mbp used to subdivide the autosomes. A conservative Bonferroni significance threshold was used, which considered all tests across the 898 traits and 17,873,880 variants as independent. Based on a genome-wide threshold of α = 0.01, the nominal *p*-value was 6.2e-13 and the corresponding Bonferroni threshold was –log_10_(6.2e-13) = 12.21. The proportion of phenotypic variance explained by each SNP was evaluated as $\frac{{2pqa^{2} }}{{\sigma _{t}^{2} }}$ where $p$ is the frequency of the minor allele, $q = 1 - p$, $a$ is the estimated allele substitution effect, and $\sigma _{t}^{2}$ is the total phenotypic variance. Similarly, the proportion of genetic variance accounted for by each SNP was evaluated as $\frac{{2pqa^{2} }}{{\sigma _{g}^{2} }}$ where $\sigma _{g}^{2}$ is the estimated genetic variance according to SNP-based estimates generated by the Bolt-LMM software.

To distinguish between multiple QTL segregating within the same region of a chromosome, an iterative conditional approach was undertaken for each phenotype. After running an initial GWAS that we refer to as the ‘base GWAS’, chromosomes with a significant *p*-value based on the Bonferroni threshold were identified; and for each of these chromosomes, the most significant variant was identified and added to the set of covariates included in the next iteration. These subsequent iterations were only conducted on chromosomes that retained significant effects, whereby the process was repeated until these analyses ceased to highlight significant effects. For each of these iterations, the set of 43,851 SNPs representing genomic relationships continued to be fitted (using the LOSO approach) to account for population structure. These analyses resulted in a list of variants for each phenotype that aimed at capturing all the significant association analysis signals.

### Gene expression phenotypes and eQTL identification

Gene expression phenotypes and the resulting eQTL were generated as part of a previously described study [36]. Briefly, tissue from 411 cows was used to conduct high-depth mammary RNA-seq, yielding approximately 89 million read pairs per sample. Reads were mapped to the UMD3.1 *Bos taurus* reference genome using the Tophat2 program (version 2.0.12) [53], and filtered to remove outliers based on a principal components analysis of the gene expression values. Additional filters were applied to remove animals with excessively low call rates, and those with genotypes that were not concordant with sire or dam genotypes. This resulted in a dataset containing 357 animals, 62 of which were in common with the 38,085 animals in the current study. Transformed gene expression phenotypes for genes overlapping 1-Mb windows of whole-genome sequences were used to identify significant eQTL [36]. Genetic impacts on gene expression were evaluated by fitting a generalised least-squares model that assessed the relationship between genotype and transformed gene expression phenotypes, with covariances between animals accounted for by the numerator relationship ($\mathbf{A}$) matrix. Resulting χ^2^ statistics with 1 degree of freedom were used to identify eQTL *p*-values. The Bonferroni significance threshold had been set at –log_10_(2.53e-07), based on α = 0.05, corrected for 197,338 tests.

### Identification of protein-coding variants and co-localized eQTL

Whole-genome sequence resolution genotypes within a 1-Mbp window were annotated using the SnpEff software (version 4.1d; build 2015–04-13) [54] and Ensembl UMD3.1.78 gene annotations, to assess the candidacy of each wavenumber and predicted-trait QTL from the iterative GWAS. To focus on the most plausible candidates, variants in QTL regions were filtered to include only those in high linkage disequilibrium (LD) (${R}^{2}$ > 0.9) with a putative impact variant (PIV), where we have defined a PIV as being a splice region variant, or a moderate or high impact coding variant, according to the SnpEff classification. For variants in QTL regions that met these criteria, emphasis was placed on those with ‘highly significant’ effects. That is, the correlation between the PIV and the QTL was in the range (0.975, 1] and the –log_10_(*p*-value) for the effect was greater than 1.5 × the Bonferroni threshold; or the correlation between the PIV and the QTL was in the range (0.95, 0.975] and the –log_10_(*p*-value) for the effect was greater than 2 × the Bonferroni threshold; or the correlation between the PIV and the QTL was in the range (0.925, 0.95] and the –log_10_(*p*-value) for the effect was greater than 2.5 × the Bonferroni threshold. All other variants in QTL regions where the correlation between the PIV and the QTL was higher than 0.9, and the –log_10_(*p*-value) for the effect was greater than the Bonferroni threshold, were classified as ‘moderately significant’.

Wavenumber and predicted-trait QTL were scrutinized to identify co-localized eQTL, following the methodology of Lopdell et al*.* [36]. This approach compares association statistics from the trait QTL to association statistics from variants in the same interval for an eQTL mapping to the same general locus, with the expectation that trait QTL underpinned by eQTL will have common top-associated variants, and/or will have similar patterns of association across the wider spectrum of variants within that interval. Briefly, for each QTL from the iterative GWAS, any significant, pre-computed eQTL within the same 1-Mbp window were identified. To identify cases where trait and expression QTL shared the same top-associated variant, LD criteria were used to highlight tag variants that, at ${R}^{2}$ > 0.9, were linked to the most significant, co-localized eQTL variant. To assess commonalities of association within the broader interval (i.e. beyond pairwise analysis of the top-associated trait QTL/eQTL tag variants), Pearson correlation coefficients between the log-scaled *p*-values of the trait QTL and all eQTL within the interval of interest were computed. To account for regional differences in LD structure, Pearson correlation coefficients were evaluated across the entire 1-Mbp region of interest, and a smaller 500-kbp region, with the strongest correlation used to assess the relationship between the trait and expression QTL *p*-values. Trait QTL were filtered to those for which the Pearson correlation from either window was higher than 0.7.

### FT-MIR wavenumber association effect patterns for genes of interest

After conducting GWAS across FT-MIR wavenumbers, wavenumber QTL that were in strong LD with a PIV, or had a co-localized eQTL (as described in detail above) were identified. In cases where there were multiple candidate genes implicated for a QTL, the gene with a PIV in highest LD with the QTL was selected as representative of the locus. Where multiple loci were implicated for the same gene, the variant in highest LD with either the corresponding PIV or the top variant of the eQTL was used. For the identified genes, the –log_10_(*p*-values) for the representative tag variant were compiled across FT-MIR wavenumbers, creating significance ‘profiles’ that allowed patterns of association across the mid-infrared region to be compared between loci. To facilitate these comparisons and account for differences in *p*-value magnitudes between loci, the –log_10_(*p*-values) were scaled to sum to unity. Differences between scaled significance profiles for loci were evaluated based on the Euclidean distance between corresponding points on the profiles for pairs of genes, and clustering of the distances based on the largest pairwise dissimilarity across elements was performed using the hclust function in R (v4.0.2) [55] with default parameters.

## Results

### Sequence-based genome-wide association analysis

**Fig. 1:**
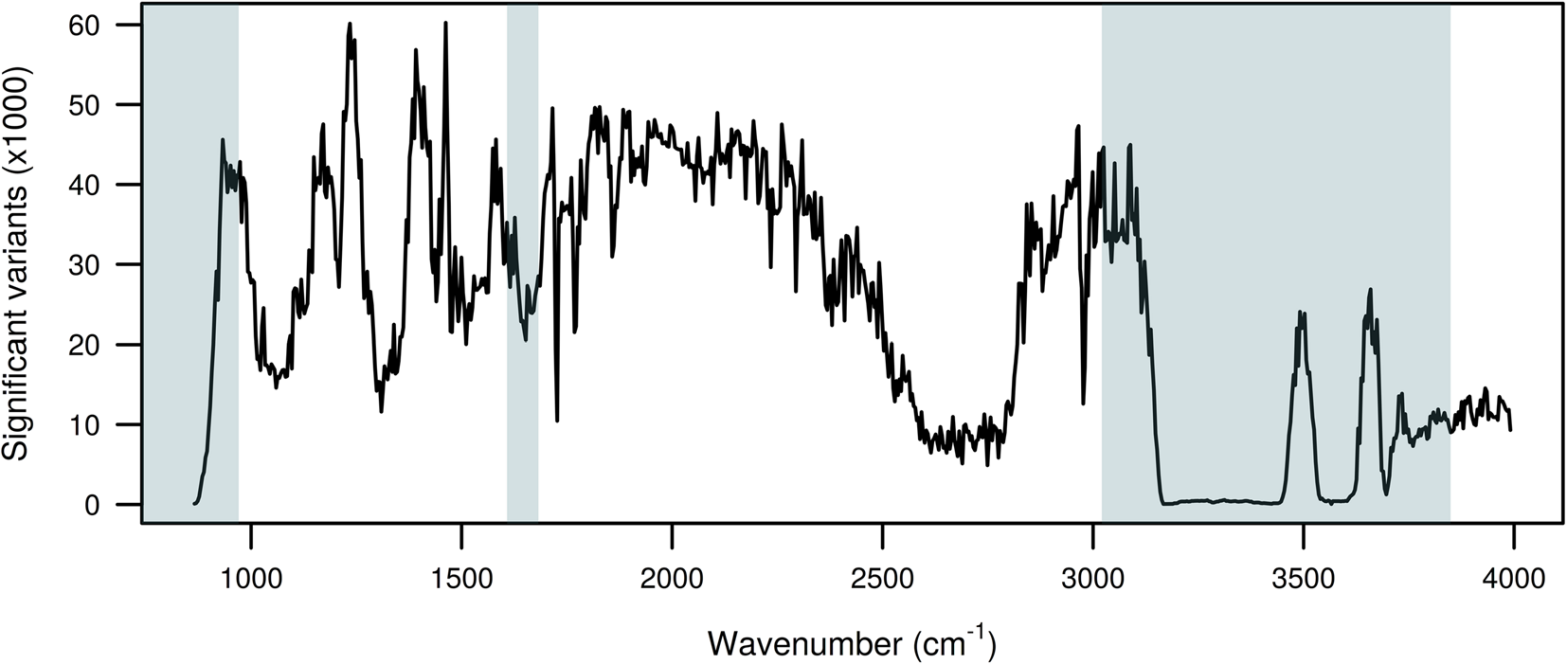
Number of significant variants from GWAS for each individual FT-MIR wavenumber. Noise regions (blue) with low repeatability are defined as from 649 to 970 cm^−1^, from 1608 to 1682 cm^−1^, and from 3021 to 3849 cm^−1^

The first-round pre-iteration (base) GWAS, including 17,873,880 imputed sequence variants, resulted in significant associations for 37,779 variants for FP, 17,159 variants for LP, and 36,067 variants for PP. The number of significant associations for individual FT-MIR wavenumbers ranged from 50 to 60,242, with a mean and median of 24,505 and 25,895 variants, respectively. For 18 of the 895 individual wavenumber phenotypes, the Bolt-LMM GWAS did not converge, due to insufficient genetic variation in the trait. Among the remaining wavenumbers, 830 had at least one significant association in the base GWAS. The numbers of significant variants in the base GWAS for individual wavenumbers across the mid-infrared range are shown in Fig. 1. Regions of the spectrum associated with low signal-to-noise ratios and poor sample measurement repeatability, due to the water content in milk are shaded in blue, according to the definitions in Tiplady et al*.* [44]. Significant associations were identified across most of the spectrum, including within regions that were commonly associated with low signal-to-noise ratios. Among the significant associations observed, 17.0% were positioned within the first 3 Mbp of chromosome 14, which encompasses the *DGAT1* gene that has been widely reported as impacting many milk composition traits [56, 57]. For the FP and PP phenotypes, the proportion of significant associations that were positioned within the first 3 Mbp of chromosome 14 were 16.5% and 13.6%, respectively. None of the significant associations for the LP phenotype localized to that region.

**Fig. 2:**
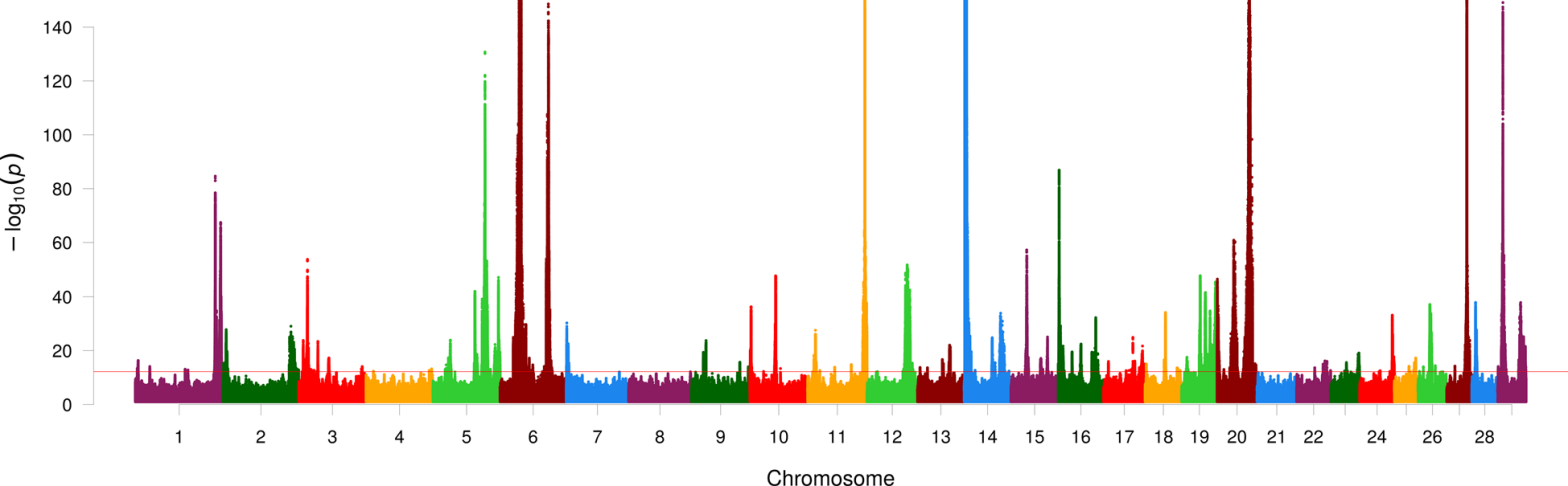
Manhattan plot showing association effects for FT-MIR wavenumbers. Consolidated association effects shown for FT-MIR wavenumbers. Chromosomes and genomic positions based on the UMD3.1 *Bos taurus* reference genome are represented on the x-axis. The strength of association signals is represented as the −log_10_(*p*-value) on the y-axis which has been truncated to facilitate visualisation of the results. The horizontal red line shows the Bonferroni significance threshold of −log_10_(6.3e–13)

**Fig. 3:**
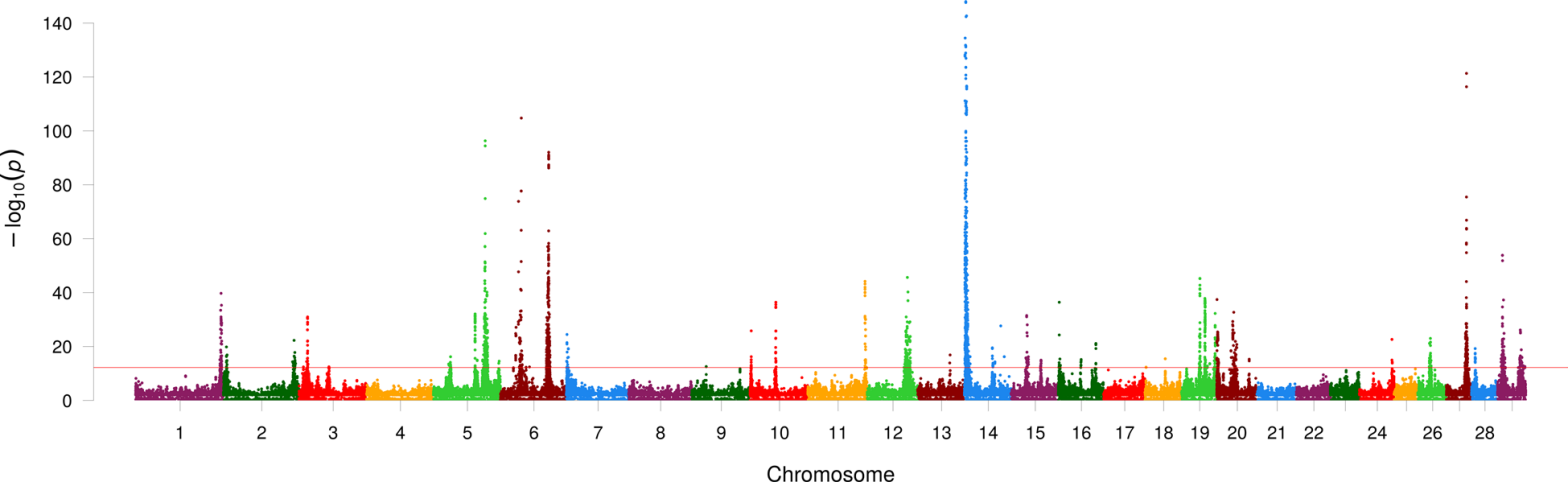
Manhattan plot showing association effects for FT-MIR predicted milk composition traits. Consolidated association effects shown for FT-MIR predicted milk production traits (Fat %, Lactose % and Protein %). Chromosomes and genomic positions based on the UMD3.1 *Bos taurus* reference genome are represented on the x-axis. The strength of association signals is represented as the −log_10_(*p*-value) on the y-axis which has been truncated to facilitate visualisation of the results. The horizontal red line shows the Bonferroni significance threshold of −log_10_(6.3e–13)

In the base GWAS, individual FT-MIR wavenumber QTL were observed on 27 of the 29 bovine autosomes (Fig. 2) within 450 different 1-Mbp regions. In contrast, QTL for FT-MIR predicted milk composition traits were observed on 25 of the 29 autosomes (Fig. 3) within 246 different 1-Mbp regions. The number of iterations required after the base GWAS until the analyses ceased to highlight significant effects for the FT-MIR wavenumber phenotypes ranged from 0 to 10, with an average of 3.9. For the FT-MIR predicted milk composition traits, FP, LP and PP, the number of iterations required after the base GWAS was 6, 8 and 7, respectively. For the FT-MIR wavenumber phenotypes, all significant signals were captured by no more than 68 tag variants, with the mean and median number of tag variants required to capture the signal for an individual wavenumber being 26 and 29, respectively. For FT-MIR predictions of FP, LP and PP, all significant signals were captured by 55, 72 and 86 tag variants, respectively.

### Identification of candidate causative variants

To identify candidate causative variants for wavenumber and predicted-trait QTL, we used functional annotation to find PIV in strong LD (${R}^{2}$ > 0.9) with trait QTL from the GWAS iterations. Those criteria yielded 42 1-Mbp regions, encompassing 55 effects with a PIV for at least one FT-MIR wavenumber. Based on our categorisation of signals into moderately and highly significant groups, 31 of the 55 wavenumber QTL were classified as highly significant. Details of these 31 effects are in Table 1. Manhattan plots of a 1-Mbp region centred on the QTL tag variant for each of the 31 highly significant wavenumber QTL from the base GWAS are provided (see Additional file 1: Figure S1). Details of the wavenumber QTL classified as moderately significant are in Table S2 (see Additional file 2: Table S2). Note that there are three effects where the locus has been identified as highly significant based on the LD with one or more other loci (Table 1), and moderately significant based on the LD with other loci (see Additional file 2: Table S2). Effect sizes and MAF details for the tag SNP of the 31 highly significant wavenumber QTL are in Table S3 (see Additional file 2: Table S3). For each of these 31 QTL, the proportions of phenotypic and genetic variance that they account for across FT-MIR wavenumber and predicted composition traits are in Table S4 (see Additional file 2: Table S4). Of the 31 highly significant wavenumber QTL, 14 were identified in the base GWAS (Iteration 0). For the 17 highly significant wavenumber QTL identified in subsequent GWAS iterations after the base GWAS (Table 1), *p*-values at previous iterations for the phenotype, and *p*-values for the corresponding top chromosomal SNP in that iteration are in Table S5 (see Additional file 2: Table S5).

**Table 1:** Peak variants for FT-MIR wavenumbers with highly significant protein sequence association effects

Chr	Position	Tag variant ID	N of hits	Top wvn (cm^−1^)	Iter	*P*-value	Protein coding variant ID	LD	Gene	Impact	Description
3	7908611	rs137763930	11	940	1	6.7e−20	rs110560331	0.976	*FCRLA*	L	c.233-3 T > C
3	7931694	rs211402696	20	1462	2	1.2e−23	rs381714237	0.989	*FCGR2B*	H	c.899dupC
3	15411459	rs134900385	6	1022	1	4.3e−19	rs382689947	0.994	*FAM189B*	M	c.1237 T > C
3	15411459	rs134900385	6	1022	1	4.3e−19	rs134844772	0.990	*GBA*	M	c.1080C > A
3	15411459	rs134900385	6	1022	1	4.3 e−19	rs132659643	0.999	*HCN3*	M	c.1699A > G
3	15411459	rs134900385	6	1022	1	4.3e−19	rs109330809	0.990	*MTX1*	L	c.508-6 T > C
3	15517871	rs109328483	6	1007	1	4.4e−19	rs136761456	0.992	*SCAMP3*	M	c.151G > C
3	15517871	rs109328483	6	1007	1	4.4e−19	rs43706482	0.994	*THBS3*	L	c.2075-3 T > C
3	15550598	rs380597285	327	1462	0	1.3e−54	rs109816684	0.994	*SLC50A1*	L	c.282 + 7G > A
5	75729880	rs384734208	50	1466	1	5.0e−47	rs207628090	0.930	*CSF2RB*	M	c.41 T > C
5	75758989	rs210094995	2	1447	0	5.8e−40	rs210937722	0.926	*NCF4*	M	c.841G > C
5	118246868	rs136859160	308	1261	0	3.0e−44	rs456403270	0.937	*TBC1D22A*	M	c.1063C > T
6	38027010	rs43702337	455	1119	0	7.3e−948	rs43702337	1	*ABCG2*	M	c.1742A > C
6	87181619	rs43703011	17	3633	2	2.5e−22	rs43703011	1	*CSN2*	M	c.245C > A
6	87274397	rs378808772	3	1283	2	9.9e−51	rs43703010	0.974	*CSN1S1*	M	c.620A > G
6	87390576	rs43703015	18	1473	1	4.0e−108	rs43703015	1	*CSN3*	M	c.470 T > C
11	103304757	rs109625649	329	1593	0	1.2e−134	rs109625649	1	*PAEP*	M	c.401 T > C
11	104242578	rs207688357	11	1462	0	5.5e−33	rs207688357	1	*ABO*	H	c.233 + 1G > C
12	69612955	rs383509255	132	1716	0	6.4e−45	rs208744187	0.950	*TGDS*	M	c.204A > C
14	1726650	rs133611586	6	3514	1	1.6e−75		0.992	*WDR97*	L	c.2656-5_2656-4insG
14	1732043	rs437406031	384	2846	1	6.3e−42	rs450710918	0.990	*ENS..39978*	M	c.352G > A
14	1732043	rs437406031	384	2846	1	6.3e−42	rs476736066	0.997	*MROH1*	M	c.3549G > C
14	1755742	rs384226556	5	2656	0	4.0e−20	rs209542297	0.9998	*CPSF1*	L	c.4287 T > C
14	1802265	rs109234250	310	1716	0	1.5e−2607	rs109234250	1	*DGAT1*	M	c.694G > A
14	1802265	rs109234250	310	1716	0	1.5e−2607	rs134364612	0.999	*SLC52A2*	M	c.724A > G
14	66328304	rs446084949	19	1029	1	2.7e−20	rs446084949	1	*SPAG1*	M	c.2044G > A
15	28347165	rs210034037	5	1537	0	7.7e−35	rs208325660	0.999	*RNF214*	M	c.314G > A
15	53940444	rs382926661	23	1205	1	4.2e−19	rs380220394	0.993	*DNAJB13*	L	c.69-4 T > C
16	24977696	rs111027377	62	2742	2	4.8e−25	rs109896036	0.988	*MTARC1*	L	c.628-5C > T
16	24977696	rs111027377	62	2742	2	4.8e−25	rs110899826	0.988	*MTARC1*	M	c.581C > G
19	42428366	rs209808022	4	1250	1	3.1e−25	rs209302038	0.991	*KRT9*	M	c.196C > T
19	42488389	rs379667889	8	1447	0	7.8e−34	rs209756857	0.969	*KRT42*	L	c.57 + 7C > T
19	42488389	rs379667889	8	1447	0	7.8e−34	rs383013355	0.963	*KRT16*	M	c.896A > G
19	42488389	rs379667889	8	1447	0	7.8e−34	rs208923483	0.966	*KRT17*	M	c.146G > C
19	42488389	rs379667889	8	1447	0	7.8e−34	rs385937063	0.966	*KRT17*	L	c.1233C > T
19	43036265	rs210324533	11	1029	1	5.3e−43	rs207799702	0.944	*KAT2A*	L	c.700-7C > G
19	43036265	rs210324533	11	1029	1	5.3e−43	rs209410283	0.945	*KCNH4*	M	c.408C > G
19	43036265	rs210324533	11	1029	1	5.3e−43	rs377779402	0.945	*KCNH4*	H	c.2663 + 2 T > C
19	43053995	rs481837688	24	1212	1	6.6e−26	rs481837688	1	*STAT5A*	M	c.2305C > A
19	51303887	rs41921224	65	1499	0	1.9e−35	rs41921160	0.993	*CCDC57*	M	c.1907 T > C
19	57087981	rs41920620	6	1216	0	1.8e−21	rs469721022	0.999	*HID1*	L	c.1147-7G > C
28	6559147	rs133101552	3	1261	0	8.6e−23	rs133101552	1	*KCNK1*	M	c.934C > A
29	41821270	rs207854419	14	1257	1	4.6e−30	rs384900272	0.998	*NXF1*	M	c.1555G > A

Peak variants and association effects for FT-MIR wavenumbers classified as highly significant. Highly significant effects are classified such that: the –log_10_(*p*-value) for the effect was greater than 1.5 × the Bonferroni threshold and the correlation between the tag variant and the protein sequence variant was in the range (0.975, 1]; or the –log_10_(*p*-value) for the effect was greater than 2 × the Bonferroni threshold and the correlation between the tag variant and the protein sequence variant was in the range (0.95, 0.975]; or the –log_10_(*p*-value) for the effect was greater than 2.5 × the Bonferroni threshold and the correlation between the tag variant and the protein sequence variant was in the range (0.925, 0.95]. Bonferroni threshold: –log_10_(6.2e–13). N of hits: number of wavenumbers for which the variant was selected as the representative (most significant) tag variant for a peak. Iterations are defined relative to the base GWAS, with the base GWAS represented as iteration 0. *L* Low impact splice region variant, *M*  Moderate impact missense variant, *H* High impact splice donor

For predicted composition traits, 27 effects with a PIV were identified within 15 1-Mbp regions. Of the 27 predicted-trait QTL, 18 were classified as highly significant. Details of these effects are in Table 2, with details of the QTL classified as moderately significant in Table S6 (see Additional file 2: Table S6). Effect sizes and MAF details for highly significant predicted-trait QTL are in Table S7 (see Additional file 2: Table S7). Details of highly significant predicted-trait QTL from iterations subsequent to the base GWAS in Table S8 (see Additional file 2: Table S8).

**Table 2:** Peak variants for composite milk production traits with highly significant protein sequence association effects

Trait	Chr	Position	Tag variant ID	Iteration	*P*-value	Protein coding variant ID	LD	Gene	Impact	Description
FP	5	75698283	rs385866519	1	4.0e−19	rs207628090	0.979	*CSF2RB*	M	c.41 T > C
FP	11	103304757	rs109625649	0	4.3e−46	rs109625649	1	*PAEP*	M	c.401 T > C
FP	12	69608900	rs211406918	0	4.2e−33	rs208744187	0.951	*TGDS*	M	c.204A > C
FP	14	1732043	rs437406031	1	7.2e−37	rs450710918	0.990	*ENS..39978*	M	c.352G > A
FP	14	1732043	rs437406031	1	7.2e−37	rs476736066	0.997	*MROH1*	M	c.3549G > C
FP	14	1800439	rs209876151	0	8.9e−2225	rs109326954	0.9999	*DGAT1*	M	c.695C > A
FP	14	1800439	rs209876151	0	8.9e−2225	rs134364612	0.9998	*SLC52A2*	M	c.724A > G
LP	3	15433518	rs109749506	1	1.3e−20	rs382689947	0.995	*TENT5A*	M	c.1237 T > C
LP	3	15433518	rs109749506	1	1.3e−20	rs134844772	0.992	*GBA*	M	c.1080C > A
LP	3	15433518	rs109749506	1	1.3e−20	rs109330809	0.992	*MTX1*	L	c.508-6 T > C
LP	3	15545091	rs379353107	0	2.2e−42	rs109816684	0.998	*SLC50A1*	L	c.282 + 7G > A
LP	6	38027010	rs43702337	0	9.0e−717	rs43702337	1	*ABCG2*	M	c.1742A > C
LP	16	24983926	rs110162358	2	1.0e−19	rs109896036	0.999	*MTARC1*	L	c.628-5C > T
LP	16	24983926	rs110162358	2	1.0e−19	rs110899826	0.999	*MTARC1*	M	c.581C > G
LP	19	43036265	rs210324533	3	9.4e−40	rs207799702	0.944	*KAT2A*	L	c.700-7C > G
LP	19	43036265	rs210324533	3	9.4e−40	rs209410283	0.945	*KCNH4*	M	c.408C > G
LP	19	43036265	rs210324533	3	9.4e−40	rs377779402	0.945	*KCNH4*	H	c.2663 + 2 T > C
PP	3	15550598	rs380597285	0	1.7e−37	rs109816684	0.994	*SLC50A1*	L	c.282 + 7G > A
PP	5	75758989	rs210094995	0	3.3e−34	rs209394772	0.935	*CSF2RB*	M	c.227G > A
PP	5	75758989	rs210094995	0	3.3e−34	rs210937722	0.926	*NCF4*	M	c.841G > C
PP	5	118239754	rs384479185	2	3.9e−32	rs456403270	0.976	*TBC1D22A*	M	c.1063C > T
PP	6	38027010	rs43702337	0	6.4e−115	rs43702337	1	*ABCG2*	M	c.1742A > C
PP	14	1763380	rs135017891	0	5.9e−718	rs135258919	0.999	*HSF1*	M	c.1031 T > C
PP	14	1802265	rs109234250	1	1.2e−61	rs109234250	1	*DGAT1*	M	c.694G > A
PP	14	1802265	rs109234250	1	1.2e−61	rs134364612	0.999	*SLC52A2*	M	c.724A > G
PP	15	53940444	rs382926661	1	2.9e−20	rs380220394	0.992	*DNAJB13*	L	c.69-4 T > C
PP	19	43035006	rs209494359	0	1.6e−40	rs207799702	0.944	*KAT2A*	L	c.700-7C > G
PP	19	43035006	rs209494359	0	1.6e−40	rs209410283	0.945	*KCNH4*	M	c.408C > G
PP	19	43035006	rs209494359	0	1.6e−40	rs377779402	0.945	*KCNH4*	H	c.2663 + 2 T > C

Peak variants for composite milk production traits with highly significant protein sequence effects whereby: the –log_10_(*p*-value) for the effect was greater than 1.5 × the Bonferroni threshold and the correlation between the tag variant and the protein sequence variant was in the range (0.975, 1]; or the –log_10_(*p*-value) for the effect was greater than 2 × the Bonferroni threshold and the correlation between the tag variant and the protein sequence variant was in the range (0.95, 0.975]; or the –log_10_(*p*-value) for the effect was greater than 2.5 × the Bonferroni threshold and the correlation between the tag variant and the protein sequence variant was in the range (0.925, 0.95]. Bonferroni threshold: –log_10_(6.2e–13). Iterations are defined relative to the base GWAS, with the base GWAS represented as iteration 0. *FP* Fat %, *LP* Lactose %, *PP* Protein %, *L* Low impact splice region variant, *M* Moderate impact missense variant, *H* High impact splice donor

**Fig. 4:**
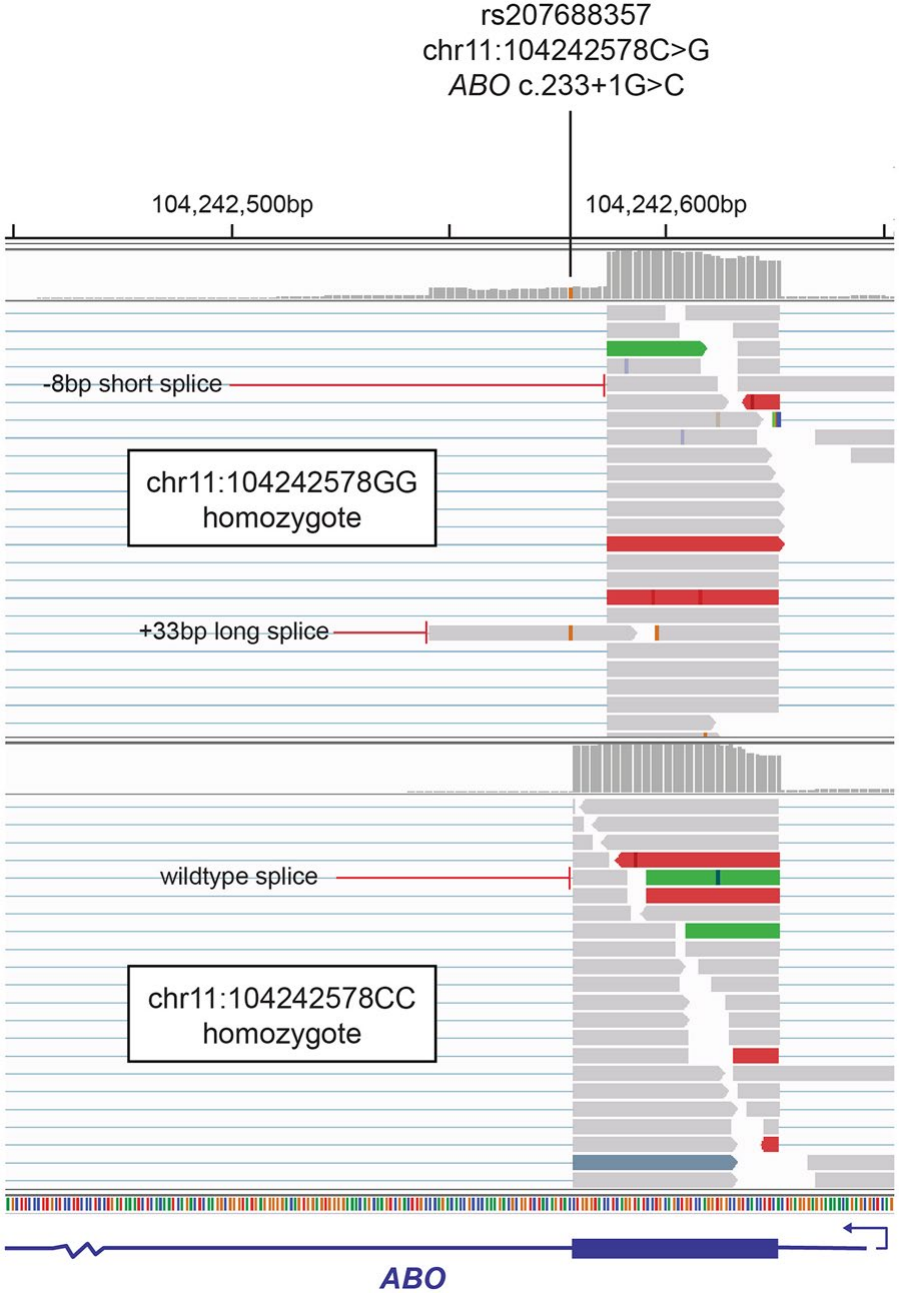
Mammary RNA-seq alignments representing ABO intron/exon 5 splicing structures of the chr11:104242578GG and chr11:104242578CC genotypes. The site of the proposed chr11:104242578C > G essential splice donor SNP is indicated, with individual reads and coverage data showing alternate splice forms in the animal carrying the mutation. This coverage track also represents the cryptic, ‘ + 33 bp long splice’ transcript as the minority splice form relative to the ‘− 8 bp short splice’ transcript, the former representing an in-frame variant, with the latter causing a predicted frameshifted protein isoform

Of all candidate protein coding mutations identified, we were particularly interested in those identified as having a high impact according to the SnpEff classification, in which variants that are expected to strongly disrupt or ablate gene function could a priori be considered as excellent candidates for these QTL. Three such PIV from the wavenumber and predicted-trait QTL fit this definition, comprising frameshift mutations in the *FCGR2B* or *KCNH4* genes, and a splice donor mutation in the *ABO* gene (Tables 1 and 2). Since this class of variants was likely to be enriched for annotation errors [58], we manually visualized mammary RNA-seq alignments for these mutations to help confirm their predicted impacts as disruptive of coding sequences. Although the *FCGR2B* rs381714237 variant was represented in the RNA-seq reads, the mutation appeared to be intronic. Annotation of the *KCNH4* mutation appeared similarly dubious, with limited evidence suggesting that it was localized in a mammary-expressed exon. The *ABO* rs207688357 mutation was clearly localized in the donor site of the splice junction of intron/exon 5, with animals that carried the mutation showing activation of cryptic alternative splice sites. These alternative transcripts comprised an 8-bp contraction, or 33-bp expansion of exon 5 (splicing at chr11:104242578 and chr11:1042425462 respectively, Fig. 4), which suggests that the ABO protein in animals homozygous for the mutation is non-functional.

### Identification of co-localized eQTL

Comparisons of association statistics from trait QTL to those representing mammary eQTL variants in the same interval identified co-localized eQTL for 38 wavenumber QTL (see details in Table 3). For 19 of these identified from the base GWAS (Iteration = 0), Manhattan plots are provided for 1-Mbp regions centred on the trait QTL tag variant (see Additional file 3: Figure S9). Effect sizes and MAF details for all 38 loci with a co-localized trait QTL and eQTL pair are in Table S10 (see Additional file 4: Table S10). For each of these 38 loci, the proportions of phenotypic and genetic variance explained across FT-MIR wavenumber and predicted composition traits are in Table S11 (see Additional file 4: Table S11). For the 19 trait QTL identified in subsequent GWAS iterations after the base GWAS, *p*-values at previous iterations for the phenotype, and *p*-values for the corresponding top chromosomal SNP in that iteration are in Table S12 (see Additional file 4: Table S12).

**Table 3:** Peak variants for FT-MIR wavenumbers with co-localized eQTL

Chr	Position	Tag variant ID	Iter	Top wvn (cm^−1^)	N of hits	*P*-value	Gene	Top eQTL variant ID	Top eQTL variant *p*-value	LD	Pearson	Pearson window (Mb)
1	5120248	rs42317521	2	2794	55	4.0e−18	*CLDN8*	rs42317521	4.3e−17	1	0.764	0.5
1	144377960	rs208161466	0	2592	22	2.3e−85	*SLC37A1*	rs208161466	4.1e−15	1	0.710	1.0
1	146481250	rs383691757	1	1071	1	4.7e−15	*CSTB*	rs210595016	1.4e−52	0.992	0.938	0.5
1	154125158	rs207836083	0	1130	142	3.6e−68	*SH3BP5*	rs207836083	2.6e−32	1	0.816	0.5
3	15411459	rs134900385	1	1022	6	4.3e−19	*KRTCAP2*	rs133285846	9.7e−09	0.996	0.938	1.0
3	15550598	rs380597285	0	1462	325	1.3e−54	*SLC50A1*	rs380597285	8.7e−16	1	0.854	0.5
3	34387618	rs109030498	1	1466	157	3.5e−25	*ELAPOR1*	rs109030498	3.2e−30	1	0.817	0.5
3	53755929	rs209271975	1	1089	15	8.6e−22	*LRRC8C*	rs466686834	3.5e−39	0.99	0.927	0.5
5	75729880	rs384734208	1	1466	44	5.0e−47	*CSF2RB*	rs210641868	9.2e−27	0.926	0.752	1.0
5	75732526	rs210305241	1	1458	5	4.4e−42	*NCF4*	rs209273109	9.3e−16	0.91	0.813	1.0
5	93945738	rs211210569	0	1171	544	1.8e−131	*MGST1*	rs209372883	3.2e−43	0.919	0.925	1.0
6	46568418	rs210515595	3	1772	5	7.7e−22	*SLC34A2*	rs110805476	2.5e−07	0.979	0.805	0.5
6	87388064	rs379473589	1	1436	17	1.1e−97	*CSN3*	rs208009847	9.9e−33	0.963	0.878	0.5
9	21637056	rs209222932	0	1003	33	9.4e−20	*YRMY5A*	rs209222932	2.8e−36	1	0.634	0.5
9	26534109	rs208123385	0	1462	36	1.9e−24	*RNF217*	rs208173647	1.3e−16	0.986	0.856	0.5
9	87585031	rs110986237	1	1470	6	7.4e−15	*TAB2*	rs110986237	9.5e−12	1	0.851	0.5
9	102874726	rs137238900	0	1768	1	1.0e−14	*MPC1*	rs134094426	6.9e−15	0.969	0.849	0.5
10	46581015	rs109326466	0	1246	15	2.0e−46	*USP3*	rs109326466	2.0e−31	1	0.961	0.5
11	14180010	rs110527112	1	2760	23	3.6e−29	*XDH*	rs207554031	8.8e−26	0.978	0.709	0.5
11	78868975		1	1112	10	1.2e−19	*LAPTM4A*	rs110552157	1.3e−40	0.998	0.920	0.5
11	103292402	rs383398415	0	2548	1	3.5e−56	*PAEP*	rs109333988	1.2e−29	0.933	0.956	0.5
11	104229609	rs110534892	0	3648	10	1.2e−21	*ABO*	rs109750996	3.9e−28	0.944	0.803	0.5
14	1754287	rs135443540	0	1085	3	1.6e−39	*DGAT1*	rs137202508	8.9e−42	0.905	0.944	0.5
15	57266467	rs136337092	0	3935	1	2.7e−13	*CAPN5*	rs136208815	9.3e−46	0.997	0.940	0.5
16	66314547	rs42579412	2	1425	1	1.0e−15	*RGL1*	rs42579412	6.3e−14	1	0.727	0.5
16	67730371	rs380453838	1	1757	125	3.8e−21	*IVNS1ABP*	rs380453838	4.5e−27	1	0.876	0.5
18	2203322	rs132899112	1	1466	7	1.4e−15	*FA2H*	rs137235970	1.9e−27	0.998	0.875	0.5
19	33517487	rs434248431	0	1100	23	2.9e−46	*PMP22*	rs434248431	8.6e−38	1	0.832	0.5
19	43036265	rs210324533	1	1029	11	5.3e−43	*GHDC*	rs381442991	1.8e−22	0.945	0.975	0.5
19	57079881	rs381175117	2	1220	9	2.0e−23	*HID1*	rs109407913	1.2e−32	0.936	0.803	0.5
19	61134515	rs41923843	0	1130	45	3.2e−46	*KCNJ2*	rs41923843	1.7e−26	1	0.882	0.5
20	58454531	rs135636613	0	1391	23	4.3e−441	*ANKH*	rs135636613	2.4e−16	1	0.860	0.5
22	53519865	rs109233889	0	1235	7	5.3e−15	*LTF*	rs109233889	1.3e−32	1	0.813	0.5
24	58817202	rs208779762	0	1220	23	6.8e−34	*LMAN1*	rs207893260	1.3e−27	0.958	0.713	1.0
27	36211708	rs209855549	0	1731	157	6.2e−188	*GPAT4*	rs209855549	3.7e−21	1	0.848	0.5
27	41267242	rs109068627	1	2977	23	3.5e−26	*THRB*	rs109068627	1.7e−22	1	0.704	0.5
29	9546217	rs380868305	0	1130	8	4.6e−186	*PICALM*	rs380868305	2.4e−54	1	0.831	0.5
29	44579245	rs439384463	2	1548	3	4.3e−16	*MUS81*		3.0e−21	0.924	0.913	0.5

Peak variants for FT-MIR wavenumbers with a co-localized eQTL. Co-localized eQTL are defined such that: the Pearson correlation between the –log_10_(*p*-values) of the trait QTL and the –log_10_(*p*-values) of the eQTL is higher than 0.7; and the LD between the tag variant for the trait QTL and the top eQTL variant is higher than 0.9. The Pearson correlation shown is the highest from two different size windows (0.5 Mbp and 1 Mbp), centred on the top tag variant. Iterations are defined relative to the base GWAS, with the base GWAS represented as iteration 0. N of hits: number of wavenumbers for which the variant was selected as the representative (most significant) tag variant for a peak

Co-localized eQTL were identified for 25 predicted-trait QTL. Details of these trait QTL and eQTL pairs are in Table 4, with effect sizes and MAF details provided in Table S13 (see Additional file 4: Table S13). Further details of the 12 QTL identified in iterations subsequent to the base GWAS are in Table S14 (see Additional file 4: Table S14).

**Table 4:** Peak variants for composite milk production traits with co-localized eQTL

Trait	Chr	Position	Tag variant ID	Iteration	*P*-value	Gene	Top eQTL variant ID	Top eQTL variant *p*-value	LD	Pearson	Pearson window (Mbp)
FP	3	34387618	rs109030498	2	6.0e−13	*ELAPOR1*	rs109030498	3.2e−30	1	0.832	0.5
FP	5	75698283	rs385866519	1	4.0e−19	*CSF2RB*	rs210641868	9.2e−27	0.910	0.701	1
FP	5	93945738	rs211210569	0	6.7e−106	*MGST1*	rs209372883	3.2e−43	0.919	0.928	1
FP	10	46483019	rs133089336	0	4.5e−13	*USP3*	rs208181306	2.0e−31	0.909	0.905	0.5
FP	11	104229609	rs110534892	1	2.6e−14	*ABO*	rs109750996	3.9e−28	0.944	0.727	1
FP	16	67730371	rs380453838	1	2.6e−19	*IVNS1ABP*	rs380453838	4.5e−27	1	0.886	0.5
FP	27	36211708	rs209855549	0	9.7e−132	*GPAT4*	rs209855549	3.7e−21	1	0.819	0.5
LP	1	154122887	rs42167460	0	1.2e−50	*SH3BP5*	rs380642859	2.6e−32	0.999	0.859	0.5
LP	3	15433518	rs109749506	1	1.3e−20	*KRTCAP2*	rs133285846	9.7e−09	0.995	0.940	1
LP	3	15545091	rs379353107	0	2.2e−42	*SLC50A1*	rs379353107	8.7e−16	1	0.806	0.5
LP	3	53994057	rs211488591	2	6.7e−18	*LRRC8C*	rs466686834	3.5e−39	0.986	0.753	1
LP	19	43036265	rs210324533	3	9.4e−40	*GHDC*	rs381442991	1.8e−22	0.945	0.963	0.5
LP	19	61134515	rs41923843	1	1.1e−46	*KCNJ2*	rs41923843	1.7e−26	1	0.857	0.5
LP	20	58448763	rs134813825	0	3.2e−18	*ANKH*	rs134813825	2.4e−16	1	0.809	0.5
LP	27	36204066	rs208306200	0	1.9e−21	*GPAT4*	rs208306200	3.7e−21	1	0.767	0.5
LP	29	9577372	rs380473328	0	2.1e−140	*PICALM*	rs384691767	2.4e−54	0.996	0.845	0.5
PP	3	15520971	rs109098377	2	7.5e−16	*KRTCAP2*	rs133285846	9.7e−09	0.989	0.928	0.5
PP	3	15550598	rs380597285	0	1.7e−37	*SLC50A1*	rs380597285	8.7e−16	1	0.832	0.5
PP	5	75680825	rs208925020	4	8.5e−23	*CSF2RB*	rs210641868	9.2e−27	0.947	0.871	1
PP	5	93945738	rs211210569	1	3.7e−42	*MGST1*	rs209372883	3.2e−43	0.919	0.817	0.5
PP	6	87387870	rs382652853	2	2.9e−45	*CSN3*	rs208009847	9.9e−33	0.963	0.891	0.5
PP	10	46581015	rs109326466	0	4.0e−38	*USP3*	rs109326466	2.0e−31	1	0.961	0.5
PP	18	2203,325	rs135350753	0	2.1e−13	*FA2H*	rs137235970	1.9e−27	0.997	0.831	0.5
PP	19	43035006	rs209494359	0	1.6e−40	*GHDC*	rs381442991	1.8e−22	0.945	0.976	0.5
PP	24	58817202	rs208779762	0	5.7e−26	*LMAN1*	rs207893260	1.3e−27	0.958	0.737	0.5

Peak variants for composite milk production traits with a co-localized eQTL. Co-localized eQTL are defined such that: the Pearson correlation between the –log_10_(*p*-values) of the trait QTL and the –log_10_(*p*-values) of the eQTL is higher than 0.7; and the LD between the tag variant for the trait QTL and the top eQTL variant is higher than 0.9. The Pearson correlation shown is the highest from two different size windows (0.5 Mbp and 1 Mbp), centred on the top tag variant. Iterations are defined relative to the base GWAS, with the base GWAS represented as iteration 0. *FP* Fat %, *LP*  Lactose %, *PP* Protein %

### Investigation of patterns of FT-MIR wavenumber associations for genes of interest

**Fig. 5:**
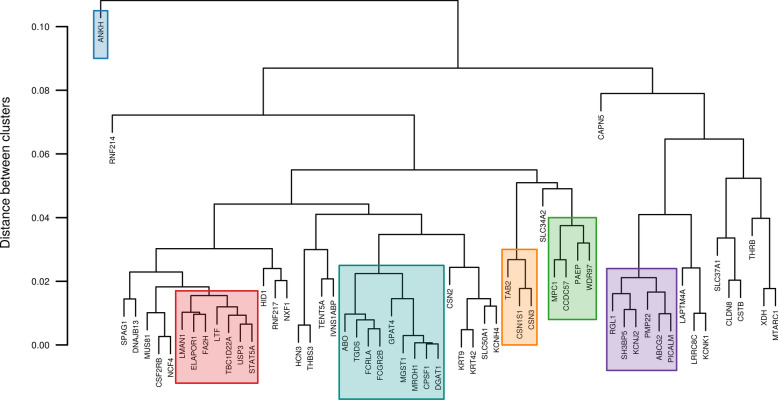
Gene clusters for significance profiles of tag variants representing candidate genes. Gene clusters based on the Euclidean distance between pairs of log-scaled *p*-value profiles across the mid-infrared spectrum for tag variants. Significance profiles for the highlighted gene clusters are presented in Figs. 6 and 7

In total, 70 genes were implicated whereby the tag locus of the wavenumber QTL was in high LD with a PIV (Table 1), or in high LD with the top variant of a co-localized eQTL (Table 3). In cases where multiple candidate genes were implicated for a QTL, the gene with the PIV in highest LD with the QTL tag variant was used to represent the locus. This resulted in tag loci representing 59 genes, for which scaled significance profiles were generated to represent their association patterns across the mid-infrared region. Clustering analysis based on the largest pairwise dissimilarity between corresponding points on profiles for pairs of genes resulted in > 20 clusters (Fig. 5). Significance profiles for all 59 genes are provided in Figure S15 (see Additional file 5: Figure S15).

**Fig. 6:**
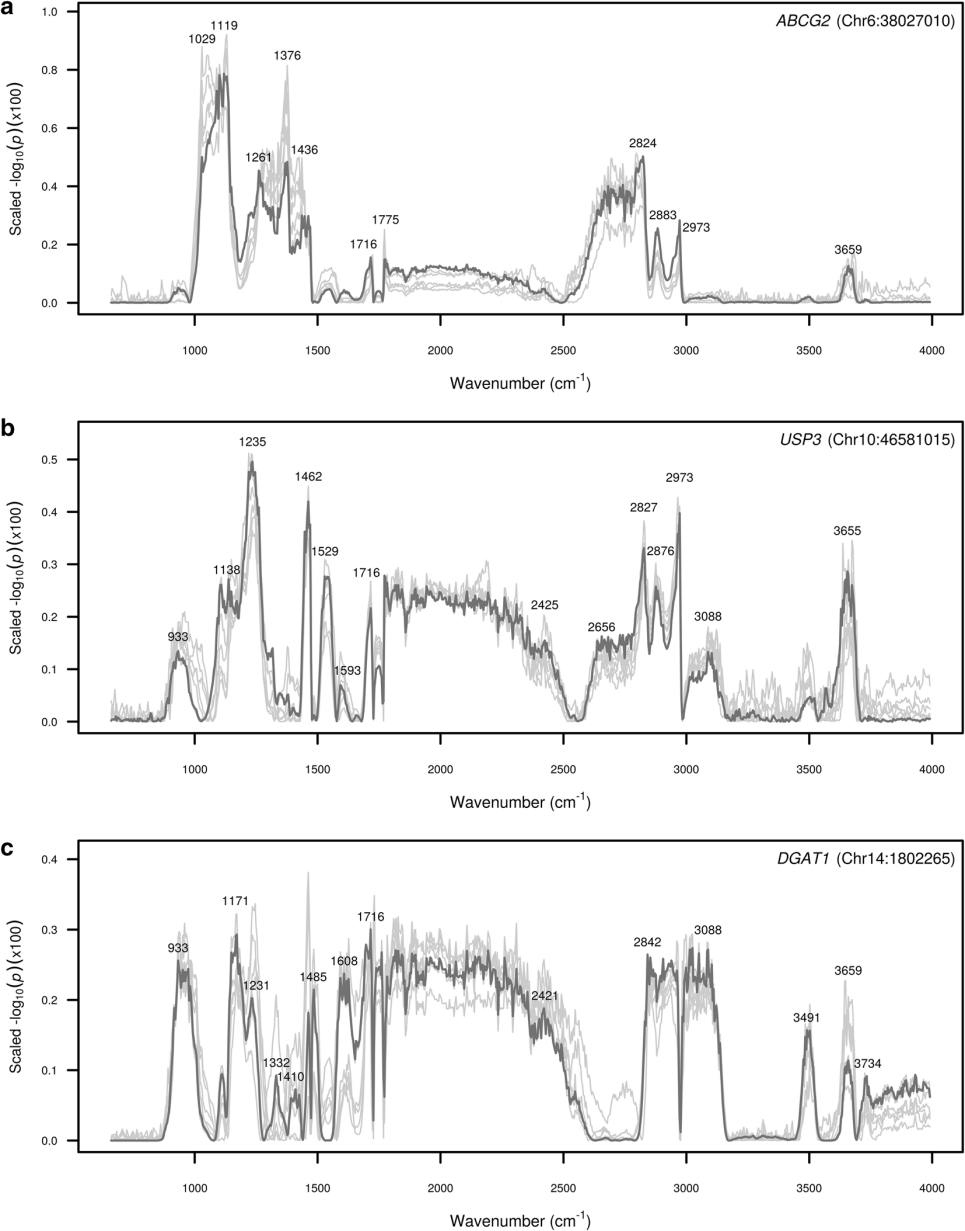
Significance profiles across the mid-infrared spectrum for tag variants of candidate genes within gene clusters. Y-axis values represent the strength of association signals as the −log_10_(*p*-value) of the effect, scaled to sum to unity across the mid-infrared spectral range. The significance profile for the most highly associated tag variant is shown in dark grey with the profiles for the other genes within the cluster shown in light grey: **a** *ABCG2* (Chr6:38027010; dark grey), *SH3BP5* (Chr1:154125158), *RGL1* (Chr16:66314547), *PMP22* (Chr19:33517487), *KCNJ2* (Chr19:61134515), *PICALM* (Chr29:9546217); **b** *USP3* (Chr10:46581015; dark grey), *ELAPOR1* (Chr3:34387618), *TBC1D22A* (Chr5:118246868), *FA2H* (Chr18:2203322), *STAT5A* (Chr19:43053995), *LTF* (Chr22:53519865), *LMAN1* (Chr24:58817202); and **c** DGAT1 (Chr14:1802265; dark grey), *FCRLA* (Chr3:7908611), *FCGR2B* (Chr3:7931694), *MGST1* (Chr5:93945738), *ABO* (Chr11:104242578), *TGDS* (Chr12:69612955), *MROH1* (Chr14:1732043), *CPSF1* (Chr14:1755742), *GPAT4* (Chr27:36211708)

Significance profiles for a subset of gene clusters from Fig. 5 are presented in Fig. 6. For each cluster, the significance profile for the gene with the largest QTL is shown in dark grey with the profiles for other genes within the cluster (according to highlighted clusters in Fig. 5) shown in light grey. Significance profiles varied widely between clusters, but were highly consistent within clusters. The first cluster (Fig. 6a) includes genes with significant associations for the LP (*ABCG2*, *SH3BP5*, *KCNJ2*, *PICALM*) and PP phenotypes (*ABCG2*). For this cluster of genes, prominent peaks were observed in bands of the mid-infrared spectrum from ~ 1020 to 1180 cm^−1^, from ~ 1200 to 1470 cm^−1^, from ~ 2610 to 2840 cm^−1^ and from ~ 2870 to 2980 cm^−1^. The second cluster (Fig. 6b) includes genes with significant associations for the FP (*USP3*, *ELAPOR1*, *TBC1D22A*) and PP (*USP3*, *LMAN1*, *FA2H*, *TBC1D22A*, *STAT5A*) phenotypes, with multiple peaks observed across the mid-infrared spectrum, with the most prominent of these being in the ranges from ~ 910 to 1010 cm^−1^, from ~ 1070 to 1560 cm^−1^, from ~ 1700 to 2450 cm^−1^, from ~ 2630 to 2980 cm^−1^ and from ~ 3620 to 3680 cm^−1^. The third cluster (Fig. 6c) includes a number of genes with significant associations for the FP (*DGAT1*, *ABO*, *TGDS*, *GPAT4*, *MGST1*, *MROH1*) and PP (*DGAT1*, *MGST1*) phenotypes. For this cluster of genes, peaks were observed in many bands of the mid-infrared spectrum in common with peaks for *ABCG2* and *USP3* (Fig. 6a, b), including from ~ 910 to 1010 cm^−1^, from ~ 1130 to 1260 cm^−1^, from ~ 1450 to 1500 cm^−1^, from ~ 1700 to 2450 cm^−1^, and from 3620 to 3680 cm^−1^. Other notable peaks observed for this cluster were from ~ 1570 to 1700 cm^−1^, from ~ 2820 to 3150 cm^−1^, and from ~ 3460 to 3530 cm^−1^.

**Fig. 7:**
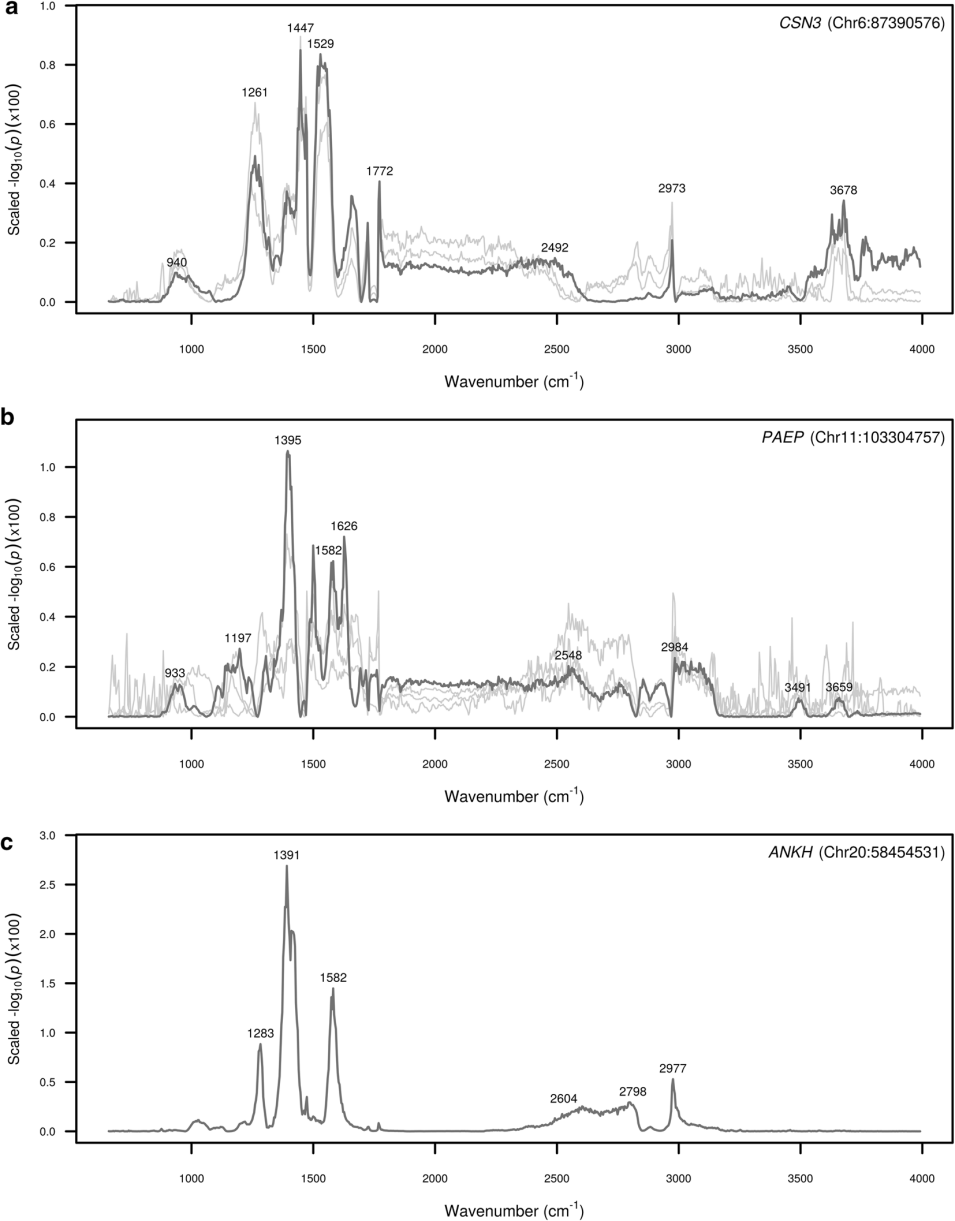
Significance profiles across the mid-infrared spectrum for tag variants of candidate genes within gene clusters. Y-axis values represent the strength of association signals as the −log_10_(*p*-value) of the effect, scaled to sum to unity across the mid-infrared spectral range. The significance profile for the most highly associated tag variant is shown in dark grey with the profiles for other genes within the cluster shown in light grey: **a** *CSN3* (Chr6:87390576; dark grey), *CSN1S1* (Chr6:87274397), *TAB2* (Chr9:87585031); **b** *PAEP* (Chr11:103304757; dark grey), *MPC1* (Chr9:102874726), *WDR97* (Chr14:1726650), *CCDC57* (Chr19:51303887); and **c** *ANKH* (Chr20:58454531)

Significance profiles for gene clusters represented by *CSN3*, *PAEP* and *ANKH* are shown in Fig. 7. The pattern of significance in the profiles represented by *CSN3* and *PAEP* (Fig. 7a and b) were similar, in that a large proportion of the signal was captured within a small part of the mid-infrared range; namely from ~ 1220 to 1780 cm^−1^ for the gene cluster represented by *CSN3*, and from ~ 1350 to ~ 1650 cm^−1^ for the gene cluster represented by *PAEP*. Although *ANKH* appeared to be an outlier in the clustering analysis (Fig. 5), a similar pattern was observed with most of the signal captured within three prominent peaks in the range from ~ 1260 to 1620 cm^−1^. Two of these peaks, centred at ~ 1391 cm^−1^ and 1582 cm^−1^ were in common with peaks observed for the *PAEP* profile. From the first cluster (Fig. 7a), *CSN3* was the only gene with a significant association for a predicted milk composition trait, namely PP. From the second cluster of genes (Fig. 7b), the *PAEP* and *CCDC57* genes had significant associations with the FP phenotype, whilst *ANKH* had a significant association with the LP phenotype (Fig. 7c).

## Discussion

### GWAS for FT-MIR wavenumbers

While there have been many GWAS for FT-MIR predicted milk composition traits, there are relatively few studies reporting GWAS results for individual FT-MIR wavenumber phenotypes. This is not withstanding the fact that individual wavenumbers exhibit additional genetic signal, compared to that observed in FT-MIR predictions of major milk composition traits [41, 42], and that direct analysis of the individual wavenumbers could provide additional granularity to establish causal links between the genome and underlying milk composition. Here, we present the results of GWAS that were conducted across individual FT-MIR wavenumber phenotypes, and the use of an iterative approach to help differentiate multiple, overlapping QTL. In total, wavenumber QTL were observed across 450 1-Mbp genomic regions, whereas predicted-trait QTL were observed across only 246 1-Mbp genomic regions. Notably, many of the observed wavenumber QTL were for wavenumbers within mid-infrared regions that were characterised by low signal-to-noise ratios. Typically, spectral data in these low signal-to-noise regions are discarded from analyses; however, these results indicate that wavenumbers in these regions are potentially informative. The signals that we observed in these noise regions were within several genes, with the highest frequency and strongest signals for variants in the *DGAT1* gene. This corroborates findings from previous studies which also observed significant associations between the *DGAT1* K232A polymorphism and wavenumbers in the regions from 1619 to 1674 cm^−1^ and from 3073 to 3667 cm^−1^ [32, 41].

### Multiple FT-MIR wavenumber QTL observed

In total, 31 wavenumber QTL were identified that we deemed to be ‘highly significant’ (see Methods for definition). Highly significant QTL were also observed for 12 of these same loci in at least one FT-MIR predicted milk composition trait, whereby the locus was in high LD (${R}^{2}$ > 0.9) with the same PIV. The loci for the three largest of these effects were in perfect LD with missense mutations in the *ABCG2*, *PAEP* and *DGAT1* genes, respectively, that have been proposed to have major impacts on milk composition [56, 59, 60]. Notably, the missense variant in the *ABCG2* gene identified here (rs43702337) is the same Y581S variant that was previously reported to be associated with milk yield and composition in Holstein cattle [59]. The role of the *ABCG2* mutation in milk composition regulation can be assumed to derive from osmotic impacts due to its function as an efflux transporter [36], although the gene has recently also been implicated in the modulation of mammary epithelial cell proliferation [61]. The *PAEP* gene encodes the major whey protein β-lactoglobulin. The variant rs109625649 reported here (V134A) is one of the variants that distinguishes the ‘A’ and ‘B’ haplotypes of β-lactoglobulin [62]. The *PAEP* gene also exhibited an eQTL that was significantly correlated with wavenumber 2548 cm^−1^, which is concordant with previous reports of *PAEP* promotor variants associated with milk composition [63]. The gene *DGAT1* encodes diacylglycerol O-acyltransferase 1, which catalyses the final step in triglyceride production and which, given the substantial quantities of fat secreted during milk production, makes *DGAT1* a well-demonstrated and straightforward candidate gene for this effect. The variant rs109234250 (K232A) reported here has been widely ascribed to the effects of the *DGAT1* gene on milk production, with a recent study showing that these effects may be due in part to an expression-based mechanism [64].

For the effects observed in the *ABCG2*, *PAEP* and *DGAT1* genes, the *p*-values for the most significant FT-MIR wavenumber were always more significant than the comparable values for any of the milk composition traits. For example, the *p*-value for the most significant wavenumber at the chr6:38027010 locus, the missense mutation in *ABCG2* highlighted above (Y581S, rs43702337) [59], was 7.3e-948, whereas the *p*-values for the same variant for LP and PP were 9.0e-717 and 6.4e-115, respectively. Similarly, the *p*-value for the most significant wavenumber at the chr11:103304757 locus, the V134A *PAEP* mutation (rs109625649) was 1.2e-134, whereas the *p*-value for the same variant for FP was 4.3e-46; and the *p*-value for the most significant wavenumber at the chr14:1802265 locus, represented by the K232A *DGAT1* mutation (rs109234250) [56] was 1.5e-2607, whereas the *p*-value for the same locus for PP was 1.2e-61.

Multiple protein-coding mutations could be attributed to loci with QTL in both wavenumber and milk composition traits, highlighting genes that appear to be novel to the present study (*TGDS* and *DNAJB13*), and genes previously reported in other studies of milk composition traits: *GBA* [37, 65], *MTX1* [66], *SLC50A1* [36, 67], *CSF2RB* [66, 68, 69], *NCF4* [66, 69], *TBC1D22A* [70], *MROH1* [71] and *MTARC1* [36]. A number of other QTL that were in strong LD with a PIV were observed in FT-MIR wavenumbers, but not in the FT-MIR predicted milk composition traits. This included QTL highlighting genes that have been previously reported in other studies of bovine milk composition: *FCGR2B* [72], *SCAMP3* [66], *THBS3* [66], *CSN1S1*, *CSN2* and *CSN3* [65, 71], *ABO* [73, 74], *CPSF1* [75, 76], *SPAG1* [67], *RNF214* [36], *KAT2A* [36], *STAT5A* [77–79] and *CCDC57* [40, 80]; and QTL highlighting genes that appear novel: *FCRLA*, *WDR97*, *KRT9*, *KRT16*, *KRT17*, *HID1*, *KCNK1* and *NXF1*.

Although many regions highlighted single mutations that could be considered excellent candidate mutations for a given QTL, other loci presented more complex regions with multiple competing candidates. In some cases, candidate genes at these loci have previously been proposed; however, it is possible that one or more novel genes may explain minor QTL that map to the same positions. For example, the chr3:15.4–15.6 Mbp region which includes the genes *FAM189B*, *GBA*, *HCN3*, *MTX1*, *SCAMP3*, *THBS3* and *SLC50A1*; the chr14:1.7–1.8 Mbp region, which includes the genes *WDR97*, *MROH1*, *CPSF1*, *SLC52A2* and the *DGAT1* K232A amino acid substitution; the chr19:42.4–42.5 Mbp region which includes the genes *KRT9*, *KRT42*, *KRT16* and *KRT17*; and the chr19:43.0–43.1 Mbp region which includes the genes *KAT2A*, *KCNH4* and *STAT5A*. These regions might represent multiple, linked QTL, or instances of single QTL where the LD structure and our relatively simple approach for identifying candidate genes was ineffective at differentiating them. Another possibility is that wavenumbers in these regions detect the presence of multiple chemically-similar compounds, with milk concentrations being influenced by different proteins, such as enzymes or transporters that are encoded by different genes.

### Co-localized eQTL suggest widespread regulatory impacts on FT-MIR wavenumbers

Of the 38 significant FT-MIR wavenumber QTL with co-localized eQTL, 18 also had co-localized eQTL that were observed for an FT-MIR predicted milk composition trait. In many cases, the tag variant for the wavenumber QTL was also the top variant for the co-localized eQTL. Genes corresponding to these effects have previously been published in other studies of bovine milk composition: *SH3BP5* [36], *SLC50A1* [36, 67], *USP3* [81, 82], *IVNS1ABP* [36], *KCJN2* [36], *ANKH* [36, 71], *GPAT4* [83, 84] and *PICALM* [36, 71]. Other cases for which the wavenumber QTL was in high LD (${R}^{2}$ > 0.9) with the top eQTL variant, highlighted genes previously published in other studies of bovine milk composition: *LRRC8C* [36], *CSF2RB* [66, 68, 69], *MGST1* [35, 70], *CSN3* [65, 71], *ABO* [73, 74], *GHDC* [36, 66] and *LMAN1* [70, 85]; and genes that appear to be novel to the present study: *KRTCAP2* and *FA2H*. Pearson correlations between log-scaled *p*-values for the trait and expression QTL for the latter two effects were 0.94 and 0.88, respectively, with both displaying very strong LD between the trait QTL and the most highly significant eQTL variant (${R}^{2}$ > 0.995).

Many wavenumber QTL with a co-localized eQTL also had a co-localized eQTL identified for a predicted milk composition trait. In these cases, a common pattern was observed whereby the wavenumber QTL had more highly significant *p*-values, compared to the *p*-values for the predicted trait. This was the case for *MGST1*, *ANKH*, *GPAT4* and *PICALM*. Notably, significant wavenumber QTL were detected for several additional milk proteins, with either highly-significant coding variants (*CSN1S1*, *CSN2*, *CSN3*) or a co-localized eQTL (*LTF*). To our surprise, only the *CSN3* eQTL was identified by analysis of the milk composition traits, with a *p*-value of 2.9e-45 for the PP phenotype, which was less significant than the *p*-value for the most significant wavenumber (*p*-value = 1.1e−97).

Other wavenumber QTL where a co-localized eQTL was identified within FT-MIR wavenumbers, but not the predicted milk composition traits, included effects that highlighted a number of genes that appear novel to the present study: *CLDN8*, *CSTB*, *TA**B2*, *LAPTM4A*, *CAPN5*, *PMP22*, *HID1* and *THRB*; and a number of genes previously reported as having an effect on bovine milk composition: *SLC37A1* [66, 86], *NCF4* [66, 69], *SLC34A2* [87], *TENT5A* [40], *RNF217* [67], *MPC1* [85], *XDH* [88, 89], *PAEP* [60], *DGAT1* [56], *RGL1* [90], *LTF* [91, 92] and *MUS81* [93]. These results underscore the gain in power that is available when using individual FT-MIR wavenumber phenotypes, compared to using predicted milk composition phenotypes which are linear functions of FT-MIR absorbance values.

### Candidate causative variants of note

Although we identified a large number of candidate causative variants for FT-MIR wavenumbers and predicted milk composition phenotypes, variants in perfect LD with a tag locus (${R}^{2}$ = 1) warrant further discussion. These associations presented missense variants for genes mentioned previously (*ABCG2*, *PAEP* and *DGAT1)*, in addition to other genes that have previously been linked to bovine milk composition phenotypes (*CSN2*, *CSN3*, *ABO*, *SPAG1* and *STAT5A*). Of these, the *ABO* exon 5 splice donor mutation (rs207688357; chr11:104242578C > G) is a particularly interesting and seemingly novel candidate causative variant identified through our GWAS of FT-MIR wavenumbers.

The rs207688357 variant was selected as the representative peak tag variant for 11 wavenumbers, with the most significant peak association observed for wavenumber 1462 cm^−1^. Visualisation of RNA-seq alignments confirmed that this variant disrupts splicing in carrier and homozygous animals (Fig. 4), where the mutation appears to activate two cryptic splice sites. The first and comparatively higher expressed form of these alternative transcripts is a − 8-bp frameshifted isoform predicted to lead to premature termination, while the lowly expressed in-frame form is predicted to introduce 11 new amino acids following the 78^th^ residue (due to a + 33 bp exon 5 extension). In humans, *ABO* has a widely recognised role as encoding the glycosyltransferases that catalyse the synthesis of the oligosaccharide ABO blood group antigens [94, 95]. Since both the alternatively spliced forms of bovine *ABO* generated by rs207688357 could be assumed to be non-functional (or at least dysfunctional for the minority in-frame isoform), this mutation would be akin to the human O blood group in homozygotes, where analogous human null alleles generate a non-functional enzyme [96]. These antigens are best known due to their expression on the surface of red blood cells, although they are also expressed on epithelial cells, as well as appearing as free oligosaccharides in milk [97]. This finding suggests a mechanism by which non- or partially functional bovine *ABO* alleles change carbohydrate structures in milk, therefore presenting differing FT-MIR signals detected by GWAS.

It should also be noted that although we are unaware of other studies proposing the rs207688357 (chr11:104242578) mutation as underlying such effects, other studies have reported genetic associations for bovine milk oligosaccharides for the broader *ABO* locus [73, 74]. One of these studies proposed an *ABO* p.Arg206Gln (R206Q; chr11:104232763; rs110960674) amino acid substitution present on the Illumina BovineHD chip as a potential causative mutation for this effect [74]. The other study reported associations with non-coding variants downstream of the *ABO* coding sequence (lead variant chr11:104229609; rs110534892), in this case using imputed sequence-based genotypes [72]. Both the p.Arg206Gln variant and the non-coding rs110534892 variant are also significant in our population, alongside the rs207688357 splice donor mutation, with peak association observed for the 1462 cm^−1^ wavenumber phenotype. These alternative candidates are less strongly associated than the rs207688357 splice donor mutation (*p*-value = 1.1e-23 and 1.8e-28, for the p.Arg206Gln and rs110534892 variants, respectively, compared to 5.5e-33). While these findings might suggest that these variants are simply linked to the functionally more compelling rs207688357 splice donor mutation, LD between the variants and the splice donor mutation is moderate to low (${R}^{2}$ = 0.486 and ${R}^{2}$ = 0.296 for the p.Arg206Gln and rs110534892 variants, respectively). Furthermore, when fitting the rs207688357 splice donor mutation as a covariate in the iterative association analysis of wavenumber 1462 cm^−1^, both variants retain residual signal (*p*-values of 4.2e-04 and 1.4e-07 for the p.Arg206Gln and rs110534892 variants, respectively), which suggests that all three variants might contribute to the oligosaccharide content of milk. In support of this concept, we also note that the non-coding rs110534892 variant proposed by Liu et al*.* [73] is in strong LD with the lead variant representing a strong *ABO* eQTL highlighted in our study (${R}^{2}$ = 0.944; Table 3). By contrast, the splice donor mutation is comparatively modestly associated with *ABO* expression at the whole transcript level (*p*-value = 9.1e−11 versus 5.9e−27), which suggests that multiple molecular mechanisms (missense, non-sense, and *cis*-regulatory effects) might contribute to oligosaccharide modulation at this locus.

### FT-MIR wavenumber association patterns for genes of interest

Although FT-MIR spectroscopy is a valuable tool for predicting a range of milk composition traits, there are limitations to the approach, i.e. it is often unable to detect molecules that are present in small quantities, and does not discriminate well between compounds that are chemically similar. Nevertheless, we have demonstrated that individual FT-MIR wavenumber phenotypes can provide valuable insights for establishing causal links between the genome and milk composition. Having observed patterns of association across multiple FT-MIR wavenumbers for individual loci (i.e. genome positions that appeared to highlight specific subsets of wavenumbers), our aim was to formally detect these patterns of association through cluster analysis. We hypothesised that the identified clusters could be rationalised based on shared biology or the physico-chemical properties of the encoded molecules—given that these signatures would presumably reflect common functions and structures in milk.

The cluster with the largest number of individual attributed loci included genes with prominent roles in the regulation of fat synthesis such as *DGAT1*, *GPAT4*, and *MGST1* (Fig. 5). These three loci have been implicated in previous studies of milk fat percentage and fatty acid synthesis [35, 56, 57, 70, 83, 84]. *DGAT1* and *GPAT4* encode acyltransferase enzymes that are responsible for mammary triglyceride synthesis, so it seems likely that the highlighted cluster reflects wavenumbers that are sensitive to changes in milk fat content. Notably, the pattern of the effects observed for *DGAT1* (Fig. 6a) was very similar to those reported previously [32, 43]. Highly significant effects were observed for the *DGAT1* K232A polymorphism in bands of the spectrum that could be attributed to a number of different chemical bond interactions including: phosphorus compounds (from ~ 910 to 1010 cm^−1^) [98], triglyceride ester C–O stretching (from ~ 1,130 to 1260 cm^−1^) [99, 100], C–H bending vibrations of –CH_2_ and –CH_3_ (from ~ 1450 to 1500 cm^−1^) [45, 98], C=O stretching in polypeptides within the amide I band of protein (from ~ 1600 to 1700 cm^−1^) [99], carboxylic acid and C = O rotation and stretching of ester groups of fat (from ~ 1700 to 1800 cm^−1^) [101], and acyl chain C–H stretching (from ~ 2820 to 3150 cm^−1^) [100].

The cluster that included the *ABCG2* Y581S polymorphism (Fig. 5) had highly significant association effects across numerous FT-MIR wavenumbers, with the largest effects concentrated within the regions from ~ 1020 to 1470 cm^−1^ and from ~ 2610 to 2980 cm^−1^ (Fig. 6b). Bands of the mid-infrared spectrum related to the largest effects for the *ABCG2* Y581S polymorphism were attributable to hydroxyl groups related to lactose (from ~ 1020 to 1180 cm^−1^) [98, 102], amide III and phosphate bands (from ~ 1200 to 1390 cm^−1^) [99, 103], C–H bending vibrations for CH_2_ and –CH_3_ (from ~ 1410 to 1470 cm^−1^) [98], overtones and bands of lactose (~ 2600 upwards) [104], and C–H stretching vibrations of CH_2_ and –CH_3_ (from ~ 2700 to 2980 cm^−1^) [98]. Many of the mid-infrared bands with significant effects were ascribed to chemical bond interactions related to lactose, which is unsurprising, given that *ABCG2* and many of the other genes classified in the same cluster (*SH3BP5*, *PMP22*, *KCNJ2*, and *PICALM*) have been previously associated with lactose phenotypes [36, 71]. Notably, the strongest association effects for the *ABCG2* Y581S polymorphism were in different bands of the mid-infrared spectrum to the *DGAT1* K232A polymorphism, assumedly reflecting the different roles that these two genes play in altering milk composition.

Three other notable gene clusters were those represented by the *CSN3*, *PAEP* and *ANKH* genes (Fig. 7), which had a large proportion of significant signal captured within a small part of the mid-infrared range: *CSN3* (from ~ 1220 to 1780 cm^−1^), *PAEP* (from ~ 1350 to 1650 cm^−1^) and *ANKH* (from ~ 1260 to 1620 cm^−1^). The *CSN3* gene encodes κ casein, one of the most abundantly expressed proteins in milk. Bound at the aqueous-hydrophobic interface of casein micelles, κ casein content influences the size of these structures, thereby affecting various coagulation and cheese-making properties [105, 106]. The missense mutation reported here at chr6:87390576 (rs43703015) has been associated with milk composition traits and differential expression in mammary tissue [107]. The largest effects for the *CSN3* locus were in spectral regions related to amide III and phosphate bands (from ~ 1220 to 1320 cm^−1^), C–H stretching vibrations of CH_2_ and –CH_3_ (from ~ 1370 to 1480 cm^−1^), and N–H bending and C–N stretching in the amide II band (from ~ 1490 to 1590 cm^−1^) [108]. Previous studies have reported association effects for *CSN3* in similar bands of the mid-infrared spectrum, with specific wavenumbers coinciding with highly significant association effects observed in our study [32, 42, 43]. The *ANKH* gene encodes a transmembrane protein involved in pyrophosphate transport regulation, and is associated with lactose concentrations in milk [36, 71]. Interestingly, *ANKH* and *PAEP* shared a prominent peak for adjacent wavenumbers, 1391 cm^−1^ and 1395 cm^−1^, respectively. These wavenumbers were in a region related to carboxylic acid C = O bond stretching [98]. Another peak in common between these genes was centred on the 1582 cm^−1^ wavenumber, also in a region related to carboxylic acid C=O bond stretching [98]. Association effects in similar bands of the mid-infrared spectrum for *PAEP* have been reported in previous studies [32, 42, 43]. Although *ANKH* and *PAEP* shared peaks in their significance profiles, it is notable that they also had exclusive peaks. For *ANKH*, a distinct peak was observed in a region related to amide III and phosphate bands (from ~ 1270 to 1290 cm^−1^) [99, 103], and for *PAEP* a distinct peak was observed in a region related to C–NH peptide bonds and N–H stretching and bending vibrations of NH_2_ (from ~ 1600 to 1640 cm^−1^) [98, 109], which shows that although commonalities exist, there are also differences in the roles that these genes play in altering milk composition.

### Limitations of the present study and future perspectives

In this study, we demonstrated that GWAS conducted on individual FT-MIR wavenumbers can improve power for identifying QTL and candidate causal variants, compared to GWAS conducted on FT-MIR predicted milk composition traits. Although many QTL were successfully identified, several refinements to our approach could be expected to enable the identification of additional QTL. The first of these relates to the approach used in adjusting phenotypes prior to conducting the GWAS. The repeated measures model that we used for adjusting phenotypes included a random effect to capture individual animal variation, but did not use pedigree information to account for covariance between individuals. This means that genetic trend may have been captured in herd by test day effects. A more optimal, but computationally more expensive approach, would have been to fit a repeatability model including the additive relationship matrix, thereby ensuring more accurate partitioning of fixed and random effects. To assess the potential impact of this on the final GWAS results in our study, we generated adjusted phenotypes for FP, LP and PP using a full animal model with an additive relationship matrix, and compared these to the adjusted phenotypes evaluated from the simplified repeated measures model we report. The correlations between the adjusted phenotypes from the two models were all high: 0.983, 0.994 and 0.987 for FP, LP and PP respectively. This implies that although the model that we used may be considered suboptimal, it is likely that the use of this model would have only a very minor impact on the final GWAS results.

Other potential refinements to our approach specifically relate to genomic information and our strategy for identifying QTL. First, our study relied on datasets that were mapped to the UMD3.1 genome, whereas a newer reference genome (ARS-UCD1.2) that has improved sequence continuity and per-base accuracy [110] is now available. Future use of that reference genome might yield additional QTL, as well as reveal additional candidate mutations given the improvements in accompanying transcript annotations. Second, our approach could be extended to account for non-additive QTL. Recently, we conducted non-additive association mapping of growth and development traits in cattle, which highlighted a number of major-effect mutations that had not been identified through application of standard additive models [93]. Although the low MAF variants identified in that study would require larger samples than those explored here, future analyses based on larger populations might be expected to identify similar non-additive effects for FT-MIR wavenumber and predicted milk composition traits. Third, a more sophisticated methodology could be used for the selection of representative variants in each QTL peak. In our approach, we have iteratively taken the top variant from each peak based on the *p*-value of the association effect, and fitted this as a covariate in subsequent rounds of GWAS. This approach does not take nonlinear interactions between variants into account, and can lead to the selection of multiple variants in high LD with a single QTL, if that QTL is not itself represented by a single biallelic variant. Alternatively, multiple QTL at a single locus might be best tagged by a single, non-causal variant that captures multiple signals. In both these instances, factors such as imputation or genotyping error may also further compound these issues. To address this, a modified approach could be adopted, whereby gene annotation information and other genomic and molecular data sources are used to assist with variant selection. Finally, although we tried to identify causal variants representing a variety of molecular mechanisms including coding variants (missense and nonsense) and regulatory effects (through integration of mammary eQTL data), these approaches are far from comprehensive, and will still miss many candidates. Improved variant prediction methods, and generation of other functional datasets (e.g. ChIP-seq) could be used to map additional molecular QTL, where integration of those data would enhance fine mapping and identification of candidate variants [19].

## Conclusions

We conducted a sequence-based GWAS on individual FT-MIR wavenumber phenotypes, and employed gene annotation and mammary tissue gene expression datasets to identify candidate causative genes and variants. Compared to GWAS on predicted milk composition traits, GWAS on individual FT-MIR wavenumbers resulted in stronger association effects, and improved power for identifying candidate causal variants. Although many of the genomic regions with significant associations that we identified in this work have previously been linked to milk composition traits, we report the discovery of several loci that have never previously been linked to milk phenotypes. Examining patterns of significance across wavenumbers in the mid-infrared range for loci of interest provided further insights into the relationships between specific genes and the underlying chemical structure of milk. Leveraging this information and incorporating the candidate causative mutations that we have identified into genomic prediction could result in improved selection of dairy cattle for the ever-growing range of traits of interest to the industry.

## Supplementary Information

**Additional file 1**:** Figure S1**. Sequence resolution effects for highly significant wavenumber QTL. Effects shown for 14 base GWAS wavenumber QTL in high LD (*R*^2^ > 0.9) with a putative impact variant. Putative impact variants are defined as a splice region variant, or a moderate or high impact variant according to the SnpEff classification. 1-Mbp regions centred on the wavenumber QTL are shown. The x-axis represents positions on the UMD 3.1 *Bos taurus* reference genome; the y-axis shows the strength of association signal, represented as the −log_10_(*p*-value) of the effect for each variant. Effects are coloured based on the predicted effect of the variant on genes, according to the SnpEff classification. The horizontal red line shows the Bonferroni significance threshold of −log_10_(6.2e-13).

**Additional file 2**: **Table S2**. Peak variants of 27 protein-sequence association effects classified as moderately significant for FT-MIR wavenumber phenotypes. Moderately significant effects are those for which the −log_﻿10_(*﻿p*-value) of the effect was greater than 1x the Bonferroni threshold of −log_10_(6.2e-13) and the correlation between the tag variant and the protein-sequence variant was higher than 0.9, but the effect did not meet the criteria of a highly significant effect (see Table 1). Effects where the locus has been identified as highly significant based on the LD with one or more other genes (and is also present in Table 1) are shaded yellow. No. of hits is the number of wavenumbers for which the variant was selected as the representative (most significant) tag variant for a peak. Iterations are defined relative to the base GWAS, with the base GWAS represented as iteration 0. L = Low impact splice region variant; M = Moderate impact missense variant; H = High impact splice donor. **Table S3**. Minor allele frequencies and allele effects for WGS tag variants with a highly significant protein-sequence association effect in at least one FT-MIR wavenumber. **Table S4**. Association statistic profiles for 31 highly significant protein-sequence effects identified in FT-MIR wavenumber phenotypes. For each protein-sequence mutation, the proportion of phenotypic and genetic variance that it accounts for is shown for each of the 895 FT-MIR wavenumbers and three FT-MIR predicted milk composition phenotypes. The genetic variance for each phenotype is the SNP-based estimate evaluated by the Bolt-LMM software. **Table S5**. Chronological profiles across iterations for 17 highly significant protein-sequence association effects observed in FT-MIR wavenumbers, where the association is observed after fitting the top chromosomal variant(s) in previous GWAS iterations and/or the base GWAS as covariates. Iterations are defined relative to the base GWAS, with the base GWAS represented as iteration 0. *﻿P*-values at previous iterations for the phenotype, and *p﻿﻿*-values for the corresponding top chromosomal SNP in that iteration are provided. **Table S6**. Peak variants of 14 protein-sequence association effects classified as moderately significant for FT-MIR predicted milk composition traits. Moderately significant effects are those where the −log_10﻿_(*﻿p*-value) of the effect was greater than 1x the Bonferroni threshold of −log_﻿10_(6.2e-13) and the correlation between the tag variant and the protein-sequence variant was higher than 0.9, but the effect did not meet the criteria of a highly significant effect (see Table 2). Effects where the locus has been identified as highly significant based on the LD with one or more other genes (and is also present in Table 2) are shaded yellow. Iterations are defined relative to the base GWAS, with the base GWAS represented as iteration 0. FP = Fat %; LP = Lactose %; PP = Protein %; L = Low impact splice region variant; M = Moderate impact missense variant; H = High impact splice donor. **Table S7**. Minor allele frequencies and allele effects for WGS tag variants with a highly significant protein-sequence association effect in at least one FT-MIR predicted milk composition trait. FP = Fat %; LP = Lactose %; PP = Protein %. **Table S8**. Chronological profiles across iterations for highly significant protein-sequence association effects observed in FT-MIR predicted milk composition traits, where the association is observed after fitting the top chromosomal variant(s) in previous GWAS iterations and/or the base GWAS as covariates. Iterations are defined relative to the base GWAS, with the base GWAS represented as iteration 0. *P*-values at previous iterations for the phenotype, and *﻿p*-values for the corresponding top chromosomal SNP in that iteration have been provided. FP = Fat %; LP = Lactose %; PP = Protein %.

**Additional file 3**: **Figure S9**. Sequence resolution effects for 19 base GWAS wavenumber QTL with a co-localized expression QTL. 1-Mbp regions centred on the wavenumber QTL are shown. The x-axis represents positions on the UMD 3.1 *Bos taurus* reference genome; the y-axis shows the strength of association signal, represented as the −log_10_(*p*-value) of the effect for each variant. Effects are coloured based on the predicted effect of the variant on genes, according to the SnpEff classification. The horizontal red line shows the Bonferroni significance threshold of −log_﻿10_(6.2e-13).

**Additional file 4**: **Table S10**. Minor allele frequencies and allele effects for WGS tag variants with a significant association effect in FT-MIR wavenumbers and a co-localized eQTL. **Table S11**. Association statistic profiles for 38 loci with a co-localized eQTL for at least one FT-MIR wavenumber phenotype. The proportion of phenotypic and genetic variance accounted for by each locus is shown for each of the 895 FT-MIR wavenumber and three FT-MIR predicted milk composition phenotypes. The genetic variance for each phenotype is the SNP-based estimate evaluated by the Bolt-LMM software. **Table S12**. Chronological profiles across iterations for 19 significant association effects with a co-localized eQTL observed in FT-MIR wavenumbers, where the association is observed after fitting the top chromosomal variant(s) in previous GWAS iterations and/or the base GWAS as covariates. Iterations are defined relative to the base GWAS, with the base GWAS represented as iteration 0. *P*-values at previous iterations for the phenotype, and *p*-values for the corresponding top chromosomal SNP in that iteration are provided. **Table S13**. Minor allele frequencies and allele effects for WGS tag variants with a significant association effect in at least one FT-MIR predicted milk composition trait and a co-localized eQTL. FP = Fat %; LP = Lactose %; PP = Protein %.** Table S14**. Chronological profiles across iterations for 12 significant association effects with a co-localized eQTL observed in FT-MIR predicted milk composition traits, where the association is observed after fitting the top chromosomal variant(s) in previous GWAS iterations and/or the base GWAS as covariates. Iterations are defined relative to the base GWAS, with the base GWAS represented as iteration 0. *﻿P*-values at previous iterations for the phenotype, and *p*-values for the corresponding top chromosomal SNP in that iteration have been provided. FP = Fat %; LP = Lactose %; PP = Protein %.

**Additional file 5**:** Figure S15**. Significance profiles of associations between FT-MIR wavenumbers and loci/genes in high LD with a putative impact variant (PIV), or in high LD with the top variant of a co-localized eQTL. A PIV is defined as a splice region variant, or moderate or high impact variant, according to the SnpEff classification. Significance is expressed as the –log_10﻿_(*p*-value) between each FT-MIR wavenumber and locus/gene of interest.

## Data Availability

Phenotypic data representing individual FT-MIR wavenumbers and FT-MIR predicted milk composition traits has been submitted to the Dryad Digital Repository (https://doi.org/10.5061/dryad.qrfj6q5dj) [111]. Genotypes for tag variants representing trait QTL have also been uploaded under the same Dryad submission ID. Relevant eQTL for genes with co-localized trait and expression QTL peaks are available through the Dryad database portal [112]. Whole-genome sequences used for imputation of the genotypes presented in this paper have been deposited in the SRA database [113]. Additional data is available on reasonable request with the permission of Livestock Improvement Corporation, contingent on the execution of an appropriate transfer agreement.
