# Supplementary material for: Sequence-based genome-wide association study of individual milk mid-infrared wavenumbers in mixed-breed dairy cattle
Source: Genet Sel Evol. 2021 Jul 20;53:62. doi: 10.1186/s12711-021-00648-9 (PMC8290608; doi:10.1186/s12711-021-00648-9)
Supplement: Supplementary file 1 — Additional file 1: Figure S1. Sequence resolution effects for highly significant wavenumber QTL. Effects shown for 14 base GWAS wavenumber QTL in high LD (R2 > 0.9) with a putative impact variant. Putative impact variants are defined as a splice region variant, or a moderate or high impact variant according to the SnpEff classification. 1-Mbp regions centred on the wavenumber QTL are shown. The x-axis represents positions on the UMD 3.1 Bos taurus reference genome; the y-axis shows the strength of association signal, represented as the −log10(p-value) of the effect for each variant. Effects are coloured based on the predicted effect of the variant on genes, according to the SnpEff classification. The horizontal red line shows the Bonferroni significance threshold of −log10(6.2e-13). [file 12711_2021_648_MOESM1_ESM.pdf]

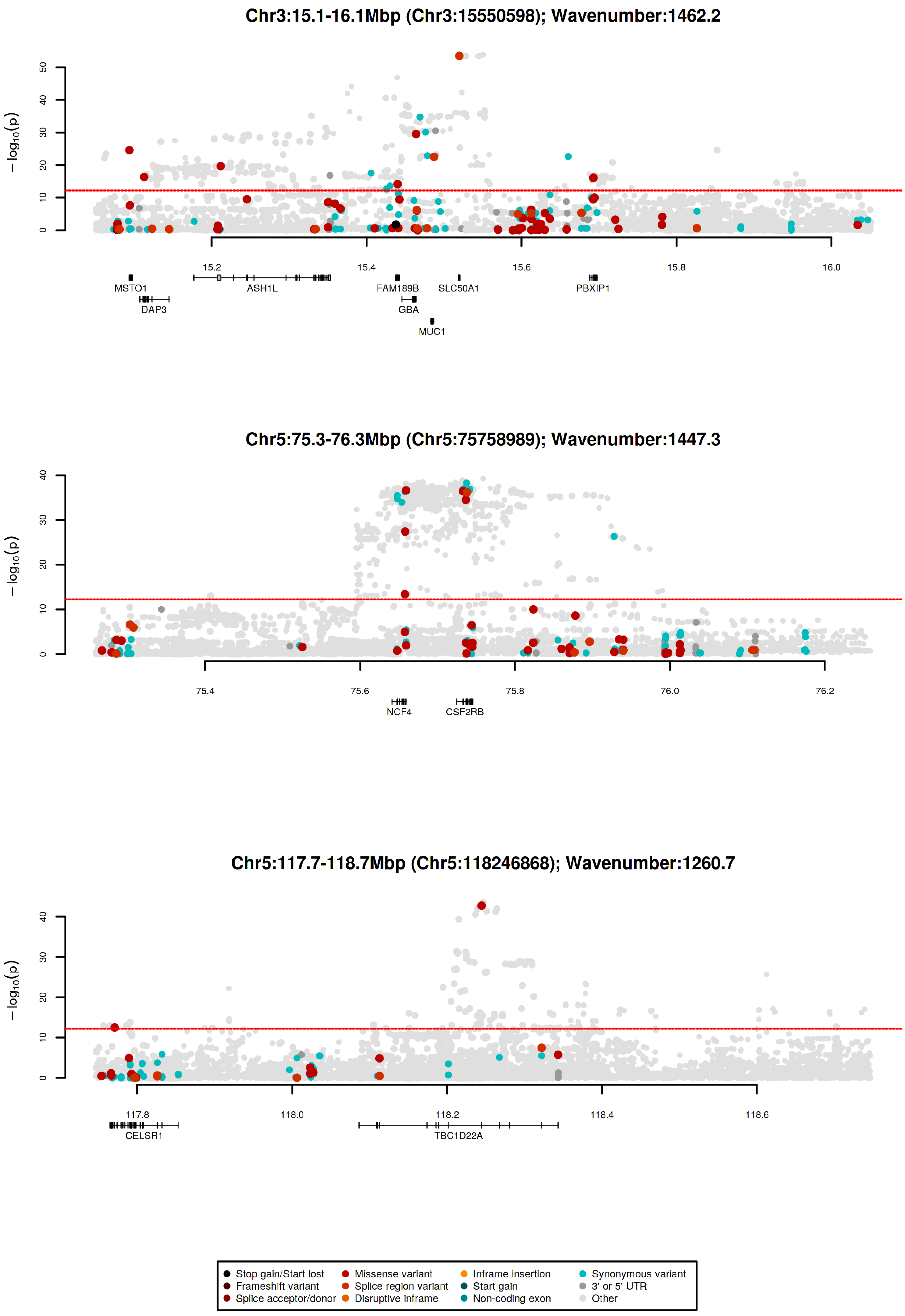

Chr3:15.1-16.1Mbp (Chr3:15550598); Wavenumber:1462.2
− log10(p)
0
10
20
30
40
50
15.2
15.4
15.6
15.8
16.0
MSTO1
DAP3
ASH1L
FAM189B
GBA
MUC1
SLC50A1
PBXIP1
Chr5:75.3-76.3Mbp (Chr5:75758989); Wavenumber:1447.3
− log10(p)
0
10
20
30
40
75.4
75.6
75.8
76.0
76.2
NCF4
CSF2RB
Chr5:117.7-118.7Mbp (Chr5:118246868); Wavenumber:1260.7
− log10(p)
0
10
20
30
40
117.8
118.0
118.2
118.4
118.6
CELSR1
TBC1D22A
Stop gain/Start lost
Frameshift variant
Splice acceptor/donor
Missense variant
Splice region variant
Disruptive inframe
Inframe insertion
Start gain
Non-coding exon
Synonymous variant
3' or 5' UTR
Other

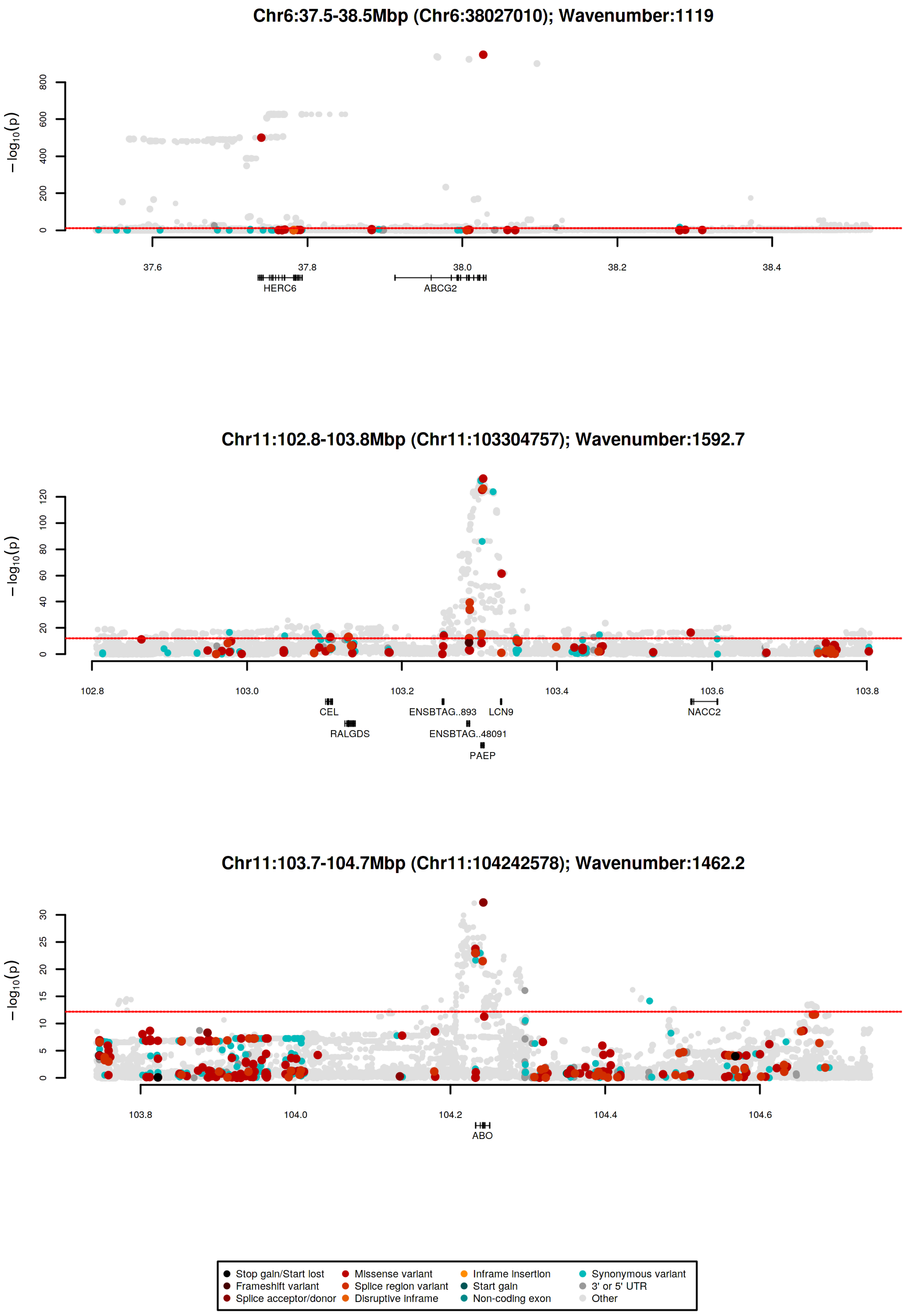
Chr6:37.5-38.5Mbp (Chr6:38027010); Wavenumber:1119
−log10(p)
0
200
400
600
800
37.6
37.8
38.0
38.2
38.4
HERC6
ABCG2
Chr11:102.8-103.8Mbp (Chr11:103304757); Wavenumber:1592.7
−log10(p)
0
20
40
60
80
100
120
102.8
103.0
103.2
103.4
103.6
103.8
CEL
RALGDS
ENSBTAG..893
ENSBTAG..48091
PAEP
LCN9
NACC2
Chr11:103.7-104.7Mbp (Chr11:104242578); Wavenumber:1462.2
−log10(p)
0
5
10
15
20
25
30
103.8
104.0
104.2
104.4
104.6
ABO
Stop gain/Start lost
Frameshift variant
Splice acceptor/donor
Missense variant
Splice region variant
Disruptive inframe
Inframe insertion
Start gain
Non-coding exon
Synonymous variant
3' or 5' UTR
Other

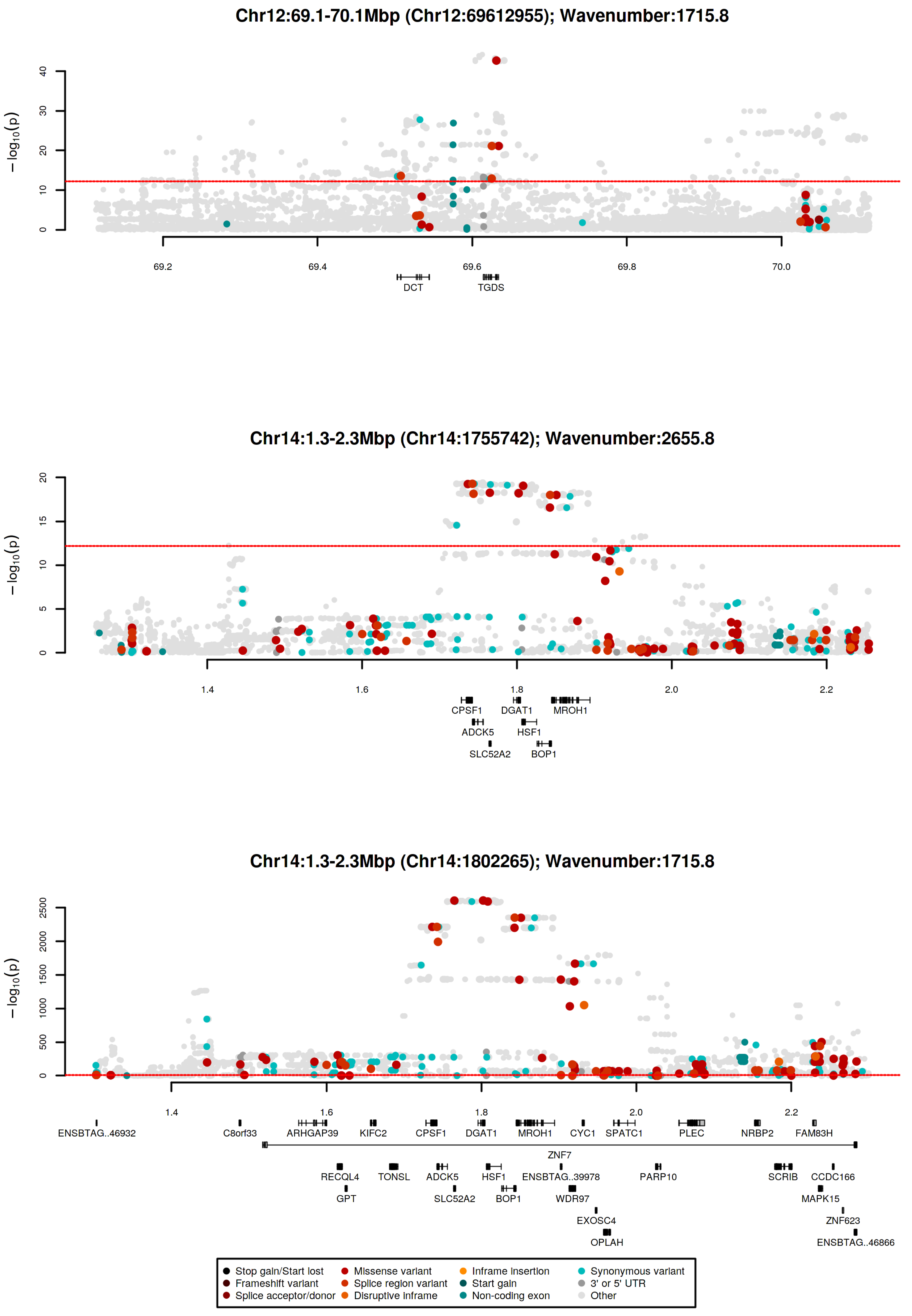

Chr12:69.1-70.1Mbp (Chr12:69612955); Wavenumber:1715.8
− log10(p)
0
10
20
30
40
69.2
69.4
69.6
69.8
70.0
DCT
TGDS
Chr14:1.3-2.3Mbp (Chr14:1755742); Wavenumber:2655.8
− log10(p)
0
5
10
15
20
1.4
1.6
1.8
2.0
2.2
CPSF1
DGAT1
MROH1
ADCK5
HSF1
SLC52A2
BOP1
Chr14:1.3-2.3Mbp (Chr14:1802265); Wavenumber:1715.8
− log10(p)
0
500
1000
1500
2000
2500
1.4
1.6
1.8
2.0
2.2
ENSBTAG..46932
C8orf33
ARHGAP39
KIFC2
CPSF1
DGAT1
MROH1
CYC1
SPATC1
PLEC
NRBP2
FAM83H
ZNF7
RECQL4
TONSL
ADCK5
HSF1
ENSBTAG..39978
PARP10
SCRIB
CCDC166
GPT
SLC52A2
BOP1
WDR97
MAPK15
EXOSC4
ZNF623
OPLAH
ENSBTAG..46866
Stop gain/Start lost
Frameshift variant
Splice acceptor/donor
Missense variant
Splice region variant
Disruptive inframe
Inframe insertion
Start gain
Non-coding exon
Synonymous variant
3' or 5' UTR
Other

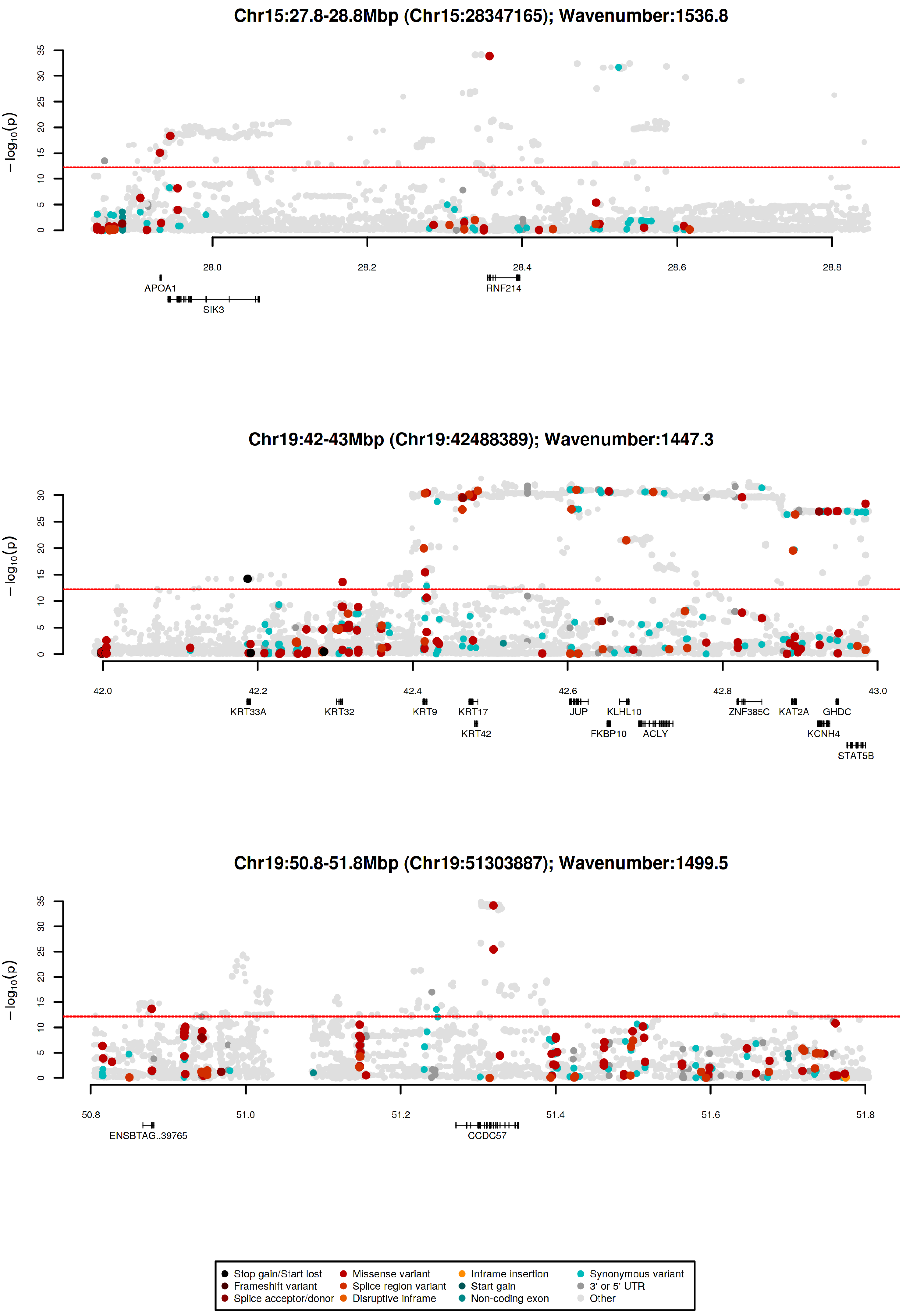
Chr15:27.8-28.8Mbp (Chr15:28347165); Wavenumber:1536.8
−log10(p)
0
5
10
15
20
25
30
35
28.0
28.2
28.4
28.6
28.8
APOA1
SIK3
RNF214
Chr19:42-43Mbp (Chr19:42488389); Wavenumber:1447.3
−log10(p)
0
5
10
15
20
25
30
42.0
42.2
42.4
42.6
42.8
43.0
KRT33A
KRT32
KRT9
KRT17
KRT42
JUP
KLHL10
FKBP10
ACLY
ZNF385C
KAT2A
GHDC
KCNH4
STAT5B
Chr19:50.8-51.8Mbp (Chr19:51303887); Wavenumber:1499.5
−log10(p)
0
5
10
15
20
25
30
35
50.8
51.0
51.2
51.4
51.6
51.8
ENSBTAG..39765
CCDC57
Stop gain/Start lost
Frameshift variant
Splice acceptor/donor
Missense variant
Splice region variant
Disruptive inframe
Inframe insertion
Start gain
Non-coding exon
Synonymous variant
3' or 5' UTR
Other

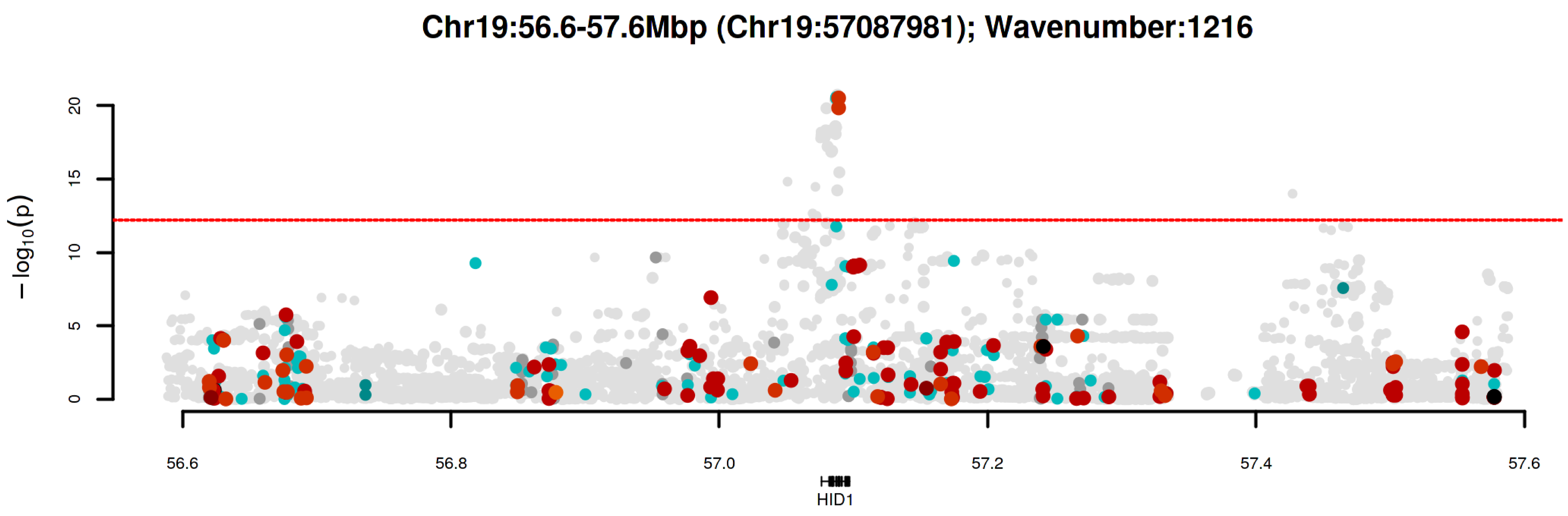
Chr19:56.6-57.6Mbp (Chr19:57087981); Wavenumber:1216
− log10(p)
0
5
10
15
20
56.6
56.8
57.0
57.2
57.4
57.6
HID1

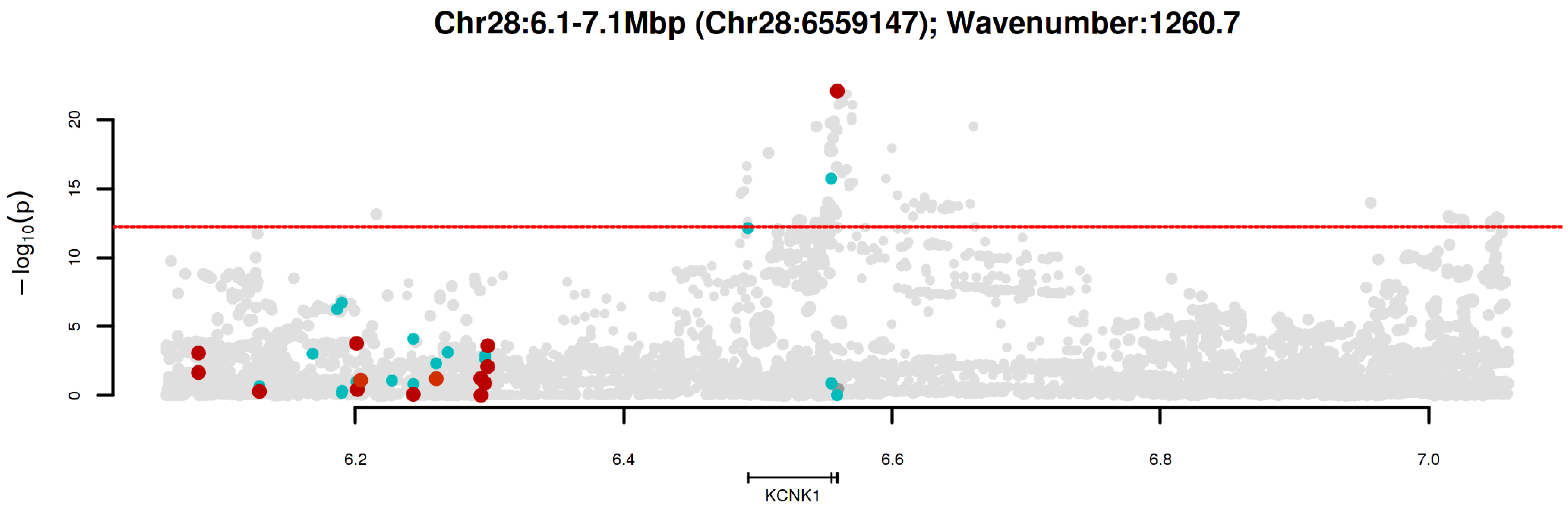
Chr28:6.1-7.1Mbp (Chr28:6559147); Wavenumber:1260.7
− log10(p)
0
5
10
15
20
6.2
6.4
6.6
6.8
7.0
KCNK1

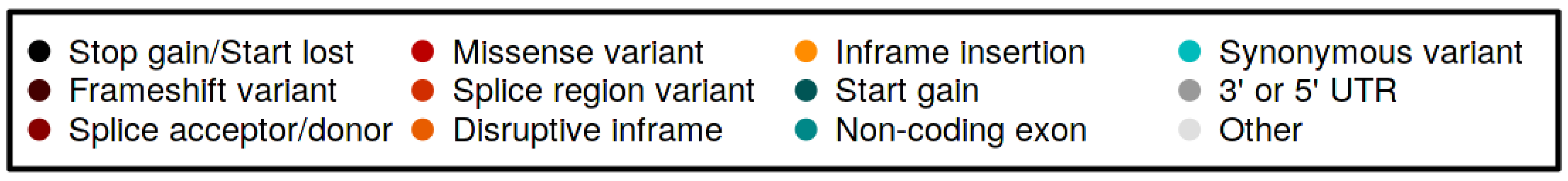
Stop gain/Start lost
Frameshift variant
Splice acceptor/donor
Missense variant
Splice region variant
Disruptive inframe
Inframe insertion
Start gain
Non-coding exon
Synonymous variant
3' or 5' UTR
Other
