# Supplementary material for: Sequence-based genome-wide association study of individual milk mid-infrared wavenumbers in mixed-breed dairy cattle
Source: Genet Sel Evol. 2021 Jul 20;53:62. doi: 10.1186/s12711-021-00648-9 (PMC8290608; doi:10.1186/s12711-021-00648-9)
Supplement: Supplementary file 3 — Additional file 3: Figure S9. Sequence resolution effects for 19 base GWAS wavenumber QTL with a co-localized expression QTL. 1-Mbp regions centred on the wavenumber QTL are shown. The x-axis represents positions on the UMD 3.1 Bos taurus reference genome; the y-axis shows the strength of association signal, represented as the −log10(p-value) of the effect for each variant. Effects are coloured based on the predicted effect of the variant on genes, according to the SnpEff classification. The horizontal red line shows the Bonferroni significance threshold of −log﻿10(6.2e-13). [file 12711_2021_648_MOESM3_ESM.pdf]

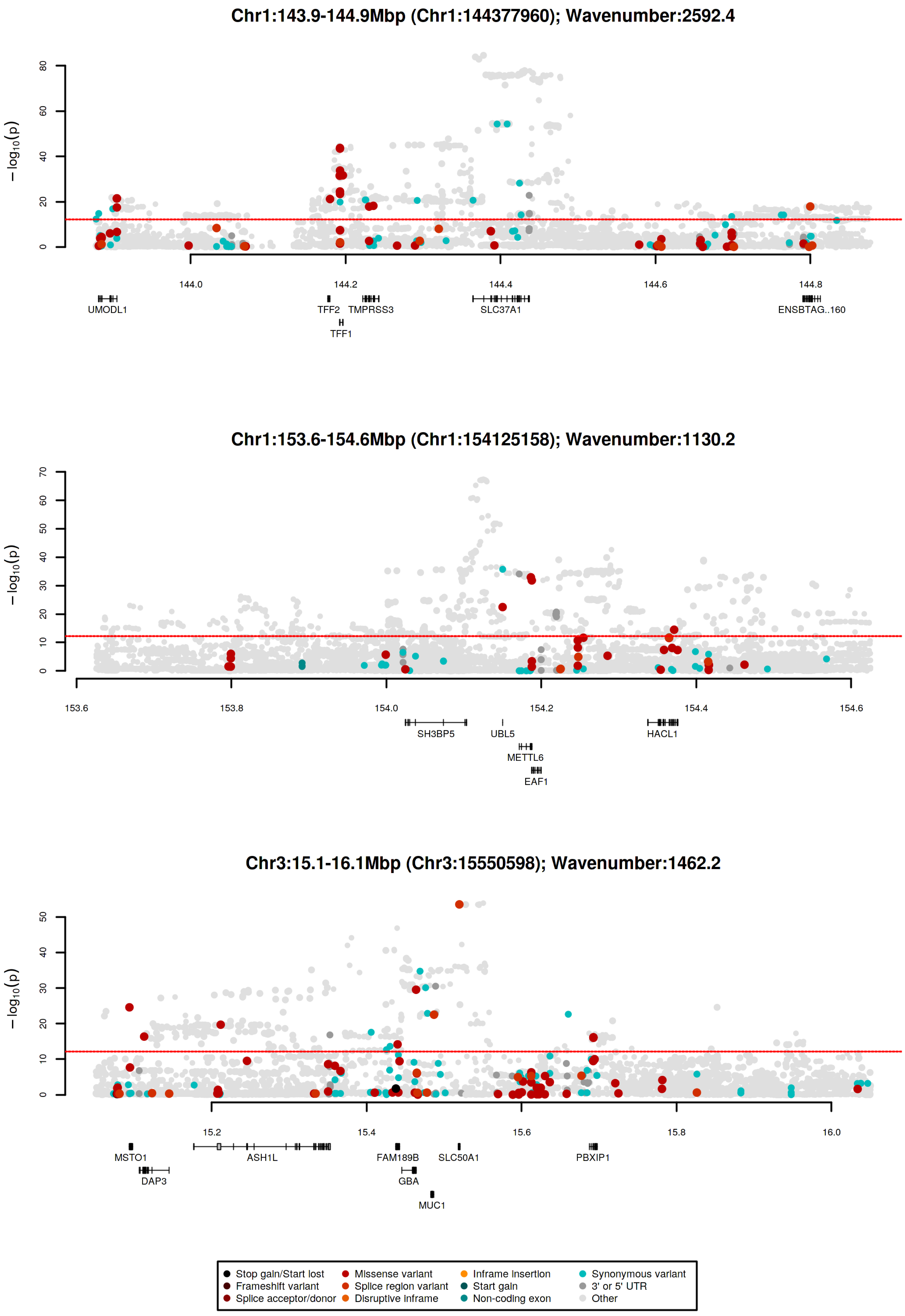
Chr1:143.9-144.9Mbp (Chr1:144377960); Wavenumber:2592.4
$-\log_{10}(p)$
0
20
40
60
80
144.0
144.2
144.4
144.6
144.8
UMODL1
TFF2
TMPRSS3
TFF1
SLC37A1
ENSBTAG..160
Chr1:153.6-154.6Mbp (Chr1:154125158); Wavenumber:1130.2
$-\log_{10}(p)$
0
10
20
30
40
50
60
70
153.6
153.8
154.0
154.2
154.4
154.6
SH3BP5
UBL5
METTL6
EAF1
HACL1
Chr3:15.1-16.1Mbp (Chr3:15550598); Wavenumber:1462.2
$-\log_{10}(p)$
0
10
20
30
40
50
15.2
15.4
15.6
15.8
16.0
MSTO1
DAP3
ASH1L
FAM189B
GBA
MUC1
SLC50A1
PBXIP1
Stop gain/Start lost
Frameshift variant
Splice acceptor/donor
Missense variant
Splice region variant
Disruptive inframe
Inframe insertion
Start gain
Non-coding exon
Synonymous variant
3' or 5' UTR
Other

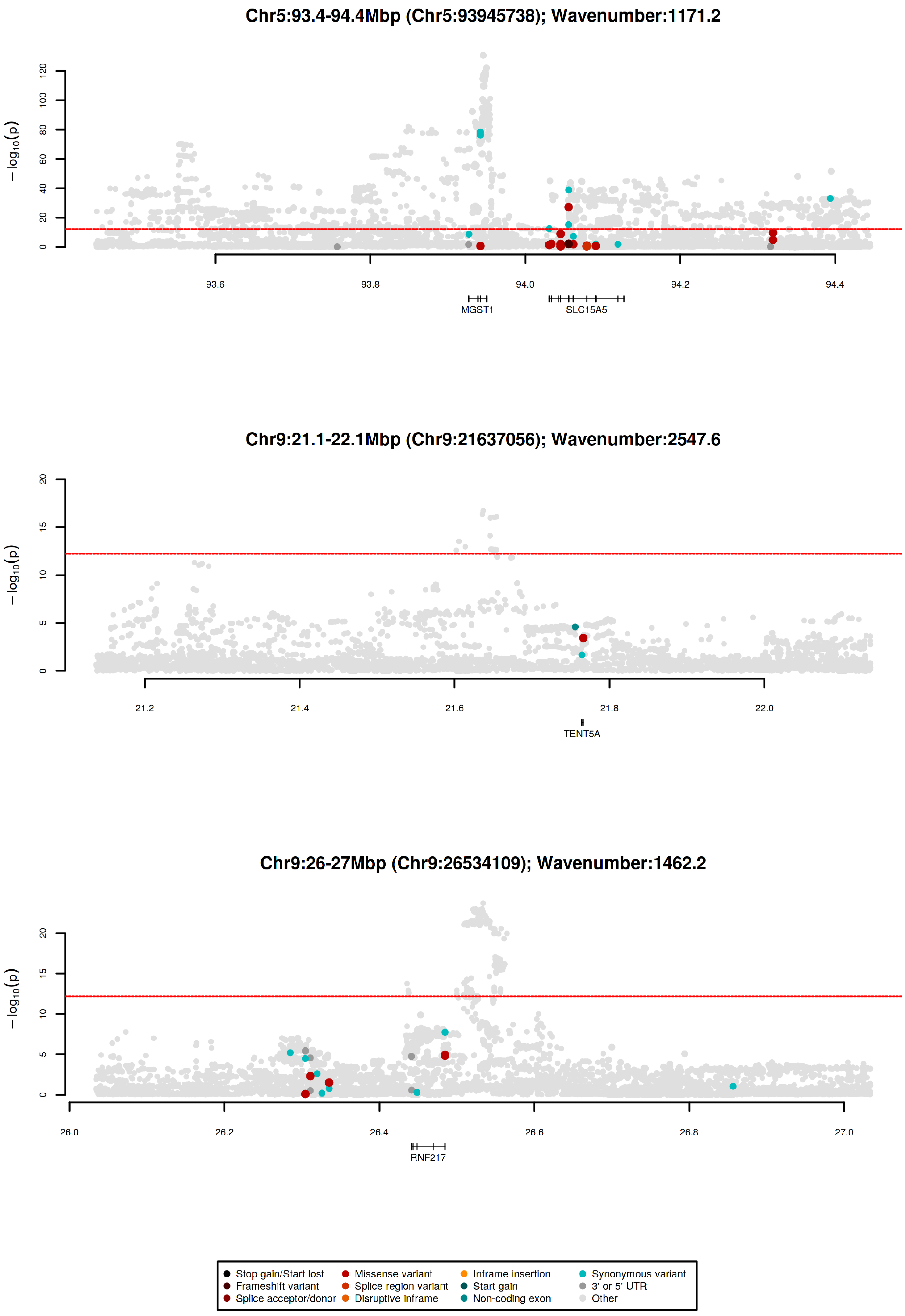

Chr5:93.4-94.4Mbp (Chr5:93945738); Wavenumber:1171.2
−log10(p)
0
20
40
60
80
100
120
93.6
93.8
94.0
94.2
94.4
MGST1
SLC15A5
Chr9:21.1-22.1Mbp (Chr9:21637056); Wavenumber:2547.6
−log10(p)
0
5
10
15
20
21.2
21.4
21.6
21.8
22.0
TENT5A
Chr9:26-27Mbp (Chr9:26534109); Wavenumber:1462.2
−log10(p)
0
5
10
15
20
26.0
26.2
26.4
26.6
26.8
27.0
RNF217
Stop gain/Start lost
Frameshift variant
Splice acceptor/donor
Missense variant
Splice region variant
Disruptive inframe
Inframe insertion
Start gain
Non-coding exon
Synonymous variant
3' or 5' UTR
Other

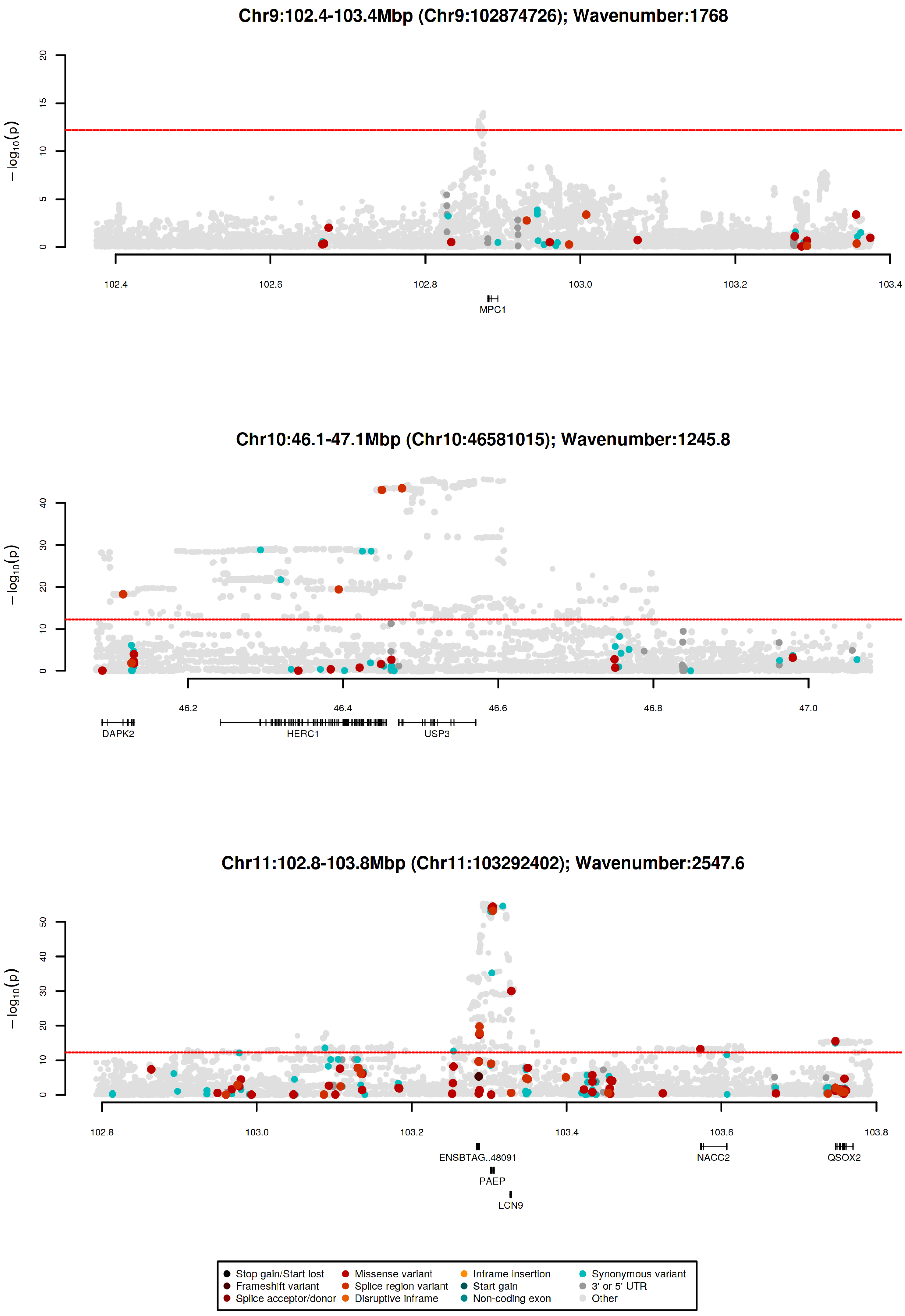
Chr9:102.4-103.4Mbp (Chr9:102874726); Wavenumber:1768
$-\log_{10}(p)$
0
5
10
15
20
102.4
102.6
102.8
103.0
103.2
103.4
MPC1
Chr10:46.1-47.1Mbp (Chr10:46581015); Wavenumber:1245.8
$-\log_{10}(p)$
0
10
20
30
40
46.2
46.4
46.6
46.8
47.0
DAPK2
HERC1
USP3
Chr11:102.8-103.8Mbp (Chr11:103292402); Wavenumber:2547.6
$-\log_{10}(p)$
0
10
20
30
40
50
102.8
103.0
103.2
103.4
103.6
103.8
ENSBTAG..48091
PAEP
LCN9
NACC2
QSOX2
Stop gain/Start lost
Frameshift variant
Splice acceptor/donor
Missense variant
Splice region variant
Disruptive inframe
Inframe insertion
Start gain
Non-coding exon
Synonymous variant
3' or 5' UTR
Other

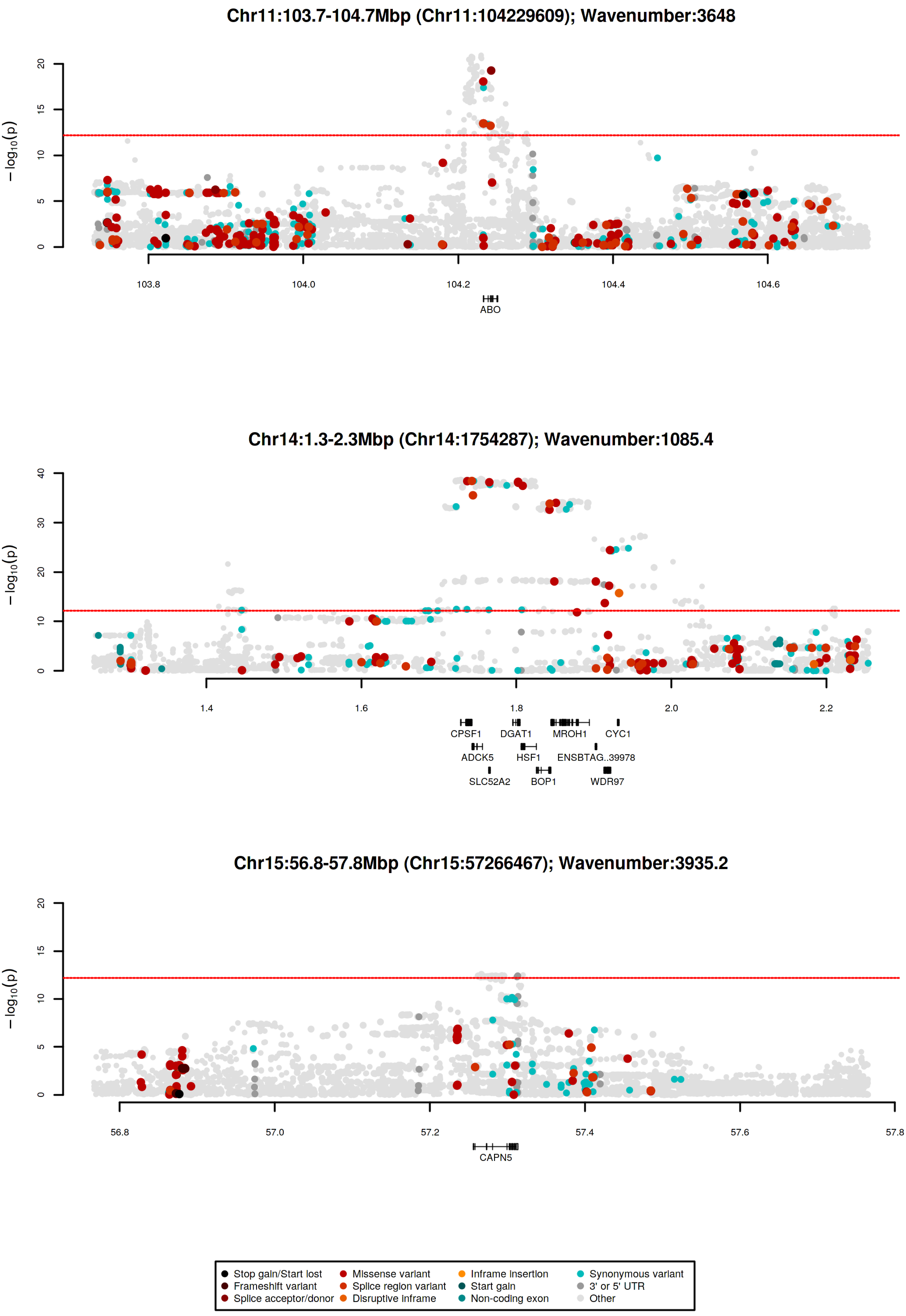

Chr11:103.7-104.7Mbp (Chr11:104229609); Wavenumber:3648
−log10(p)
0
5
10
15
20
103.8
104.0
104.2
104.4
104.6
ABO
Chr14:1.3-2.3Mbp (Chr14:1754287); Wavenumber:1085.4
−log10(p)
0
10
20
30
40
1.4
1.6
1.8
2.0
2.2
CPSF1
DGAT1
MROH1
CYC1
ADCK5
HSF1
ENSBTAG..39978
SLC52A2
BOP1
WDR97
Chr15:56.8-57.8Mbp (Chr15:57266467); Wavenumber:3935.2
−log10(p)
0
5
10
15
20
56.8
57.0
57.2
57.4
57.6
57.8
CAPN5
Stop gain/Start lost
Frameshift variant
Splice acceptor/donor
Missense variant
Splice region variant
Disruptive inframe
Inframe insertion
Start gain
Non-coding exon
Synonymous variant
3' or 5' UTR
Other

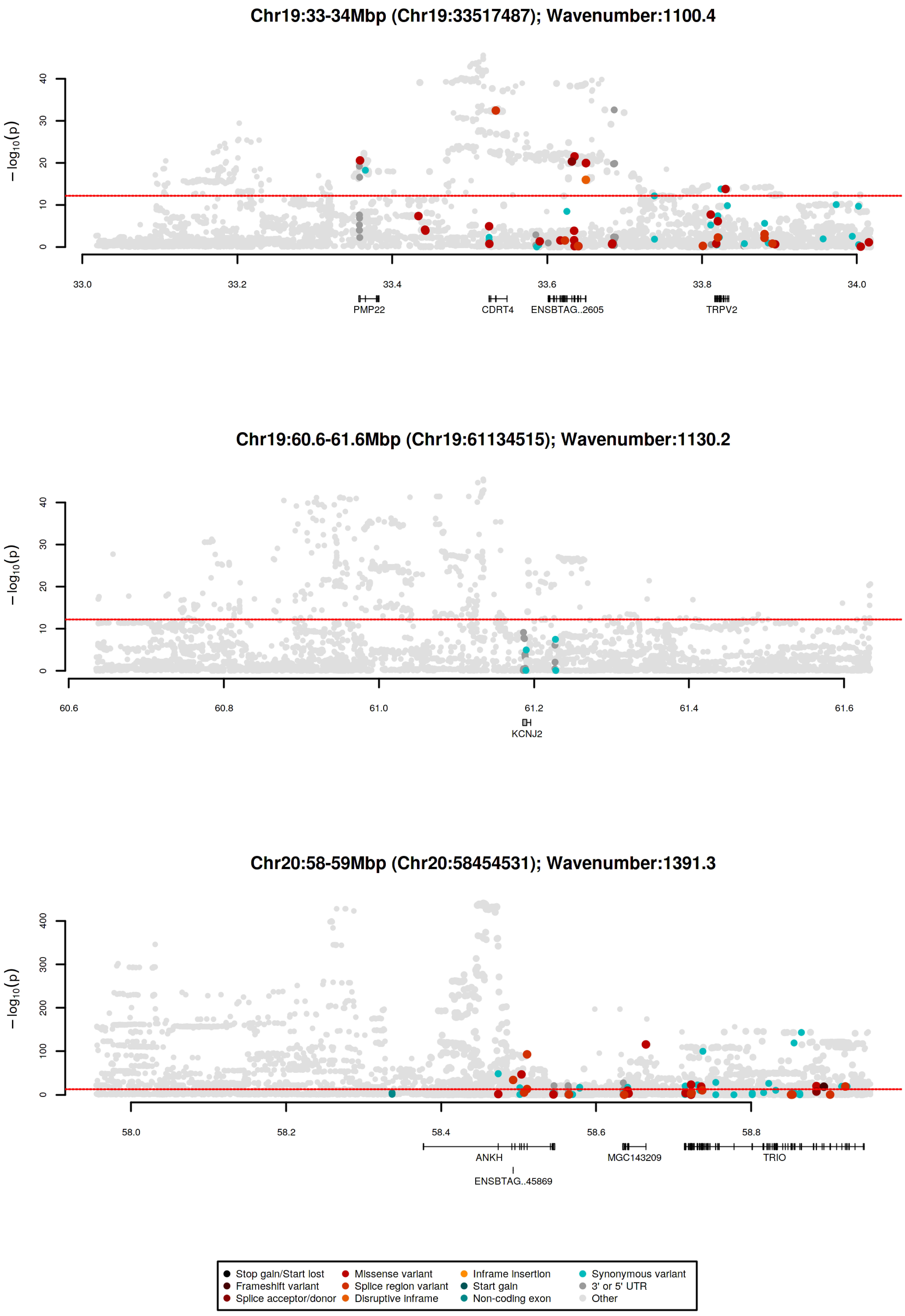
Chr19:33-34Mbp (Chr19:33517487); Wavenumber:1100.4
−log10(p)
0
10
20
30
40
33.0
33.2
33.4
33.6
33.8
34.0
PMP22
CDRT4
ENSBTAG..2605
TRPV2
Chr19:60.6-61.6Mbp (Chr19:61134515); Wavenumber:1130.2
−log10(p)
0
10
20
30
40
60.6
60.8
61.0
61.2
61.4
61.6
KCNJ2
Chr20:58-59Mbp (Chr20:58454531); Wavenumber:1391.3
−log10(p)
0
100
200
300
400
58.0
58.2
58.4
58.6
58.8
ANKH
ENSBTAG..45869
MGC143209
TRIO
Stop gain/Start lost
Frameshift variant
Splice acceptor/donor
Missense variant
Splice region variant
Disruptive inframe
Inframe insertion
Start gain
Non-coding exon
Synonymous variant
3' or 5' UTR
Other

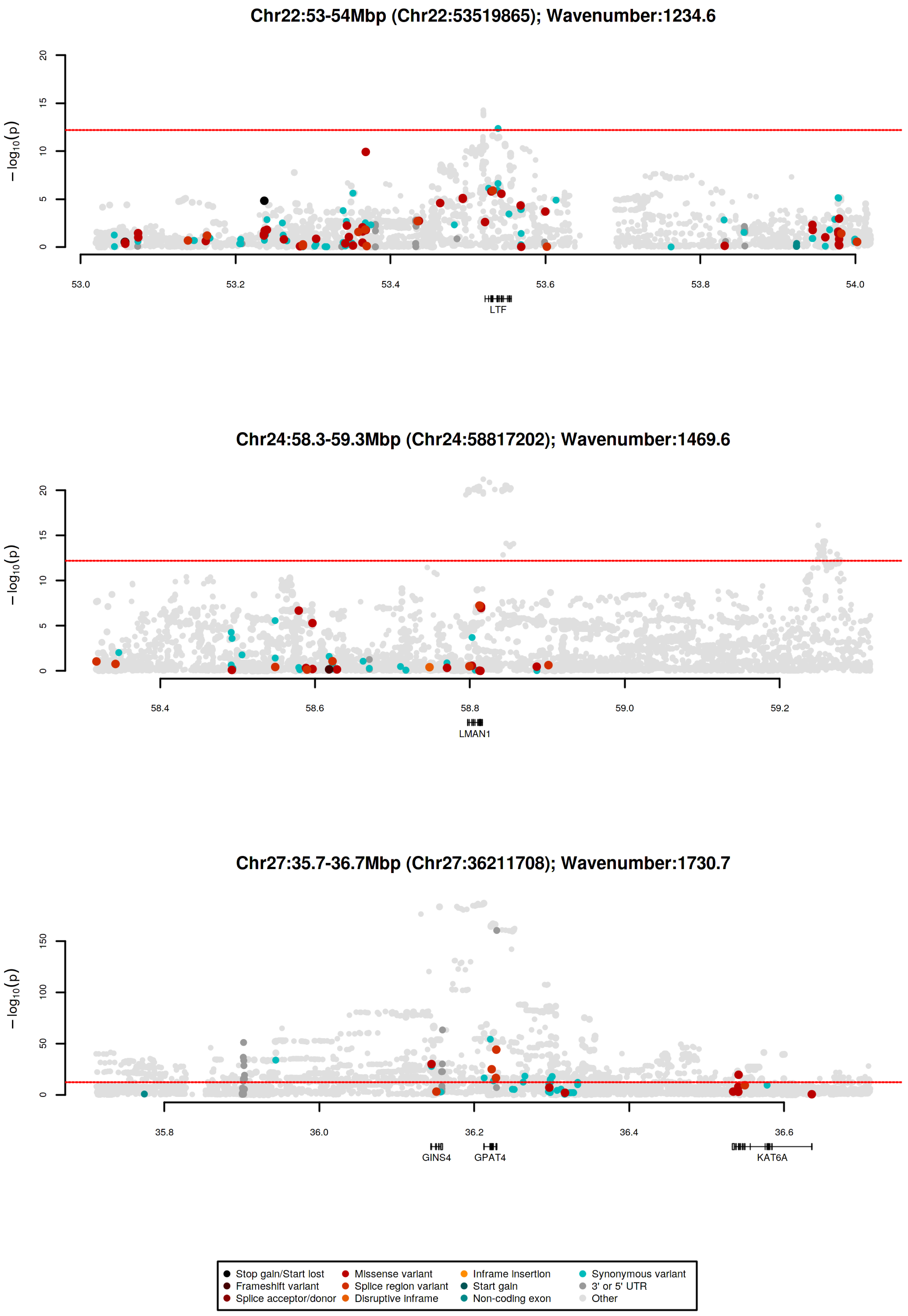

Chr22:53-54Mbp (Chr22:53519865); Wavenumber:1234.6
− log10(p)
0
5
10
15
20
53.0
53.2
53.4
53.6
53.8
54.0
LTF
Chr24:58.3-59.3Mbp (Chr24:58817202); Wavenumber:1469.6
− log10(p)
0
5
10
15
20
58.4
58.6
58.8
59.0
59.2
LMAN1
Chr27:35.7-36.7Mbp (Chr27:36211708); Wavenumber:1730.7
− log10(p)
0
50
100
150
35.8
36.0
36.2
36.4
36.6
GINS4
GPAT4
KAT6A
Stop gain/Start lost
Frameshift variant
Splice acceptor/donor
Missense variant
Splice region variant
Disruptive inframe
Inframe insertion
Start gain
Non-coding exon
Synonymous variant
3' or 5' UTR
Other

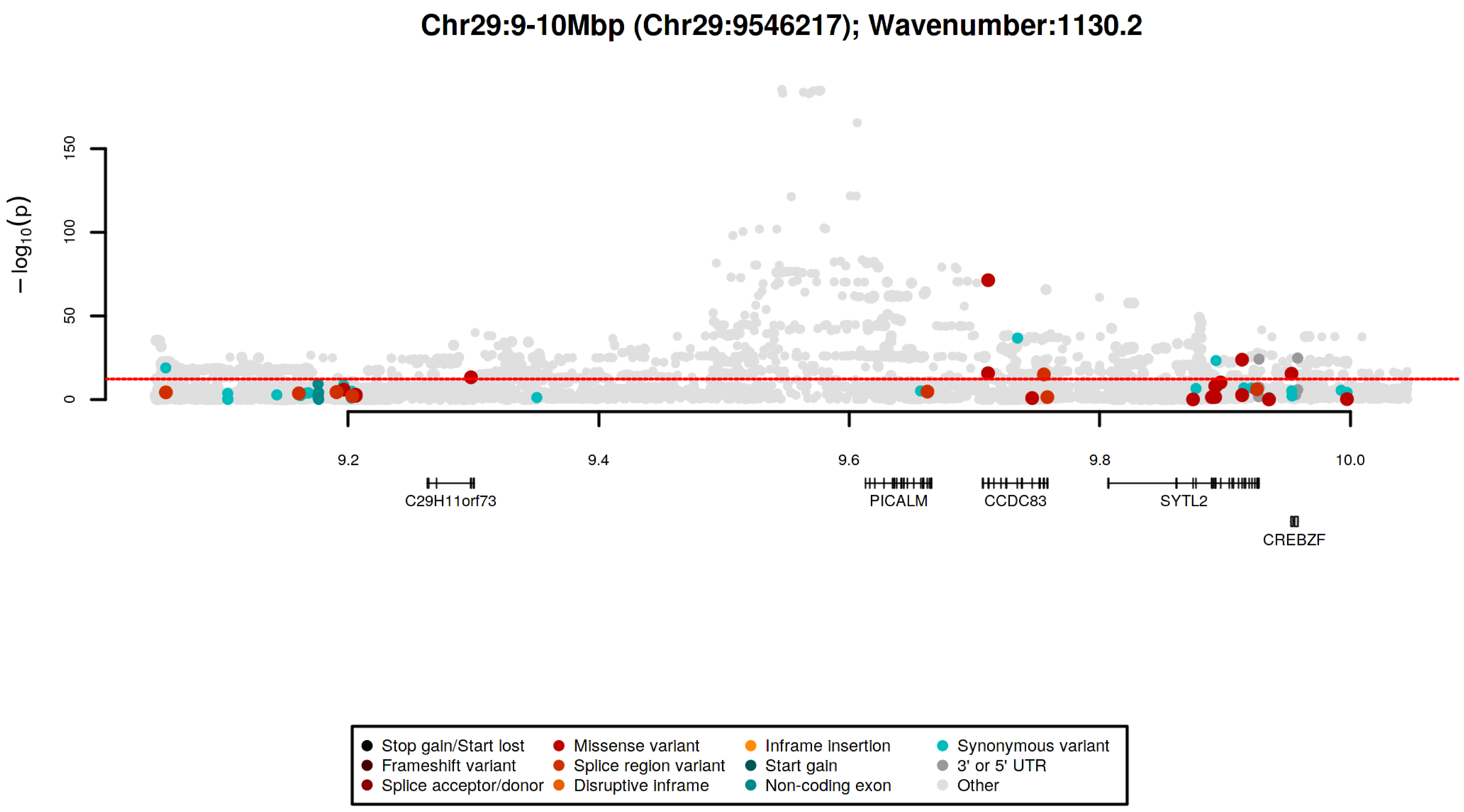

Chr29:9-10Mbp (Chr29:9546217); Wavenumber:1130.2
−log10(p)
0
50
100
150
9.2
9.4
9.6
9.8
10.0
C29H11orf73
PICALM
CCDC83
SYTL2
CREBZF
Stop gain/Start lost
Frameshift variant
Splice acceptor/donor
Missense variant
Splice region variant
Disruptive inframe
Inframe insertion
Start gain
Non-coding exon
Synonymous variant
3' or 5' UTR
Other
