# Supplementary material for: Sequence-based genome-wide association study of individual milk mid-infrared wavenumbers in mixed-breed dairy cattle
Source: Genet Sel Evol. 2021 Jul 20;53:62. doi: 10.1186/s12711-021-00648-9 (PMC8290608; doi:10.1186/s12711-021-00648-9)
Supplement: Supplementary file 5 — Additional file 5: Figure S15. Significance profiles of associations between FT-MIR wavenumbers and loci/genes in high LD with a putative impact variant (PIV), or in high LD with the top variant of a co-localized eQTL. A PIV is defined as a splice region variant, or moderate or high impact variant, according to the SnpEff classification. Significance is expressed as the –log10﻿(p-value) between each FT-MIR wavenumber and locus/gene of interest. [file 12711_2021_648_MOESM5_ESM.pdf]

CLDN8 (Chr1:5120248)

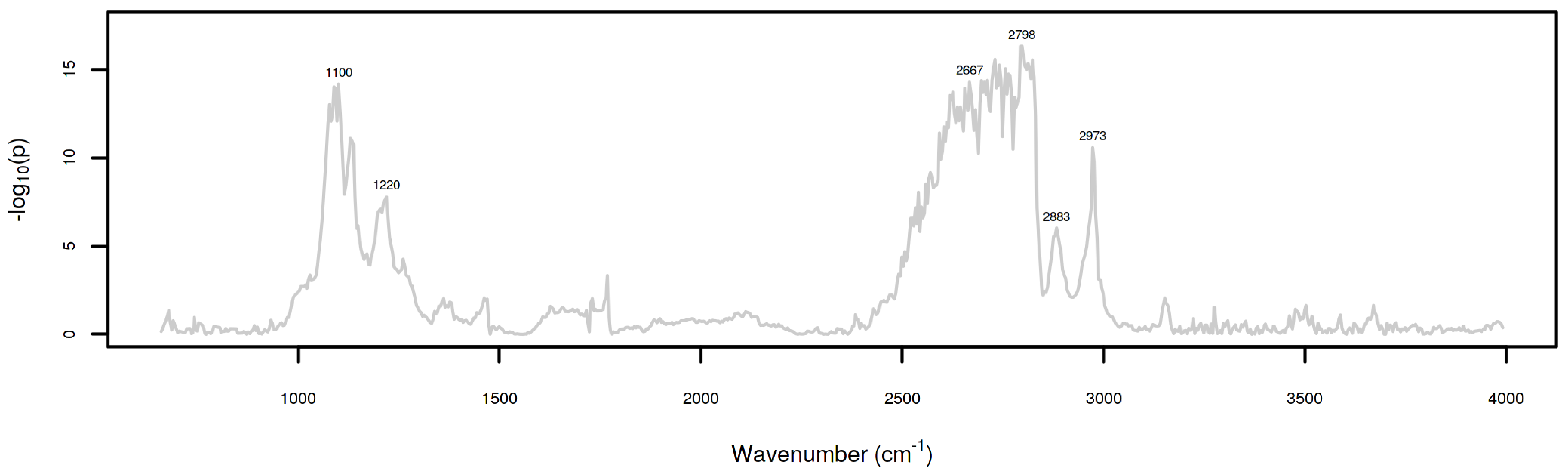

SLC37A1 (Chr1:144377960)

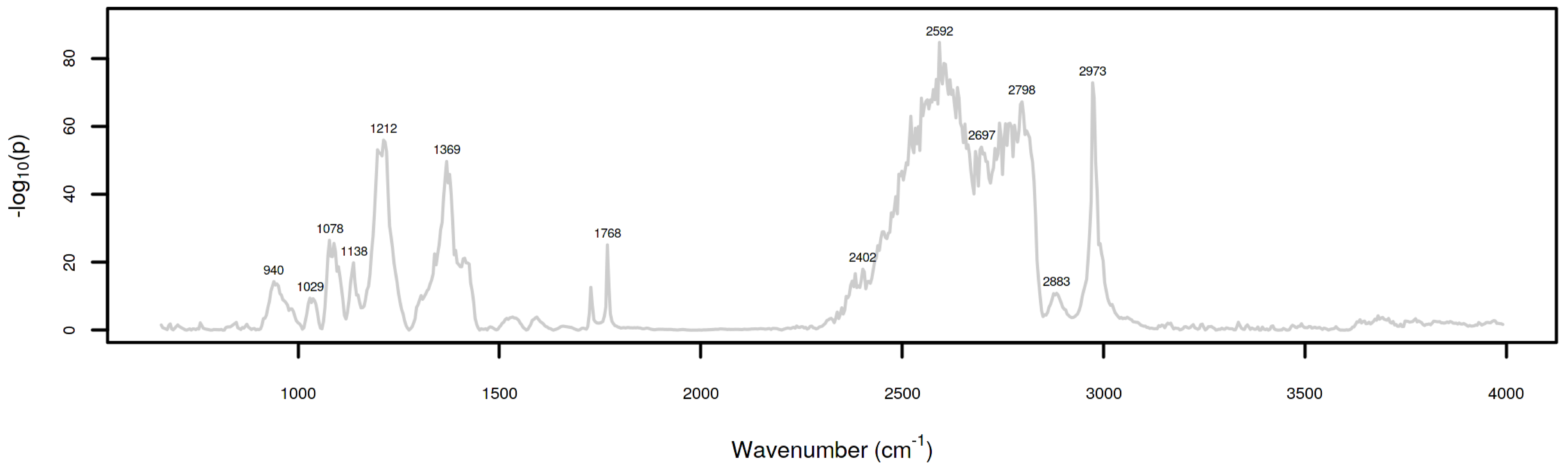

CSTB (Chr1:146481250)

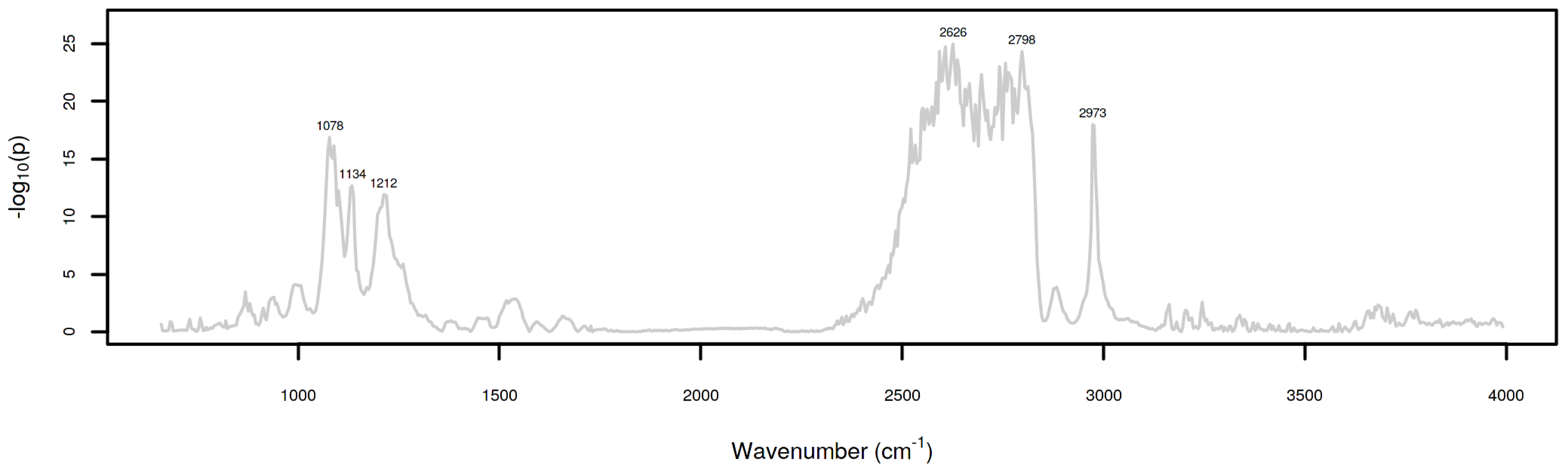

SH3BP5 (Chr1:154125158)

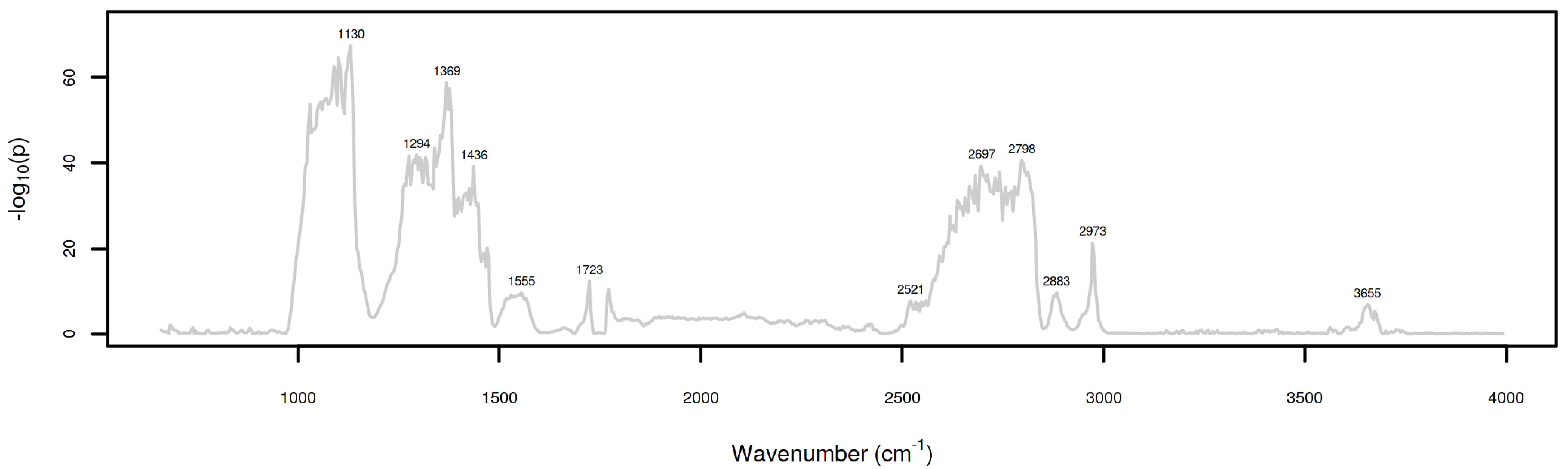

FCRLA (Chr3:7908611)

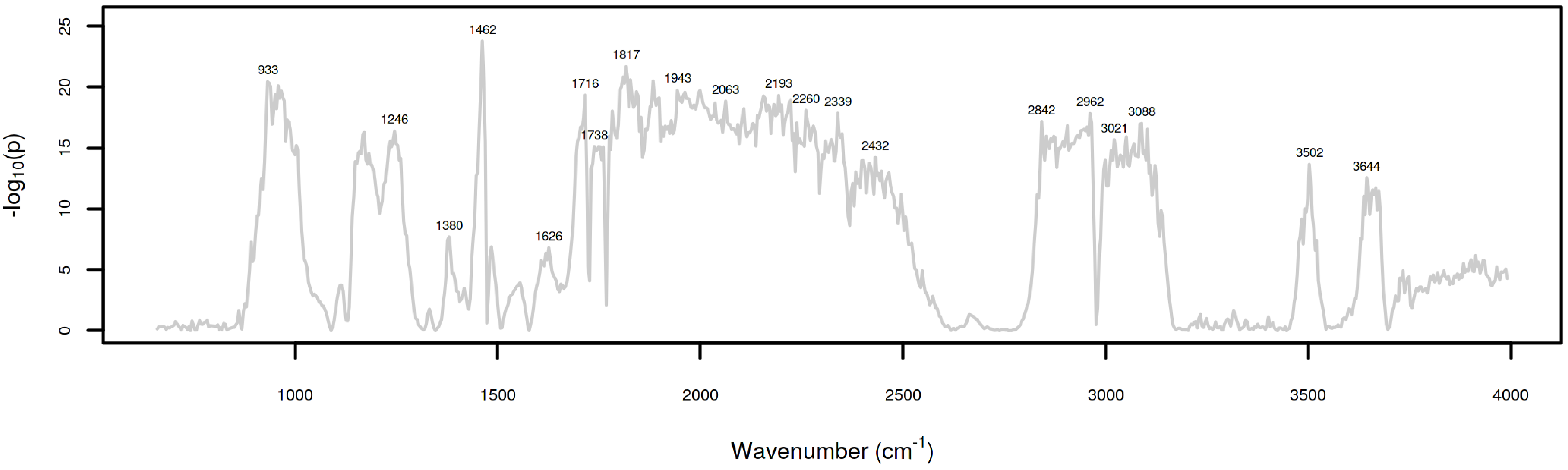

FCGR2B (Chr3:7931694)

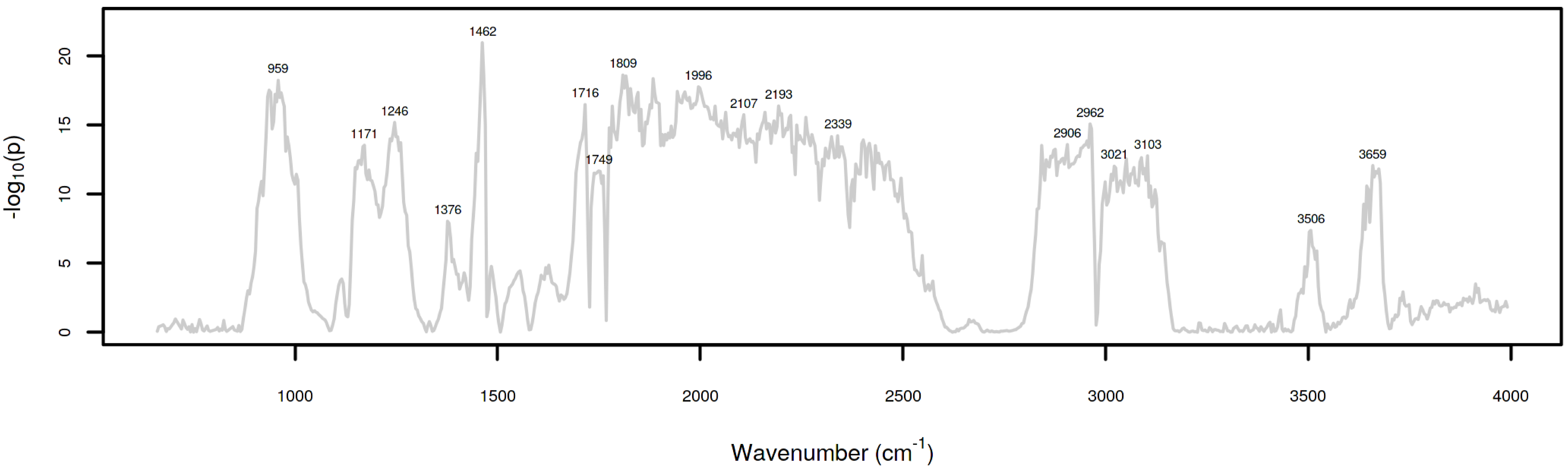

HCN3 (Chr3:15411459)

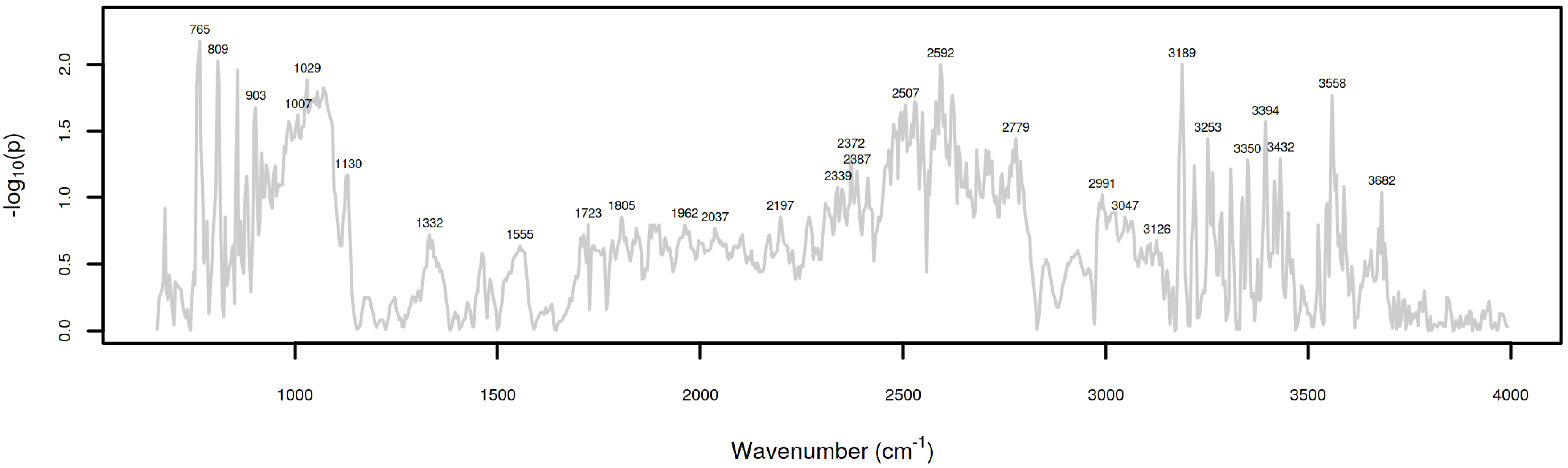

THBS3 (Chr3:15517871)

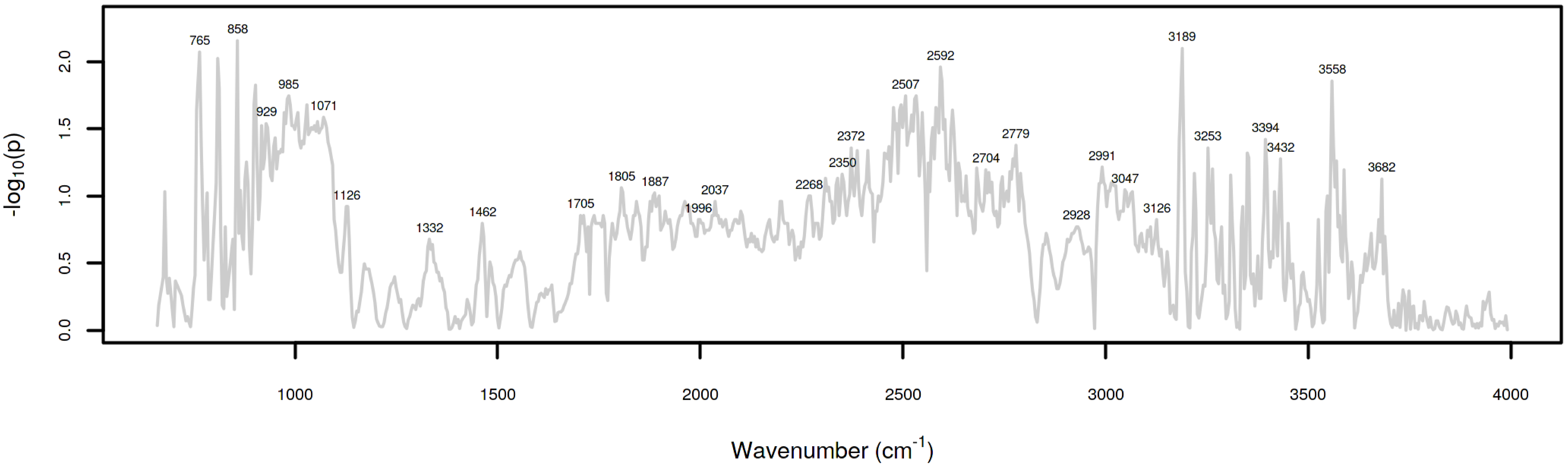

SLC50A1 (Chr3:15550598)

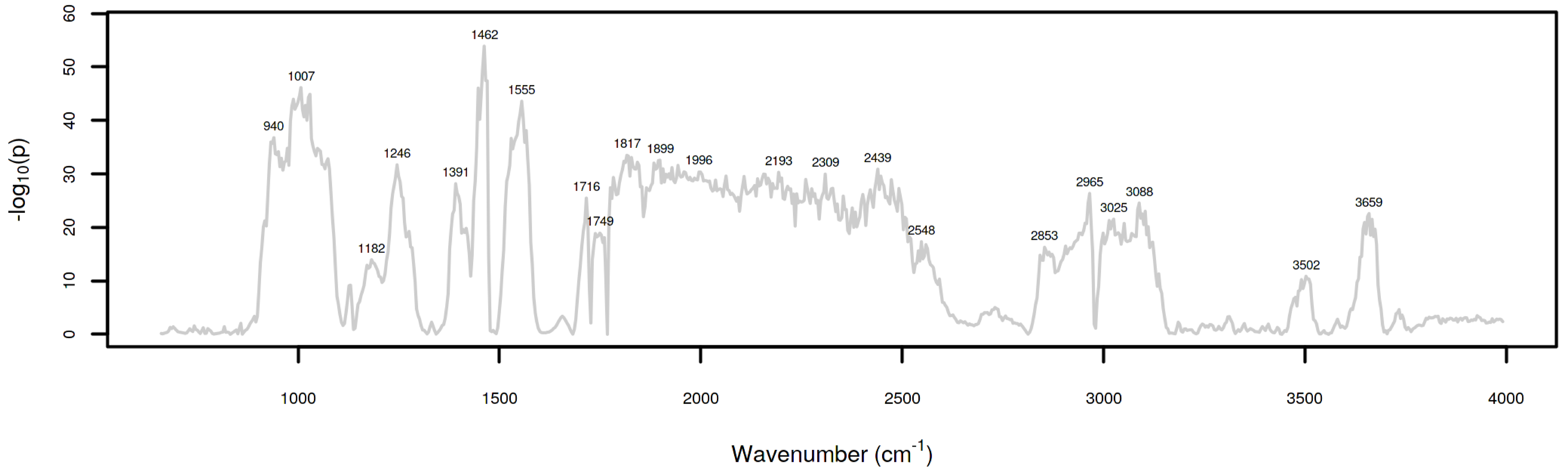

ELAPOR1 (Chr3:34387618)

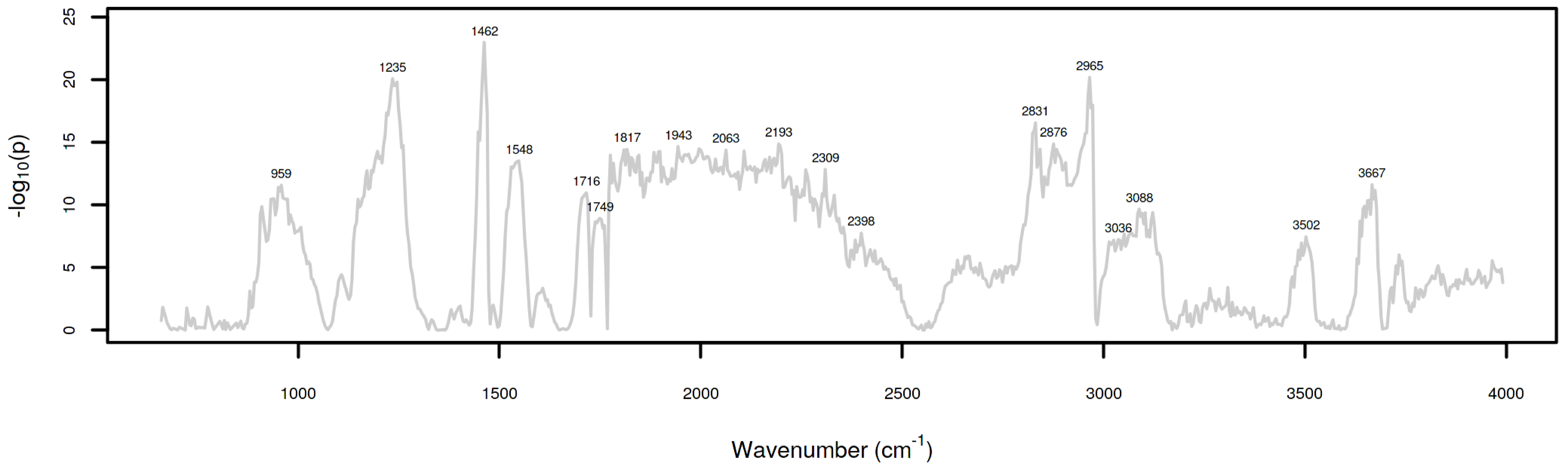

LRRC8C (Chr3:53755929)

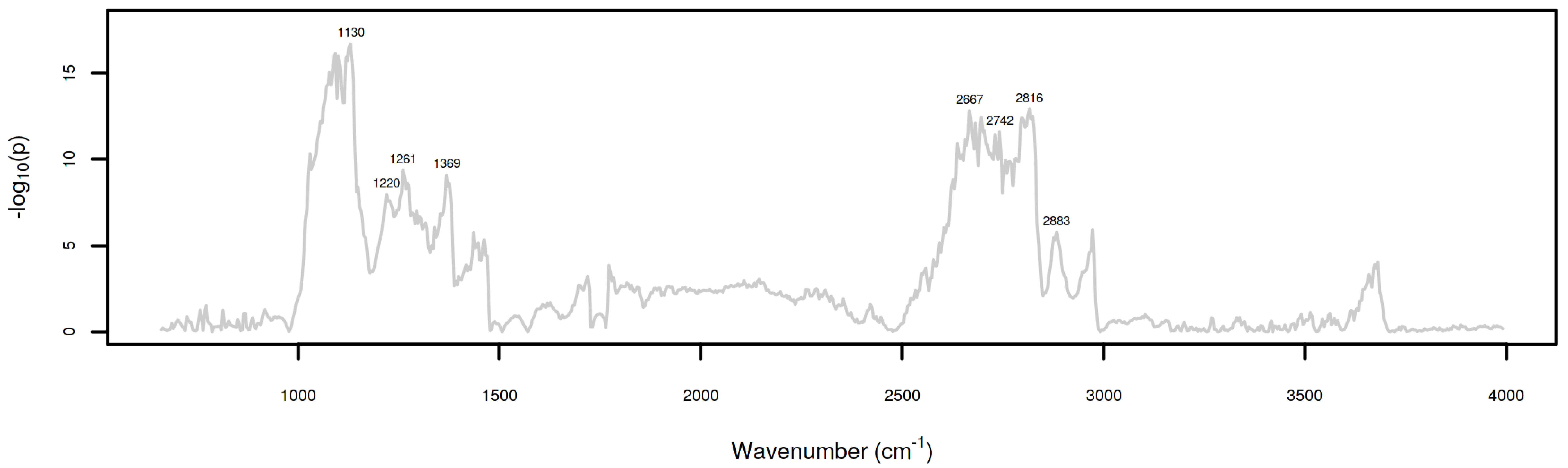

CSF2RB (Chr5:75729880)

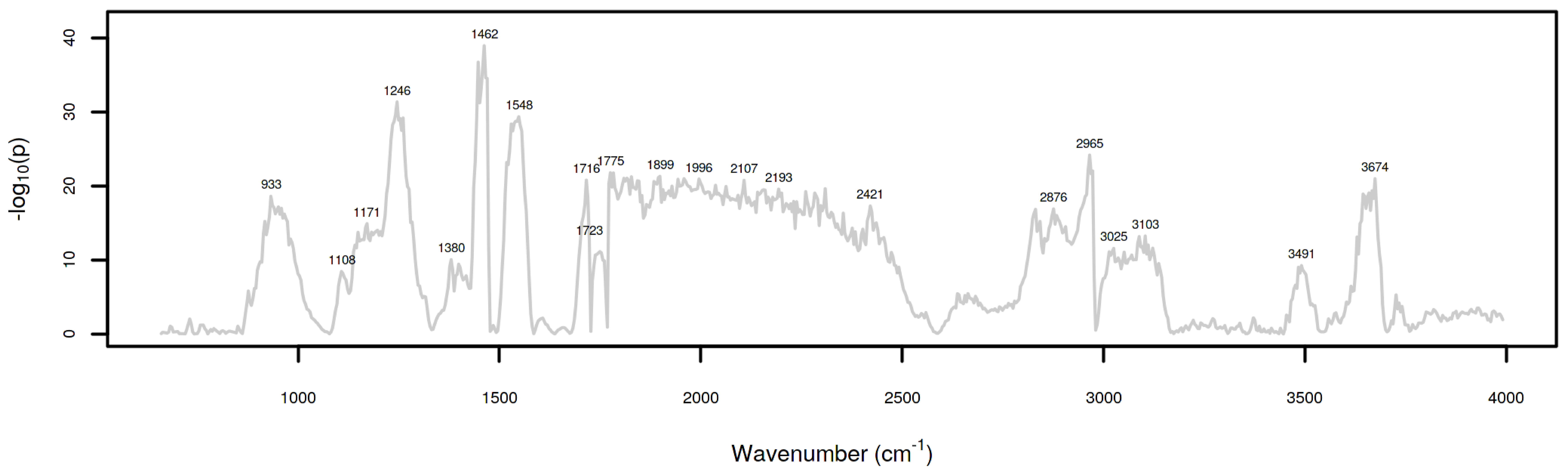

NCF4 (Chr5:75758989)

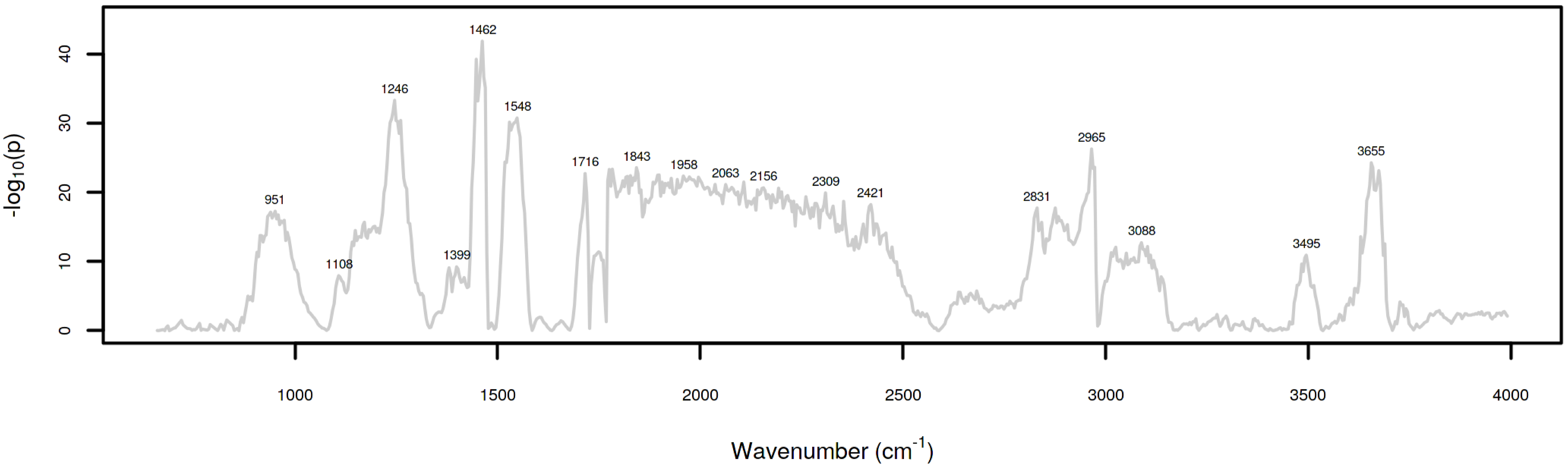

MGST1 (Chr5:93945738)

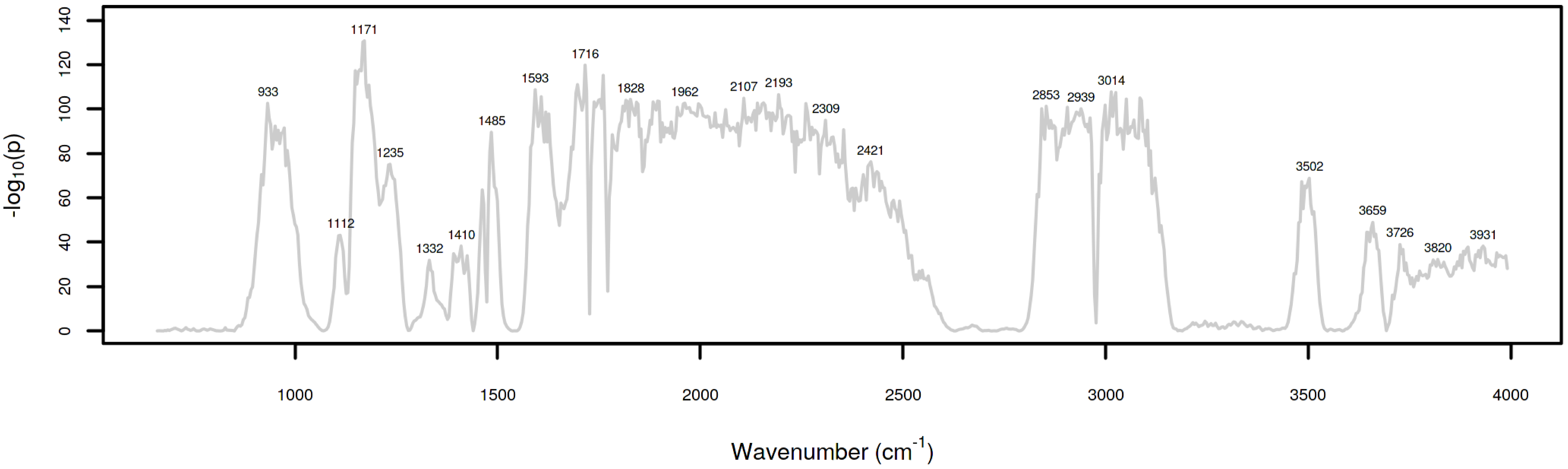

TBC1D22A (Chr5:118246868)

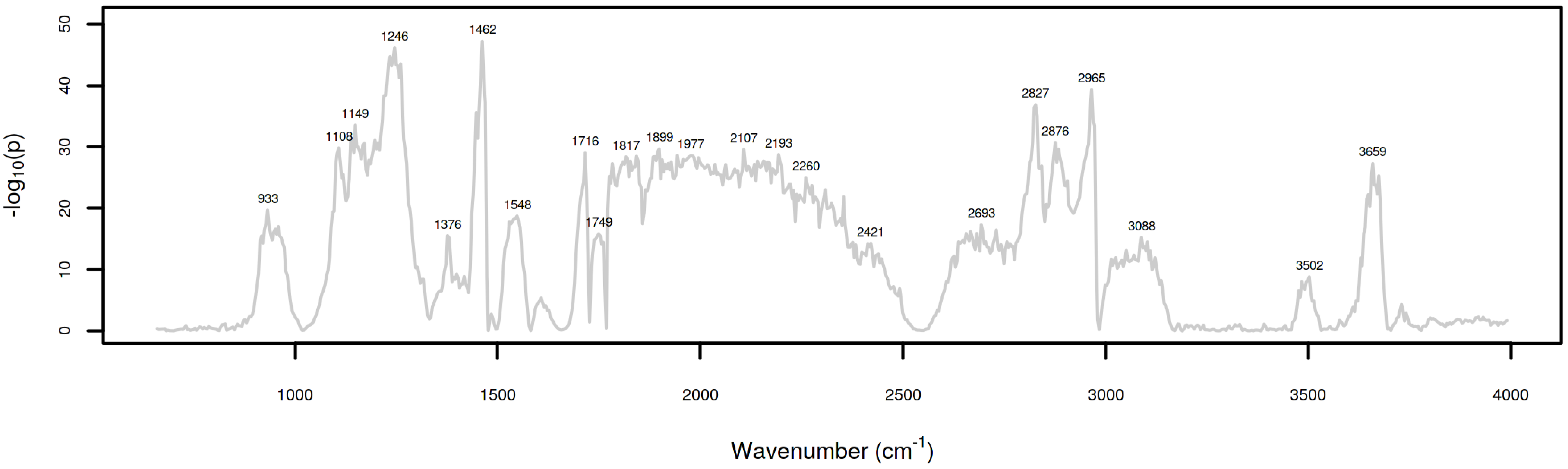

ABCG2 (Chr6:38027010)

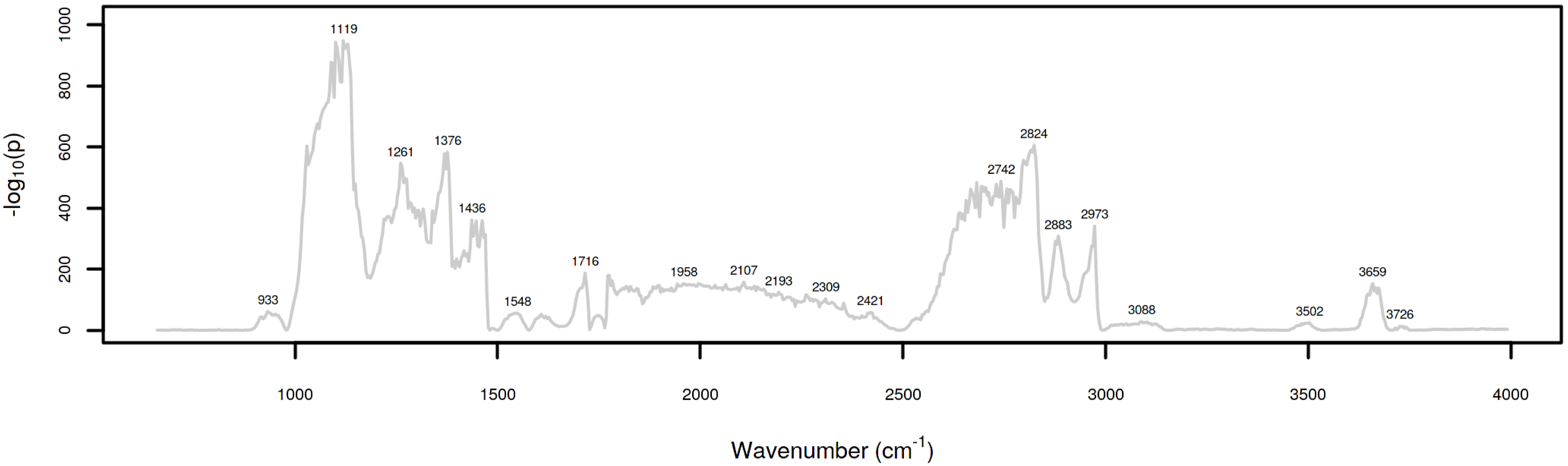

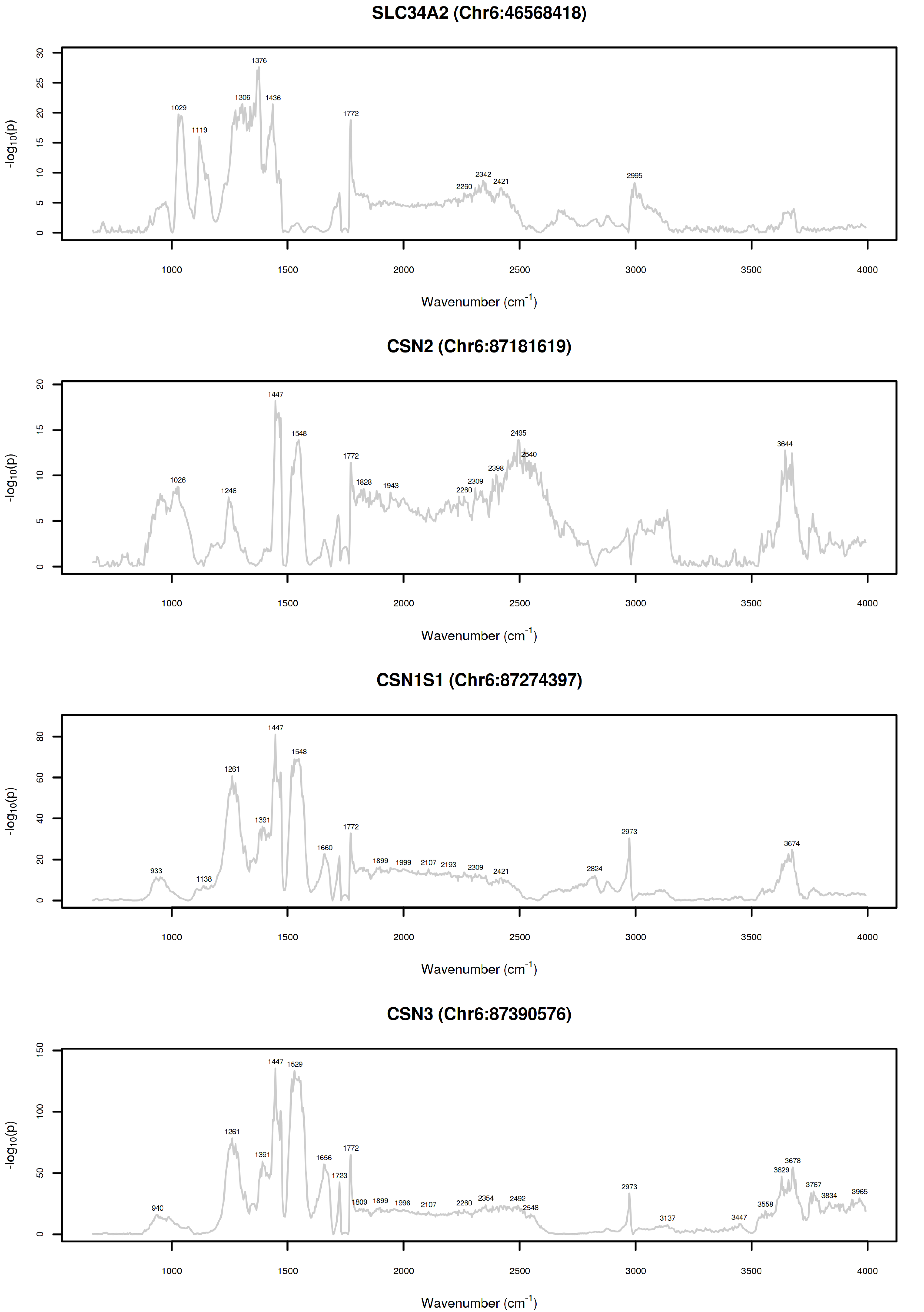
SLC34A2 (Chr6:46568418)
-log10(p)
1029
1119
1306
1376
1436
1772
2260
2342
2421
2995
Wavenumber (cm-1)
CSN2 (Chr6:87181619)
-log10(p)
1026
1246
1447
1548
1772
1828
1943
2260
2309
2398
2495
2540
3644
Wavenumber (cm-1)
CSN1S1 (Chr6:87274397)
-log10(p)
933
1138
1261
1391
1447
1548
1660
1772
1899
1999
2107
2193
2309
2421
2824
2973
3674
Wavenumber (cm-1)
CSN3 (Chr6:87390576)
-log10(p)
940
1261
1391
1447
1529
1656
1723
1772
1809
1899
1996
2107
2260
2354
2492
2548
2973
3137
3447
3558
3629
3678
3767
3834
3965
Wavenumber (cm-1)

TENT5A (Chr9:21637056)

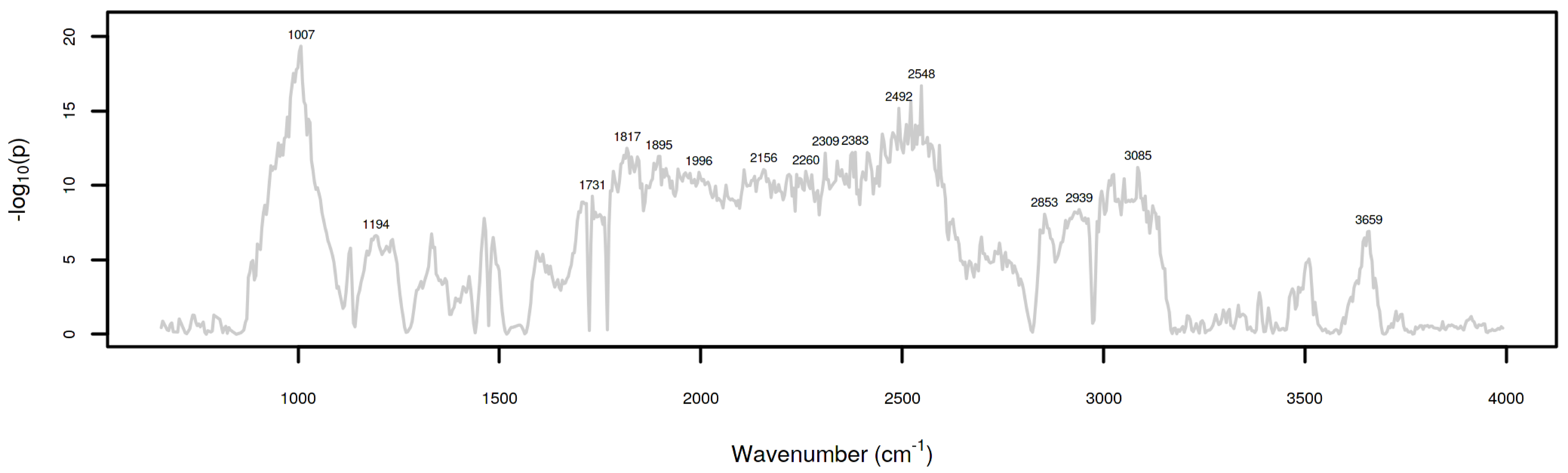

RNF217 (Chr9:26534109)

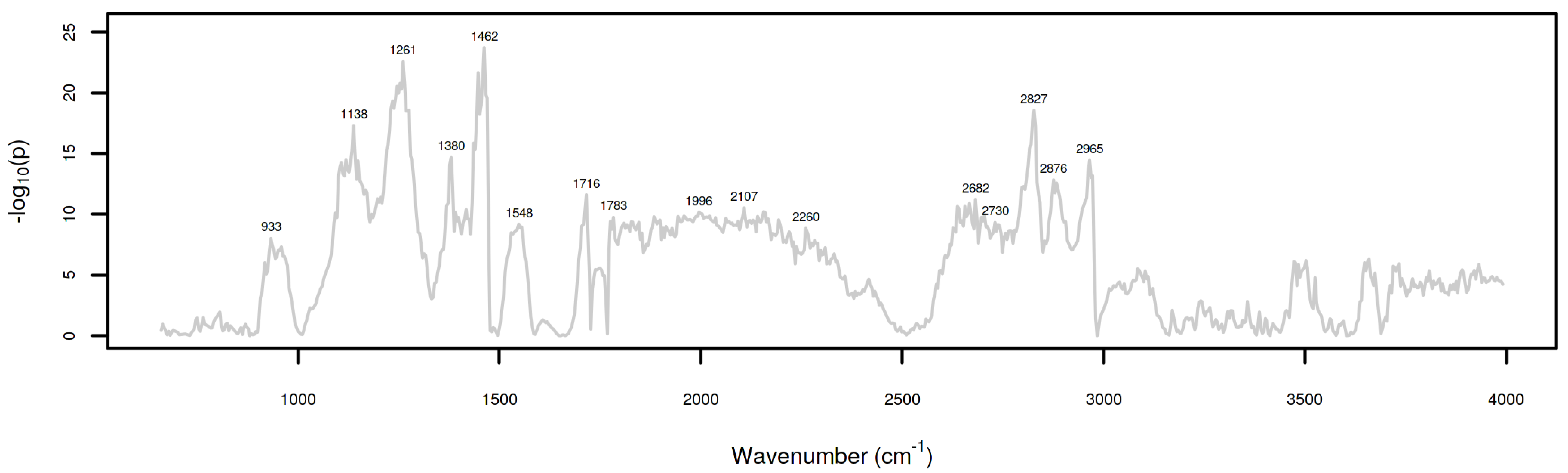

TAB2 (Chr9:87585031)

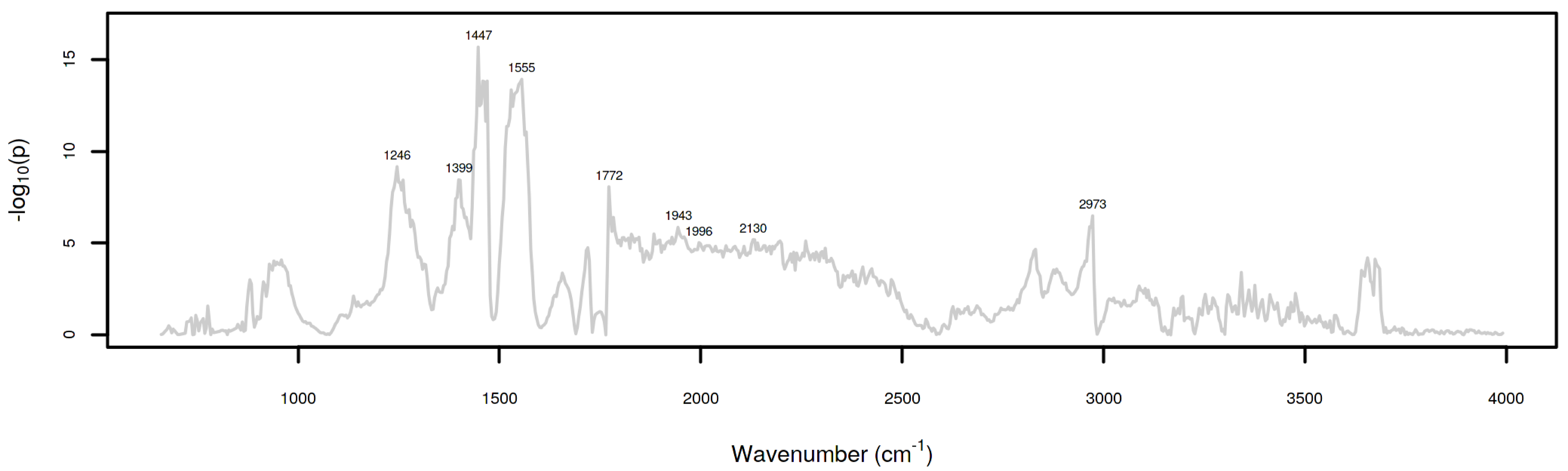

MPC1 (Chr9:102874726)

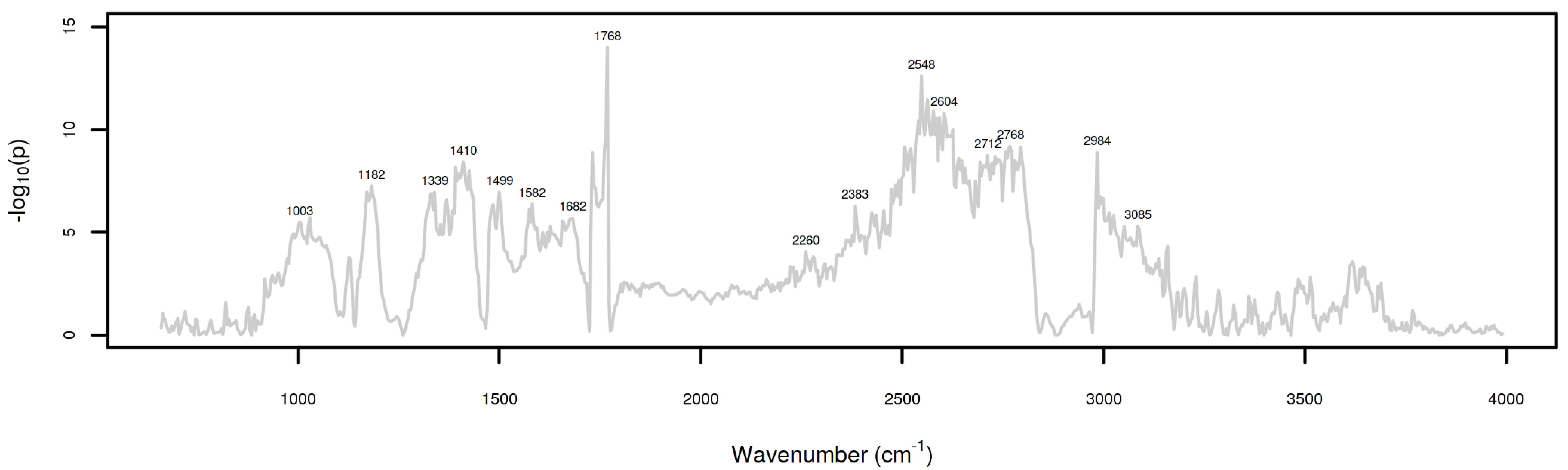

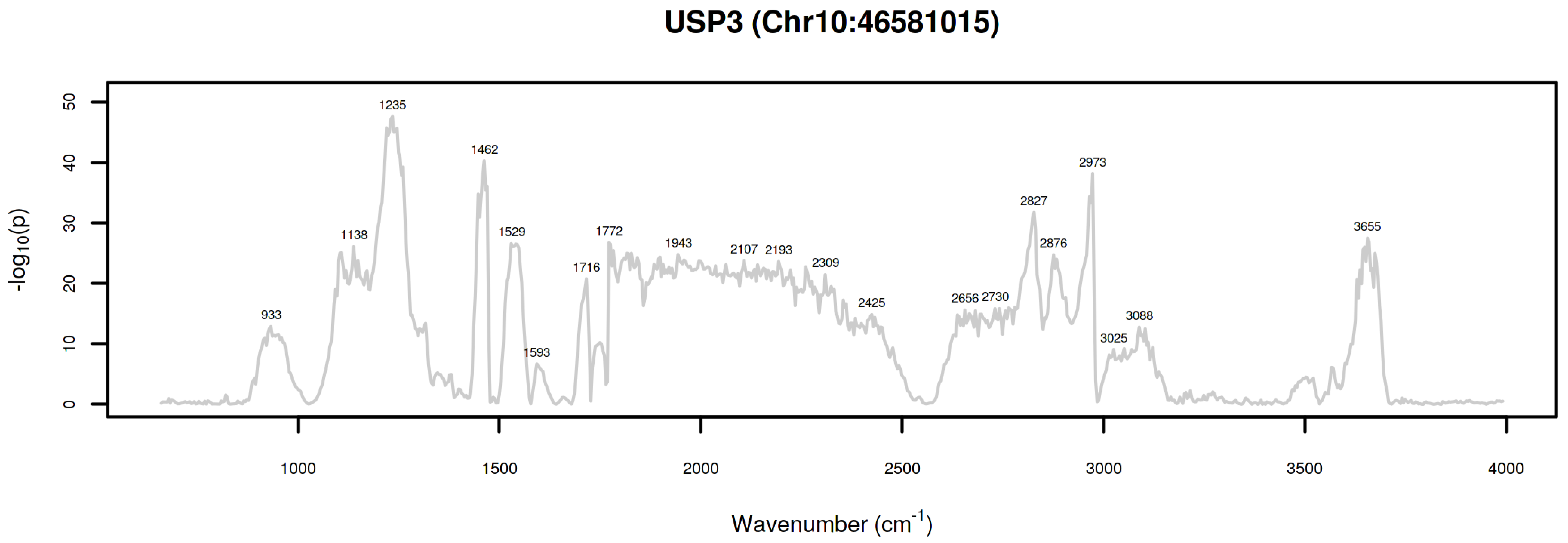
USP3 (Chr10:46581015)
-log10(p)
Wavenumber (cm-1)
933
1138
1235
1462
1529
1593
1716
1772
1943
2107
2193
2309
2425
2656
2730
2827
2876
2973
3025
3088
3655

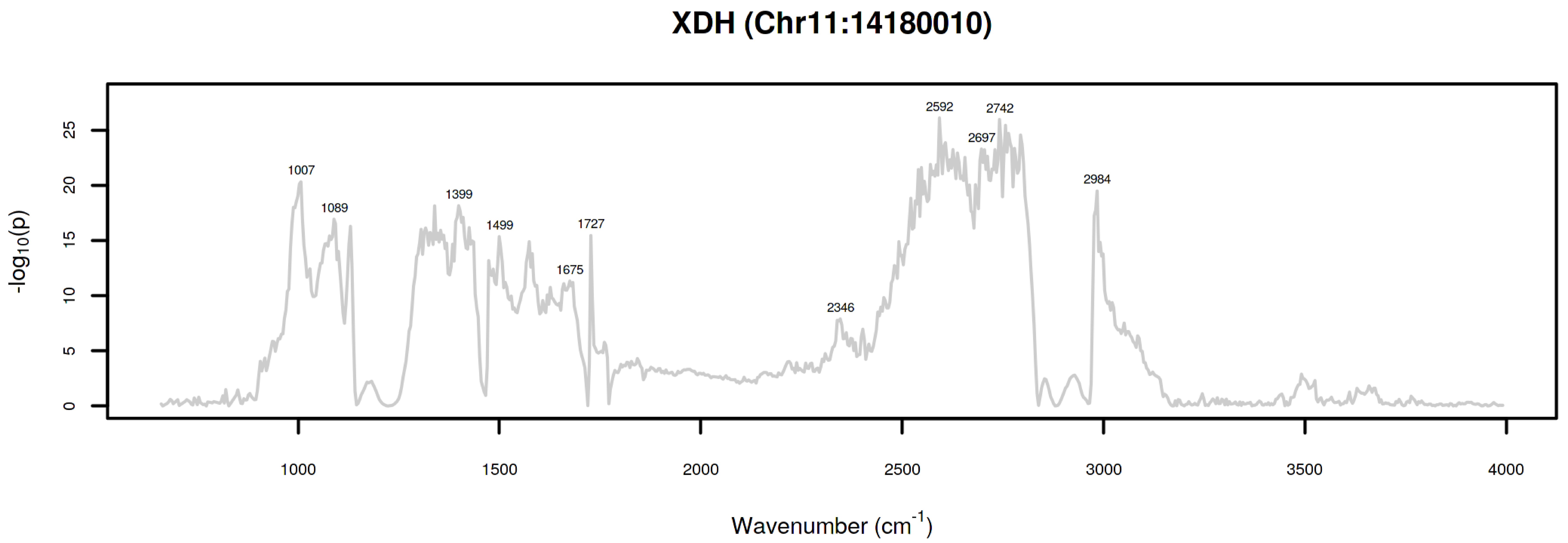
XDH (Chr11:14180010)
-log10(p)
Wavenumber (cm-1)
1007
1089
1399
1499
1675
1727
2346
2592
2697
2742
2984

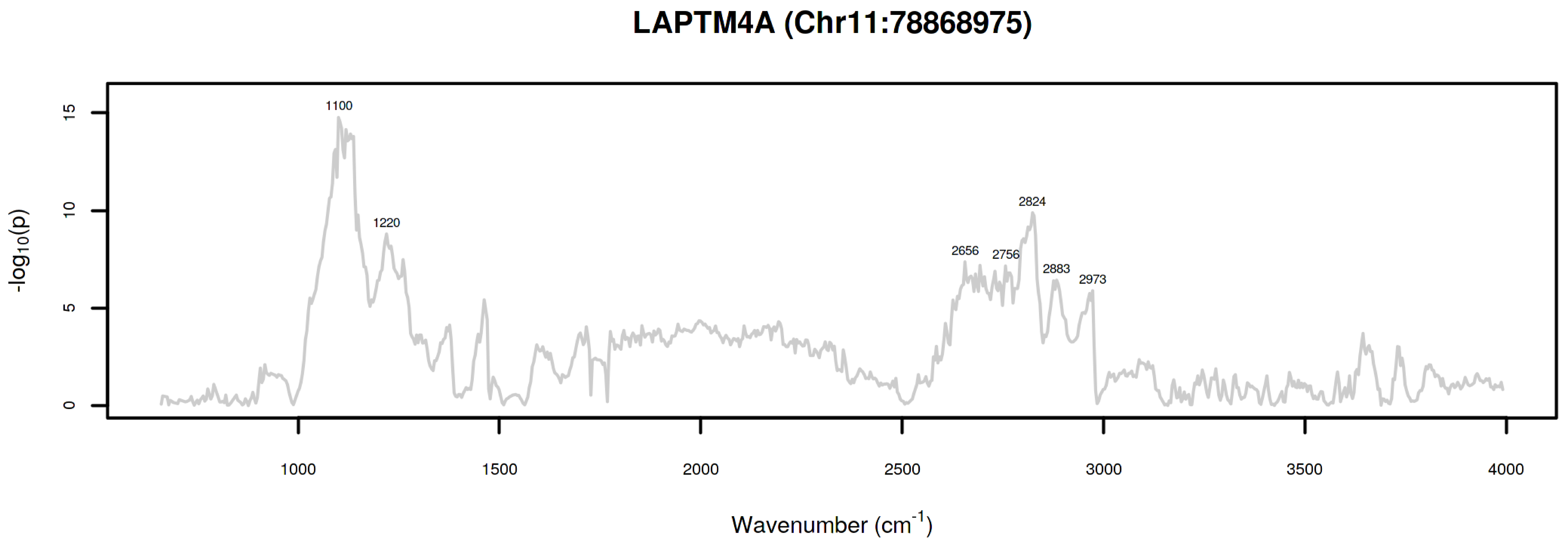
LAPTM4A (Chr11:78868975)
-log10(p)
Wavenumber (cm-1)
1100
1220
2656
2756
2824
2883
2973

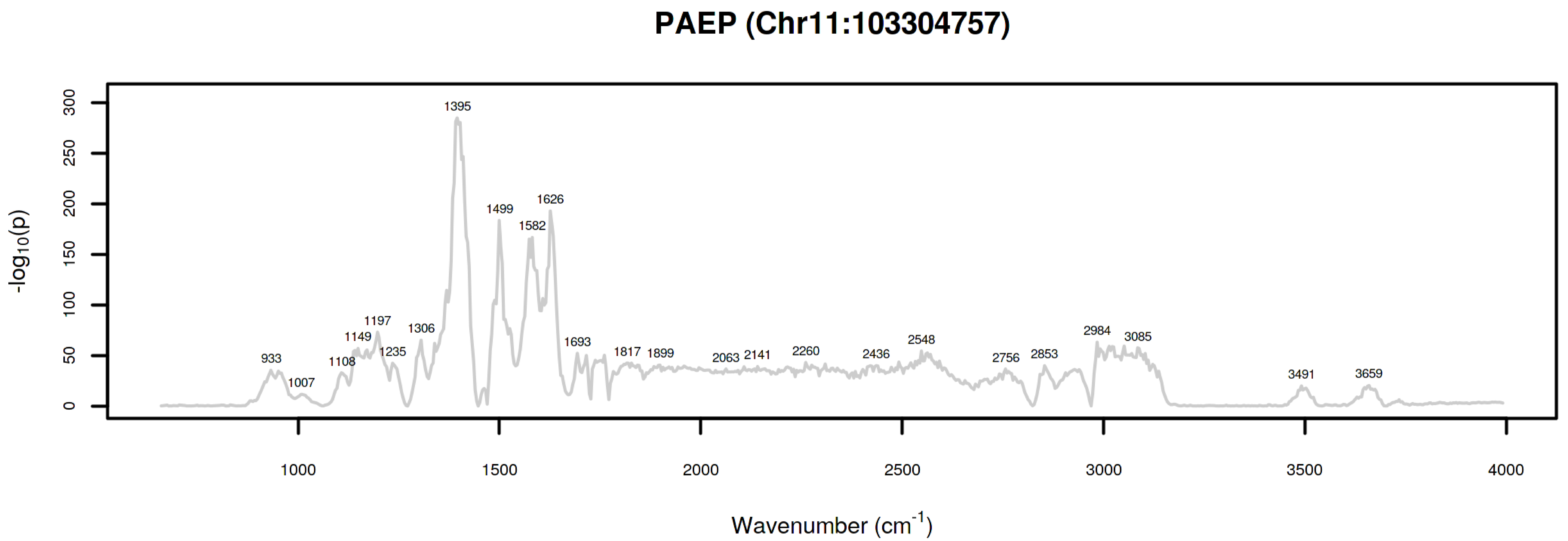
PAEP (Chr11:103304757)
-log10(p)
Wavenumber (cm-1)
933
1007
1108
1149
1197
1235
1306
1395
1499
1582
1626
1693
1817
1899
2063
2141
2260
2436
2548
2756
2853
2984
3085
3491
3659

ABO (Chr11:104242578)

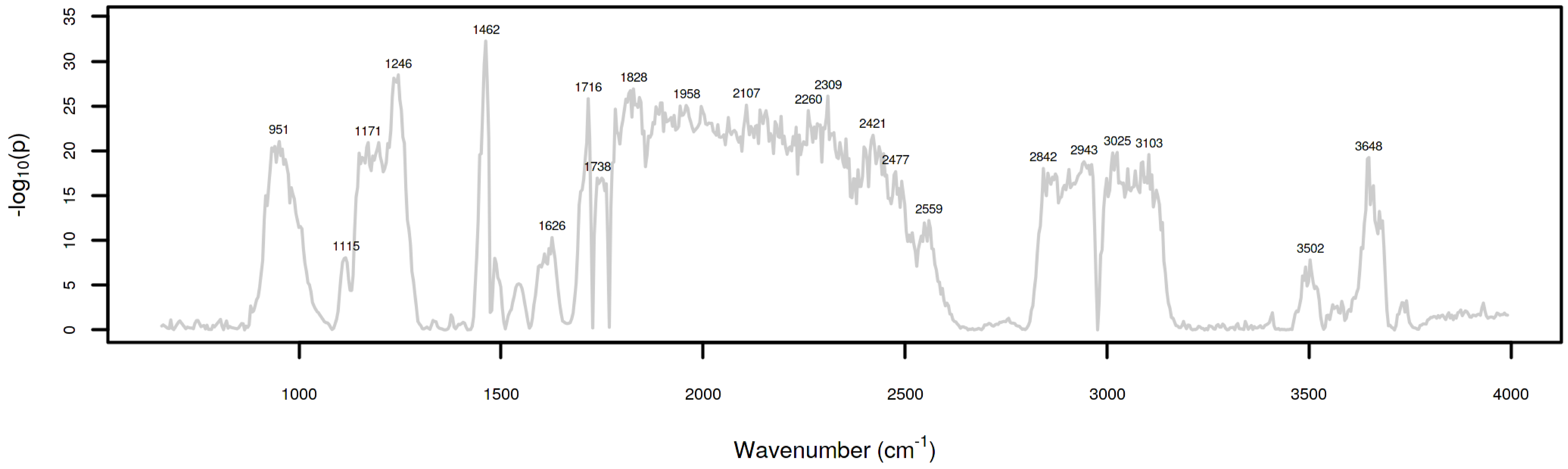

TGDS (Chr12:69612955)

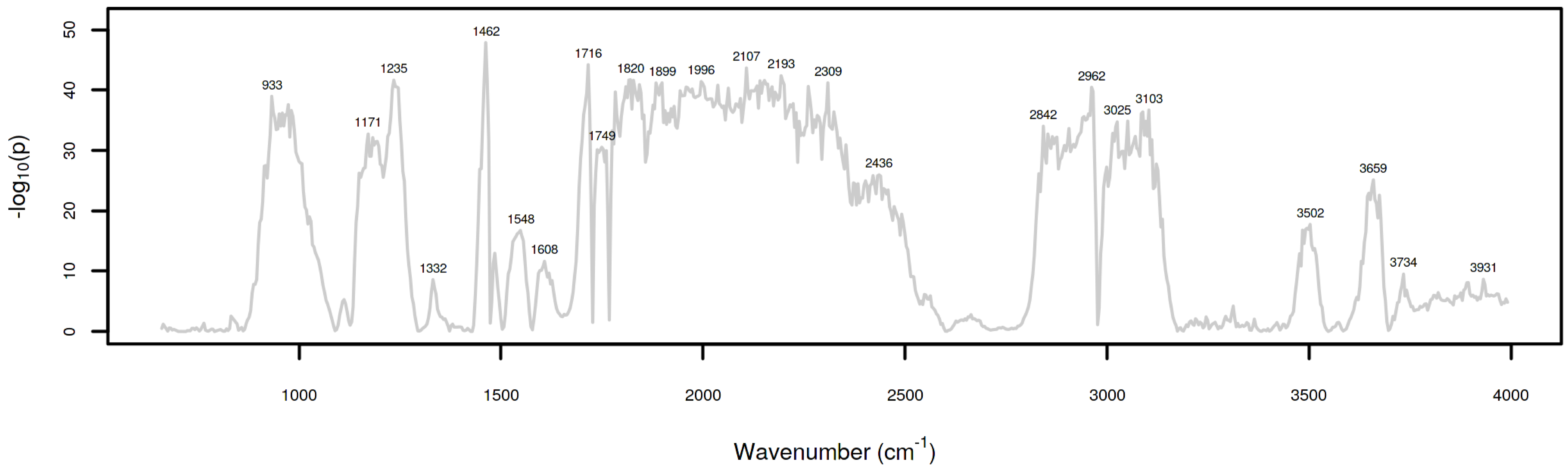

WDR97 (Chr14:1726650)

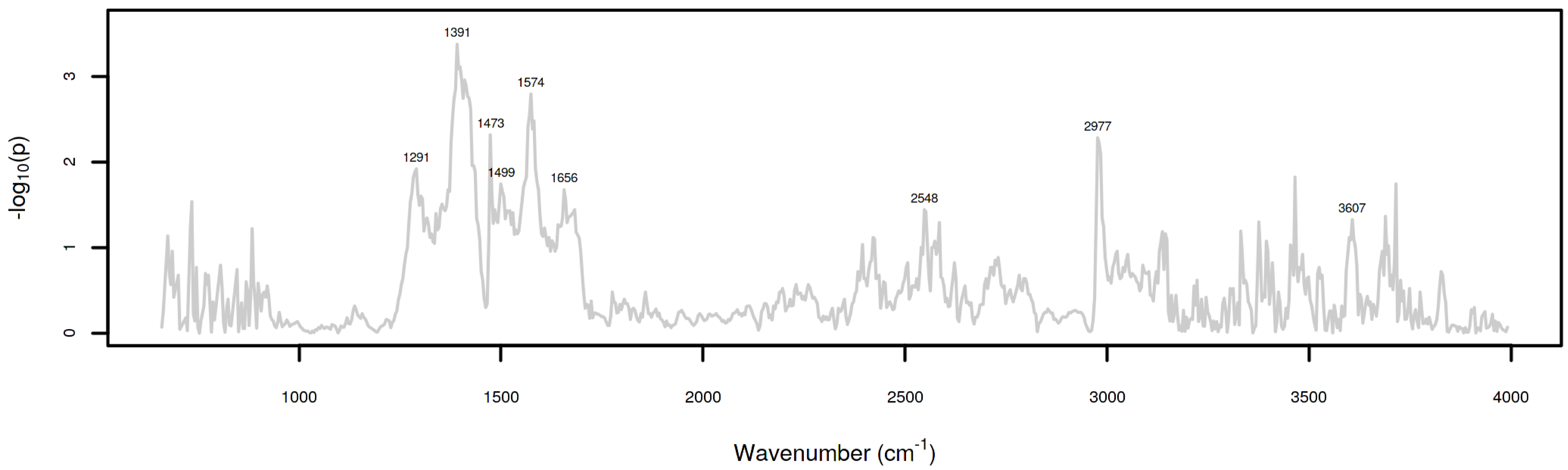

MROH1 (Chr14:1732043)

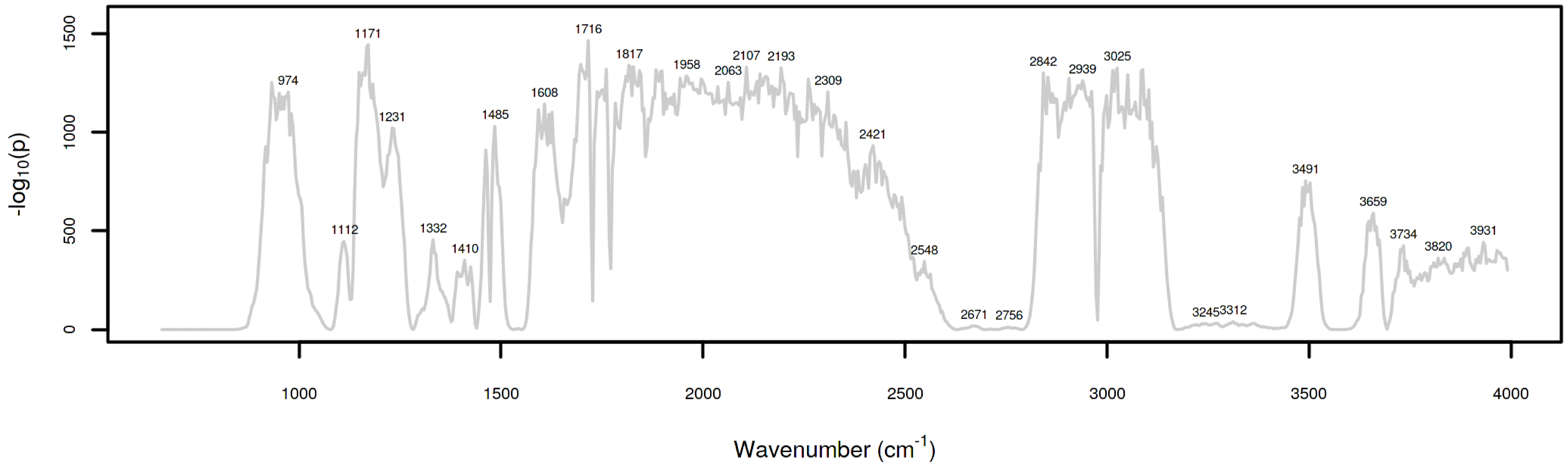

CPSF1 (Chr14:1755742)

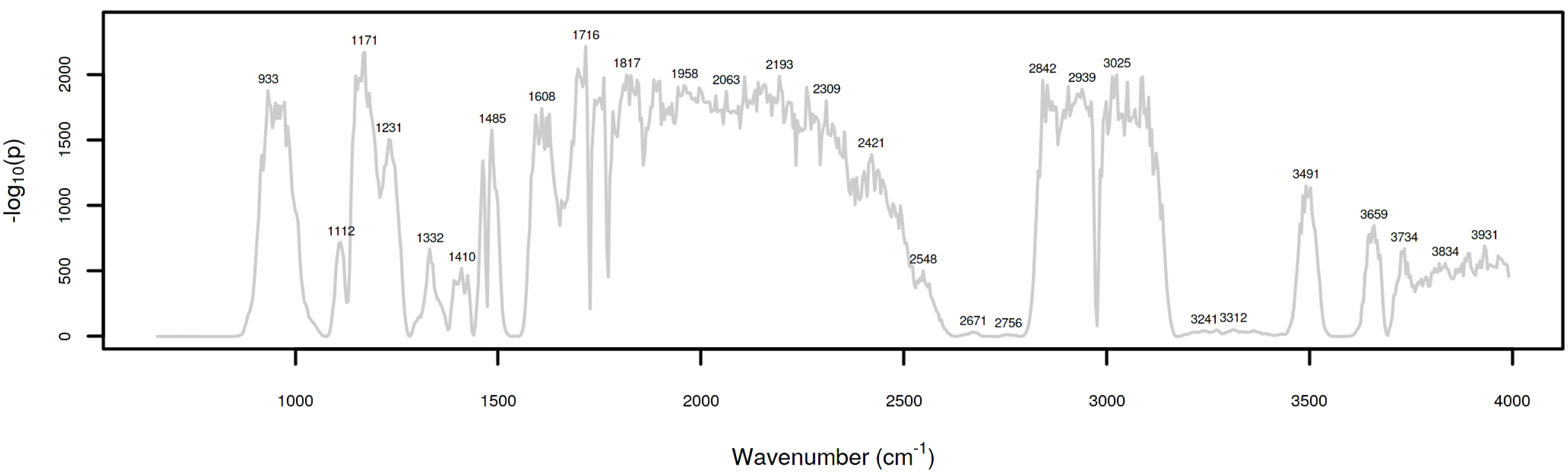

DGAT1 (Chr14:1802265)

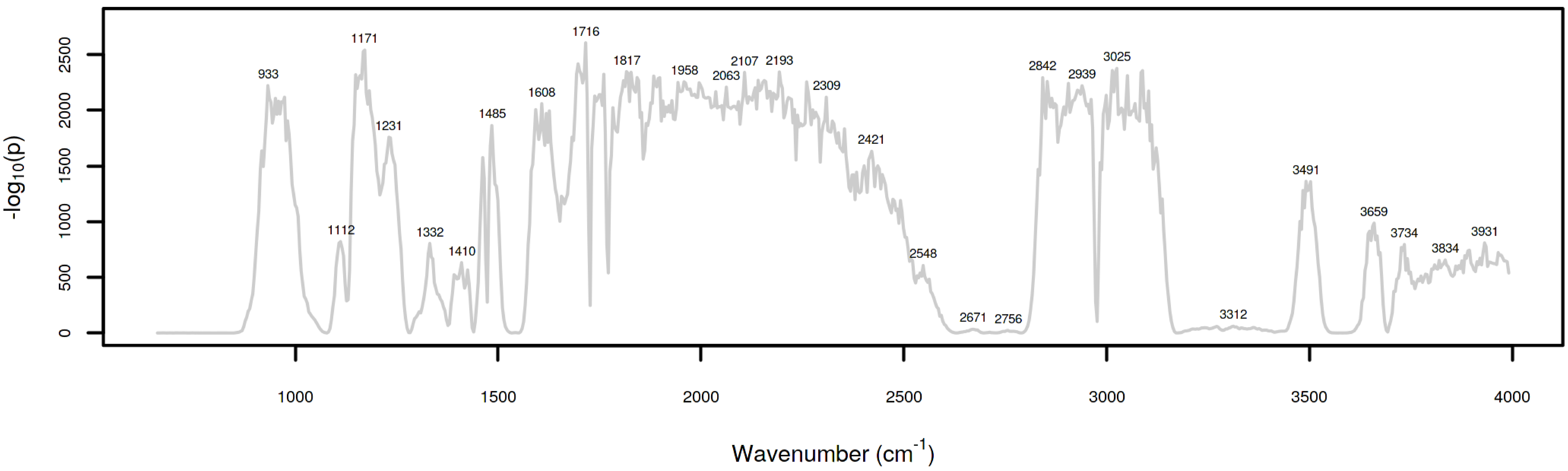

SPAG1 (Chr14:66328304)

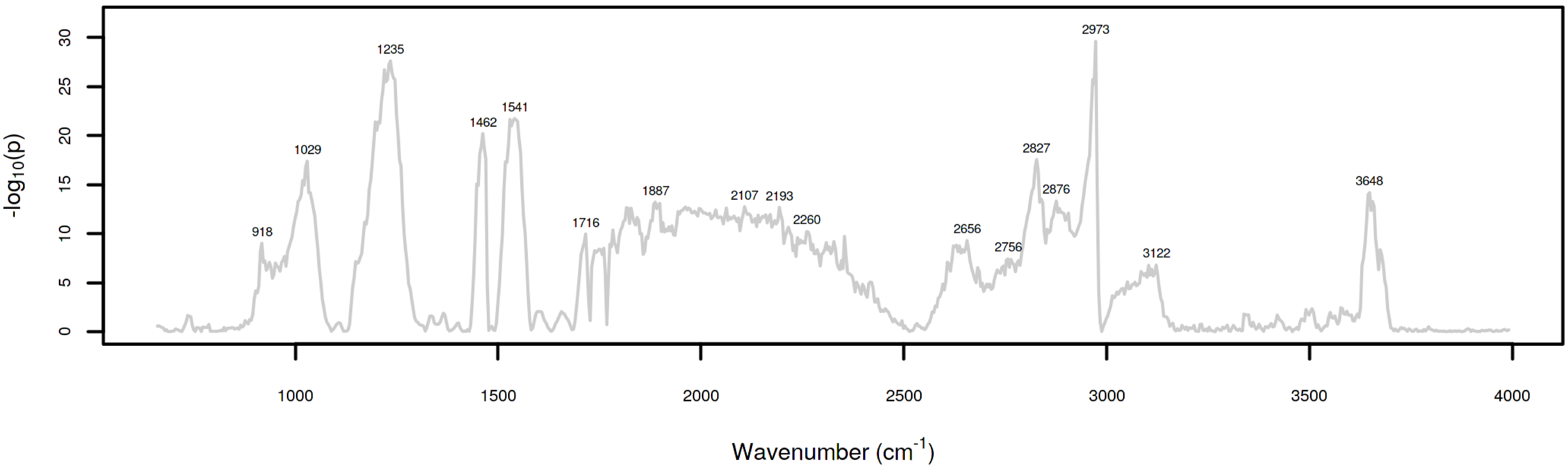

RNF214 (Chr15:28347165)

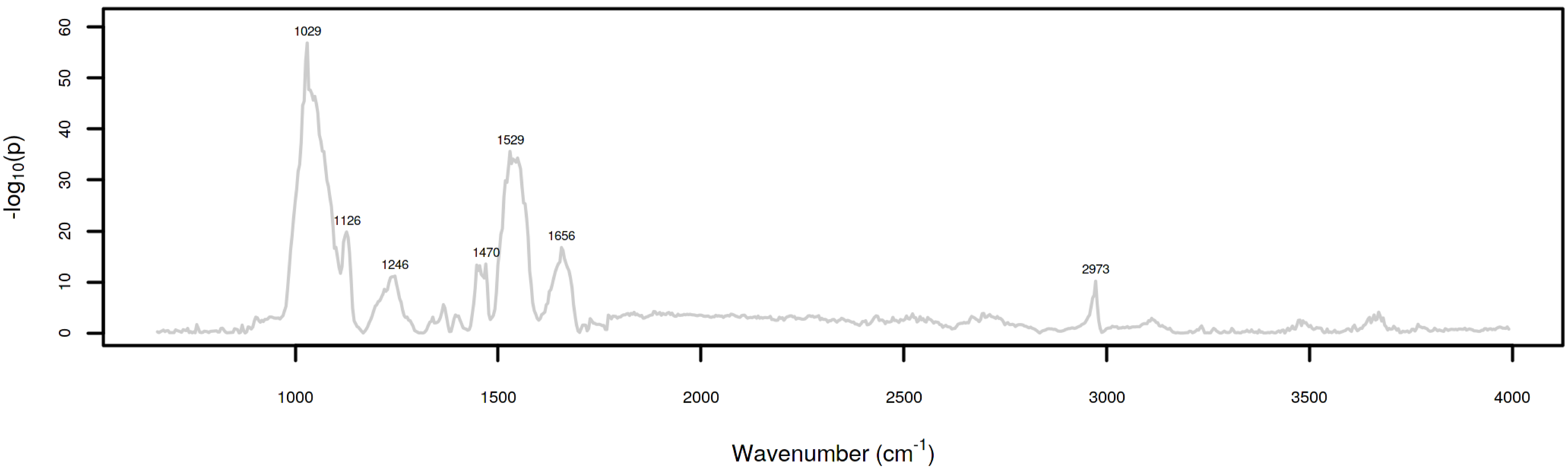

DNAJB13 (Chr15:53940444)

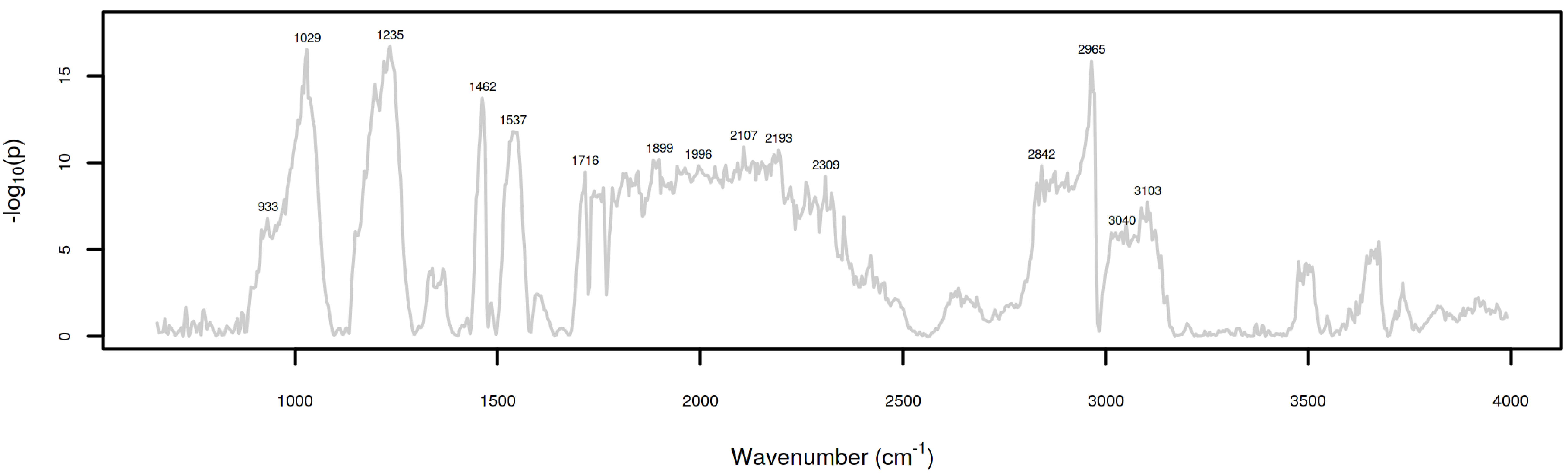

CAPN5 (Chr15:57266467)

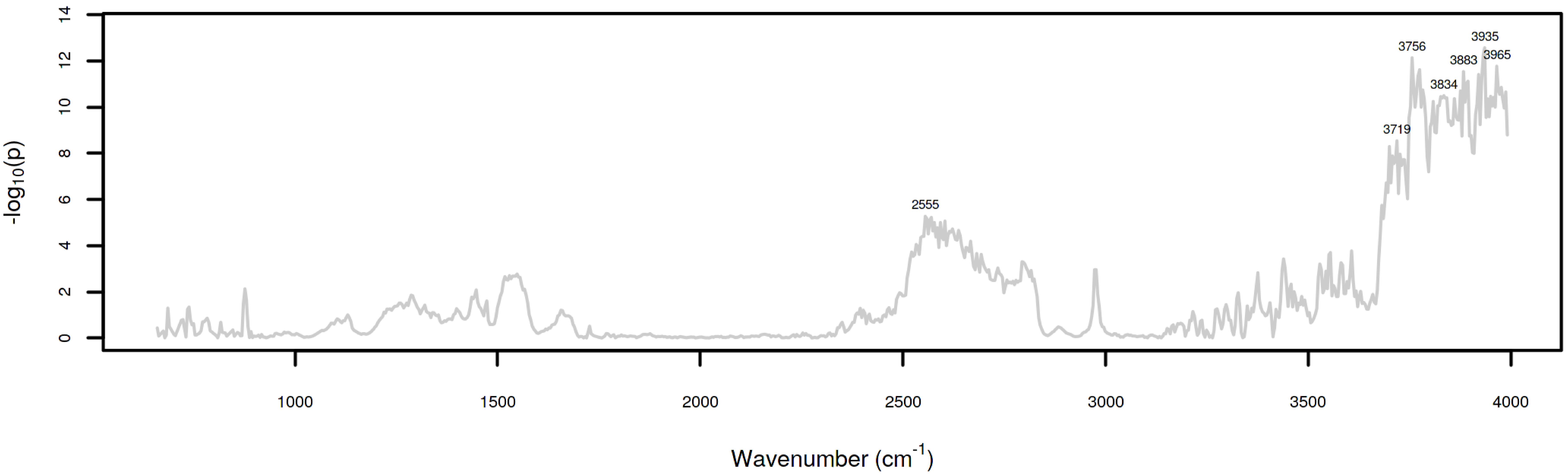

MTARC1 (Chr16:24977696)

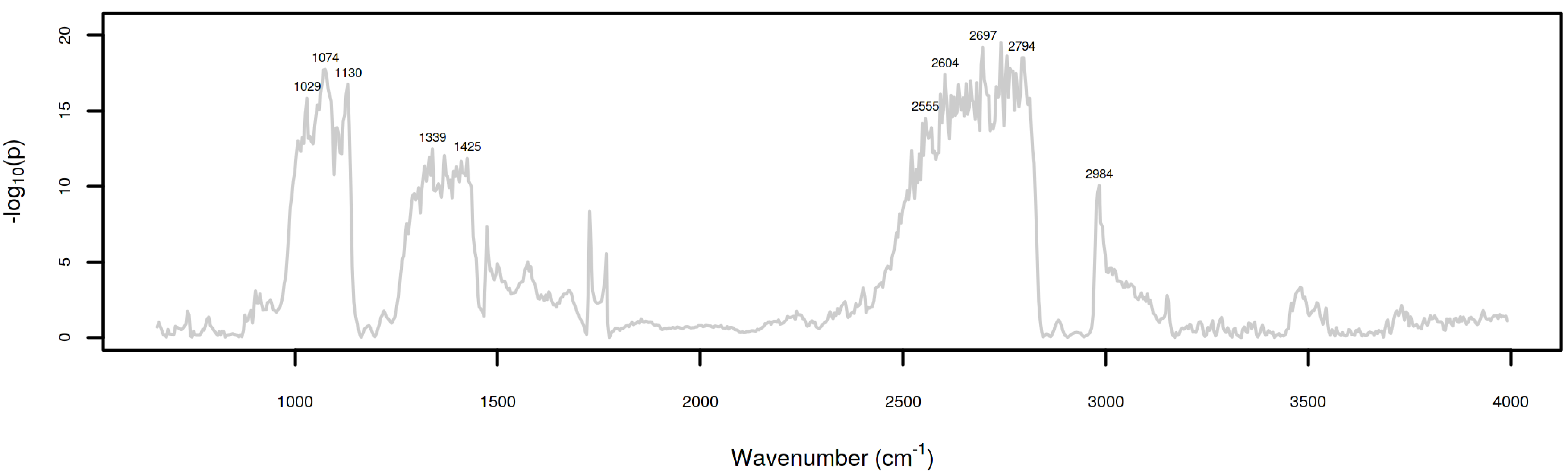

RGL1 (Chr16:66314547)

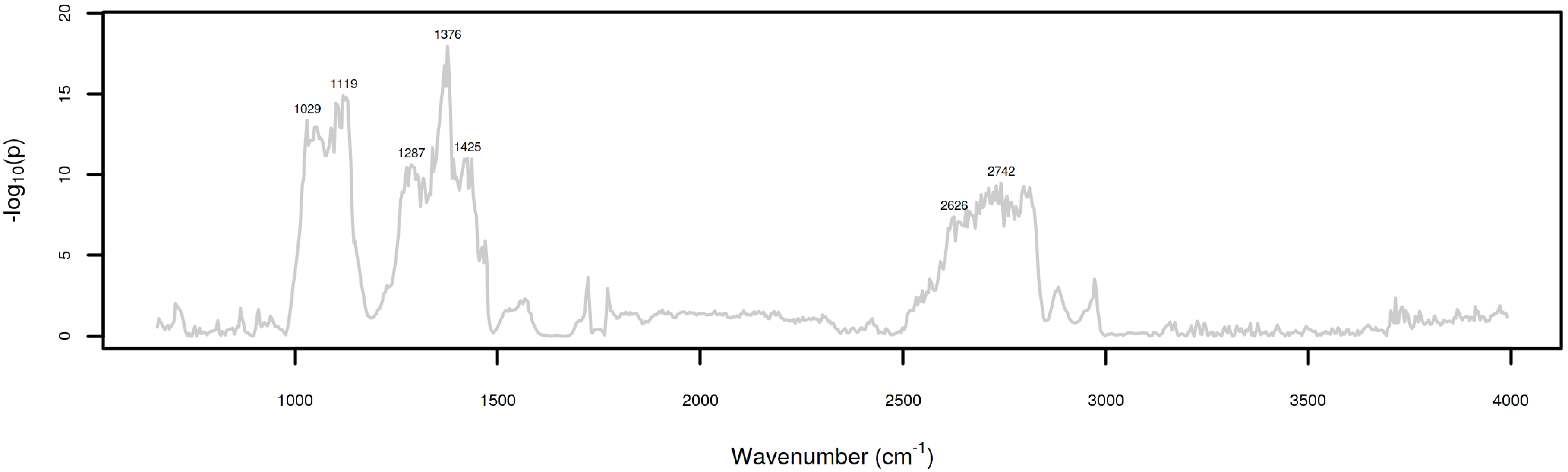

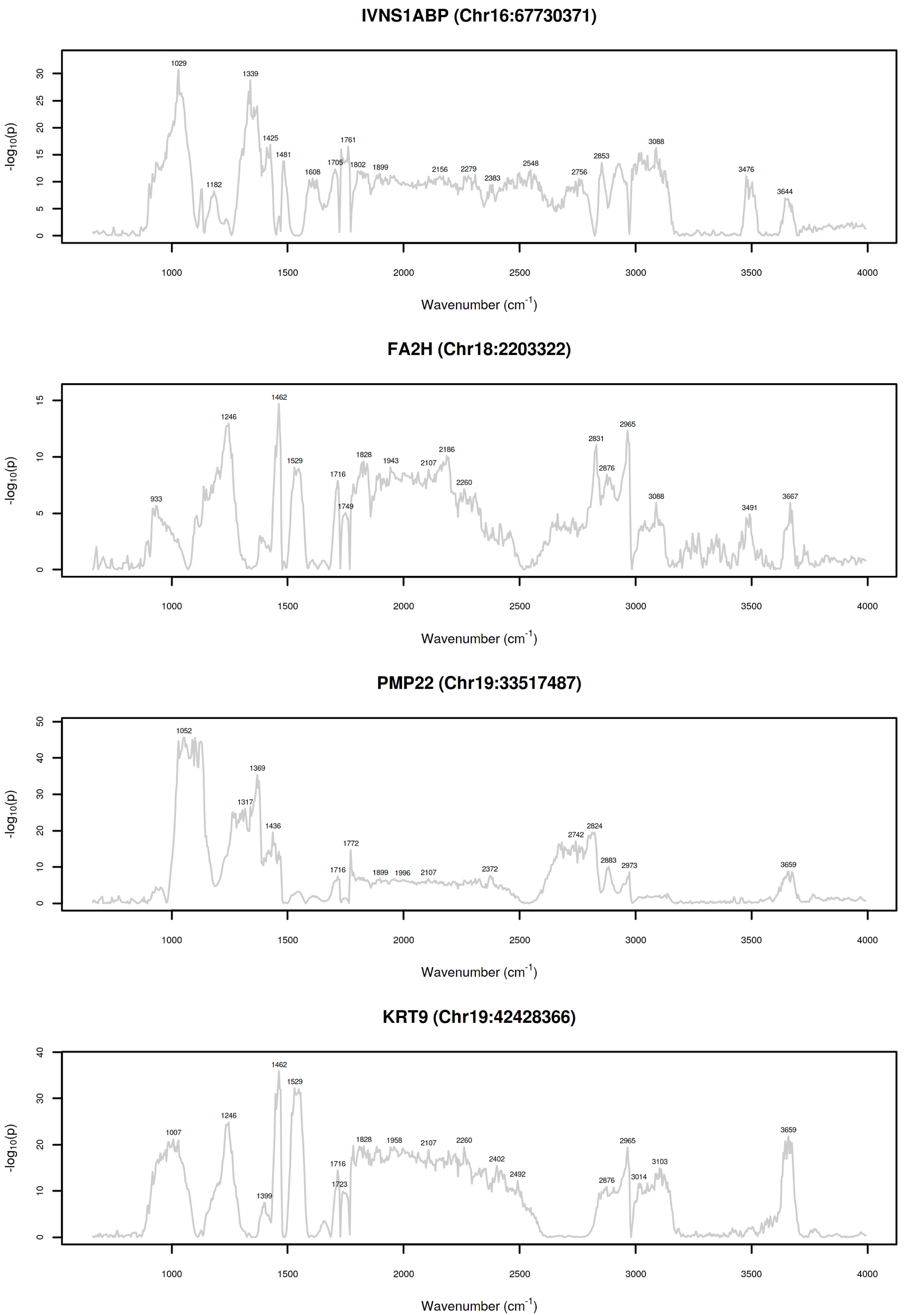

IVNS1ABP (Chr16:67730371)
1029
1339
1182
1425
1481
1608
1705
1761
1802
1899
2156
2279
2383
2548
2756
2853
3088
3476
3644
FA2H (Chr18:2203322)
933
1246
1462
1529
1716
1749
1828
1943
2107
2186
2260
2831
2876
2965
3088
3491
3667
PMP22 (Chr19:33517487)
1052
1317
1369
1436
1716
1772
1899
1996
2107
2372
2742
2824
2883
2973
3659
KRT9 (Chr19:42428366)
1007
1246
1399
1462
1529
1716
1723
1828
1958
2107
2260
2402
2492
2876
2965
3014
3103
3659
-log10(p)
Wavenumber (cm-1)

KRT42 (Chr19:42488389)

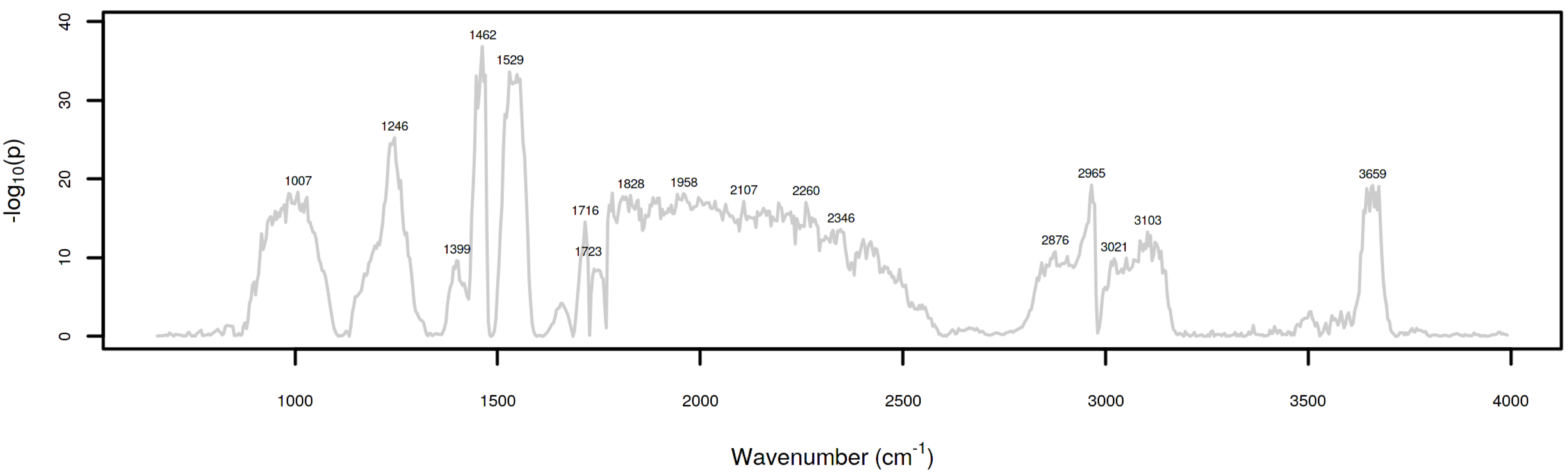

KCNH4 (Chr19:43036265)

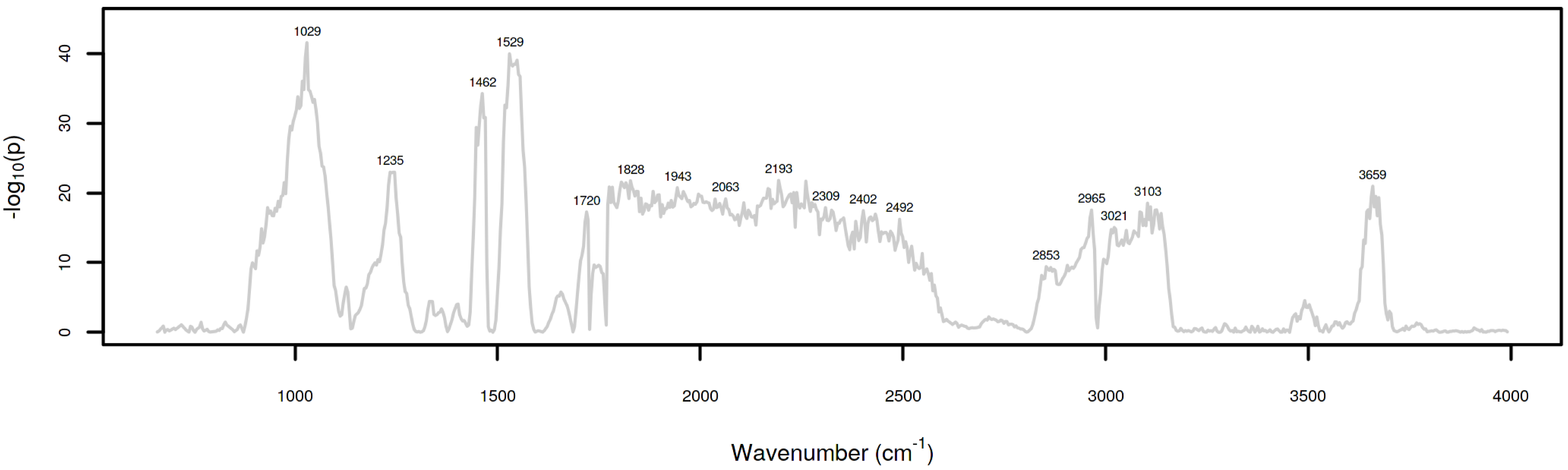

STAT5A (Chr19:43053995)

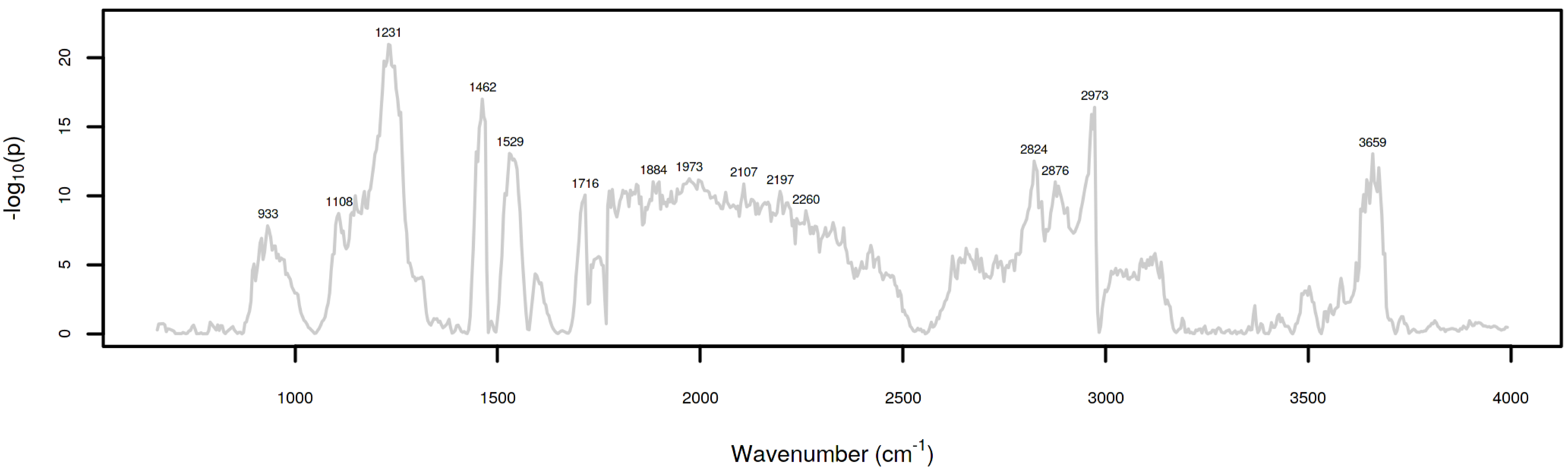

CCDC57 (Chr19:51303887)

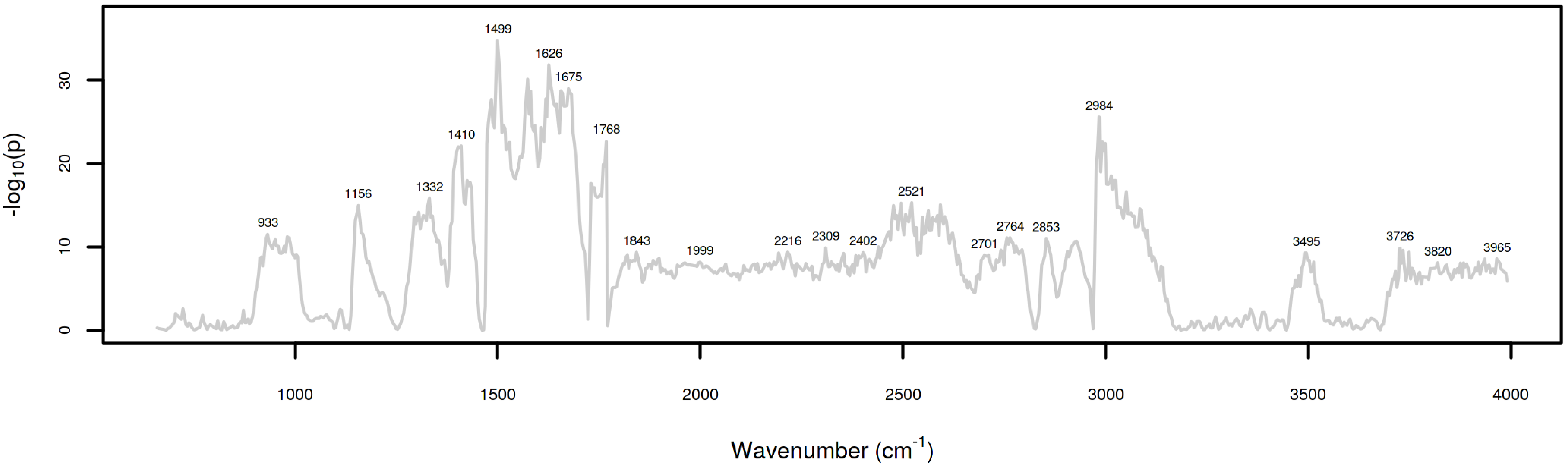

HID1 (Chr19:57087981)

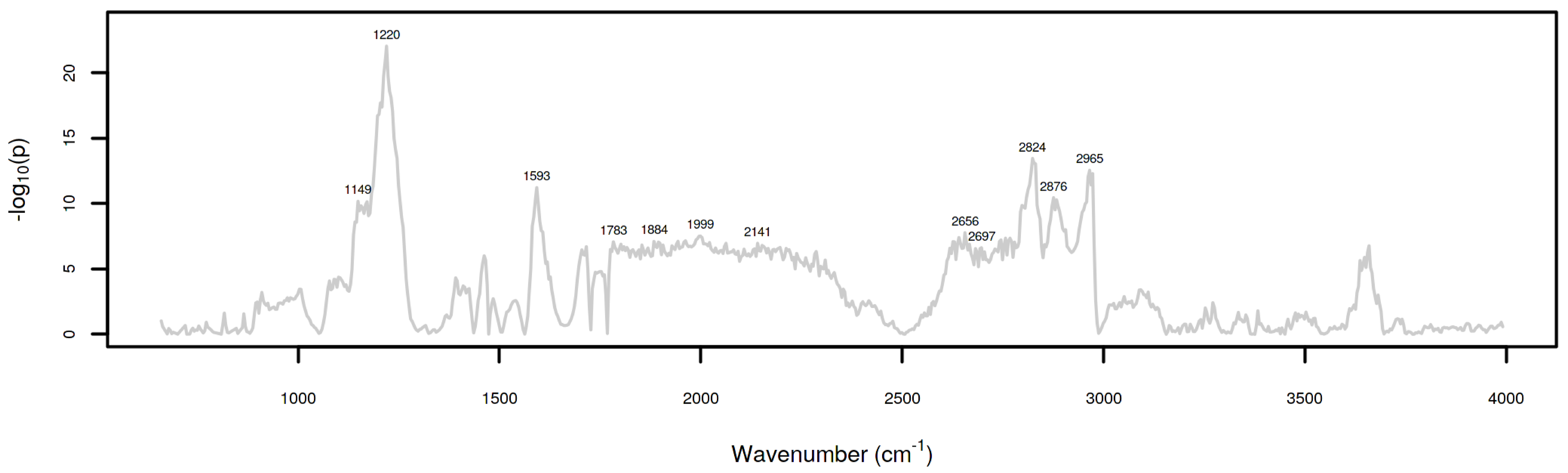

KCNJ2 (Chr19:61134515)

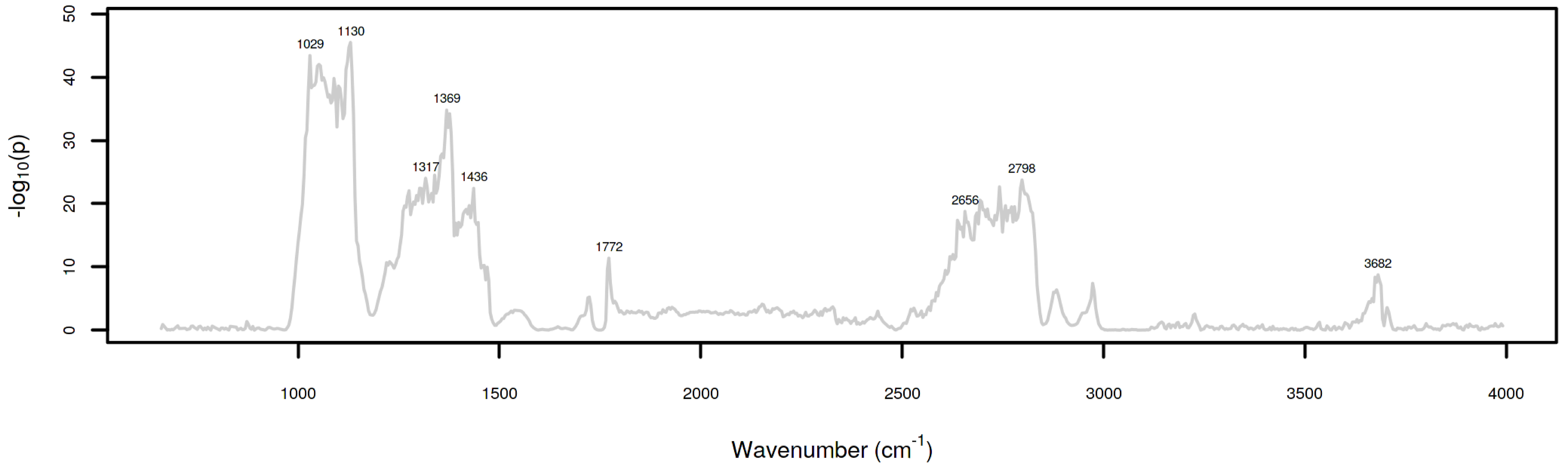

ANKH (Chr20:58454531)

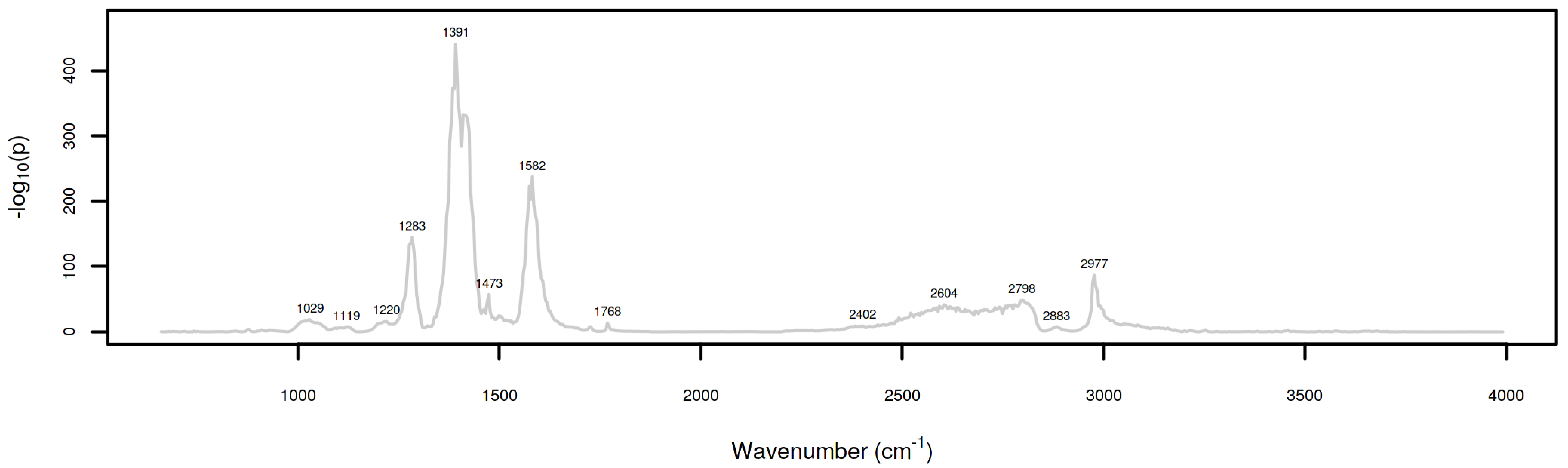

LTF (Chr22:53519865)

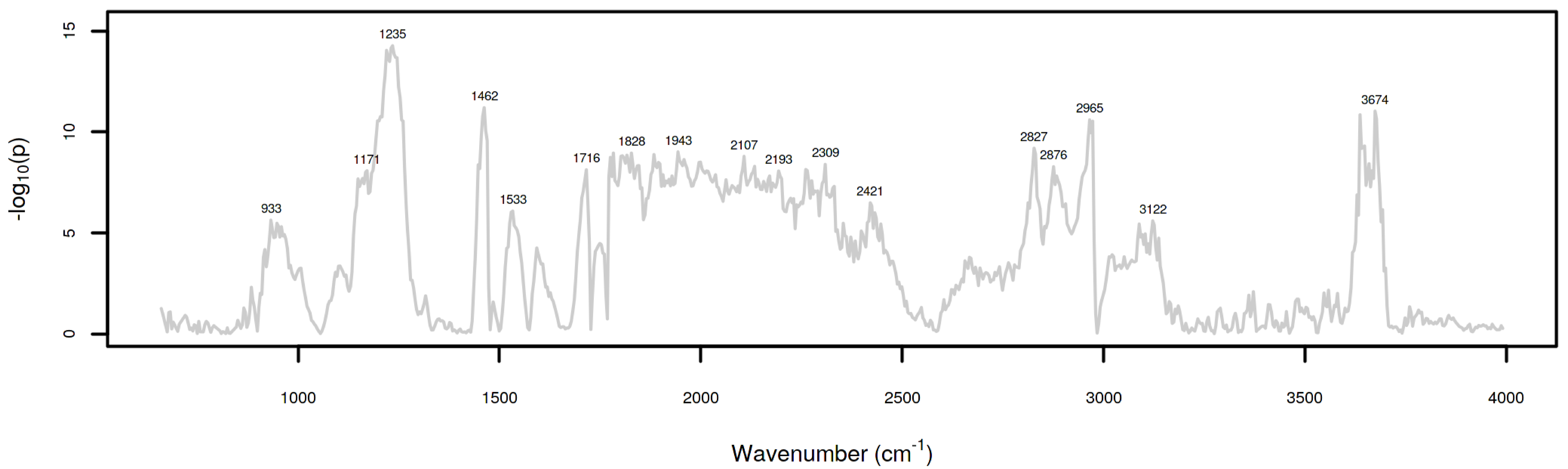

LMAN1 (Chr24:58817202)

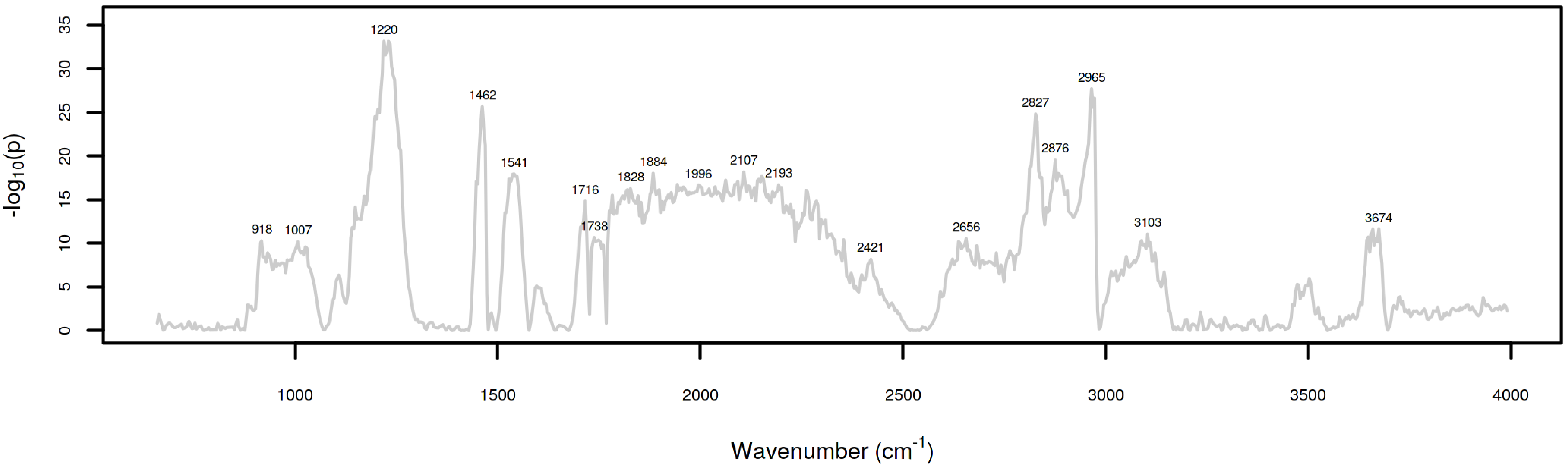

GPAT4 (Chr27:36211708)

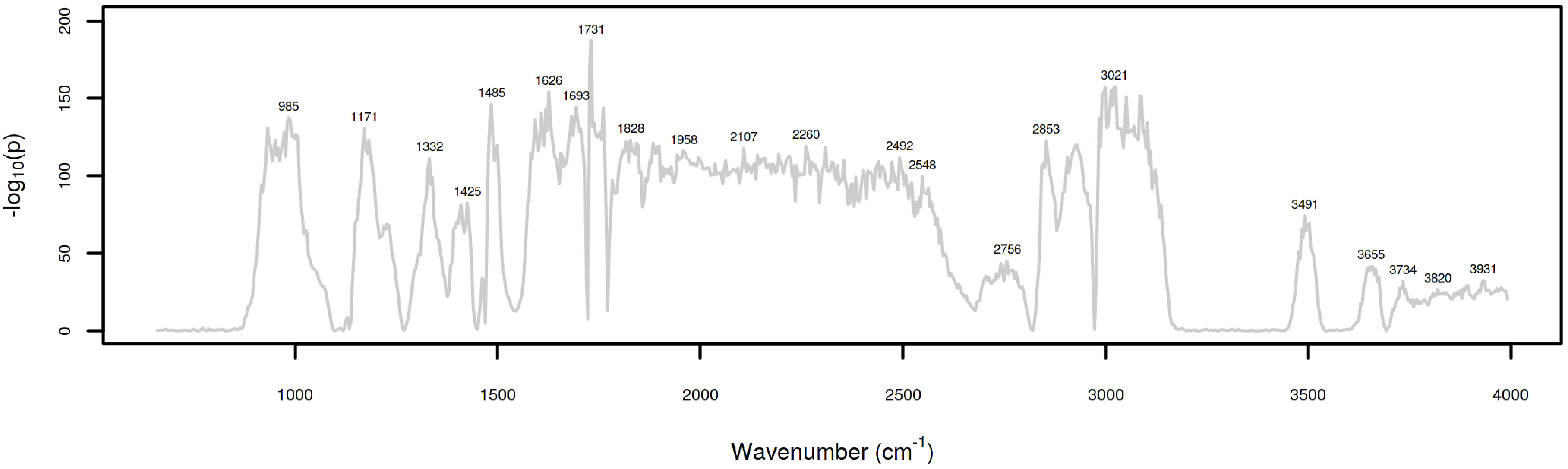

THRB (Chr27:41267242)

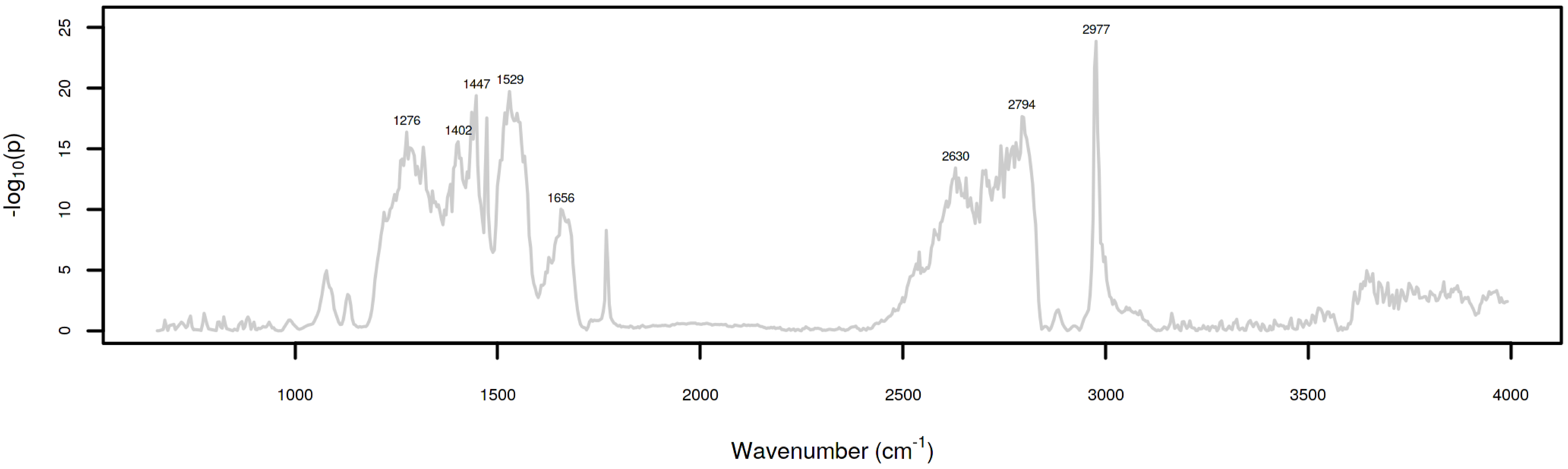

KCNK1 (Chr28:6559147)

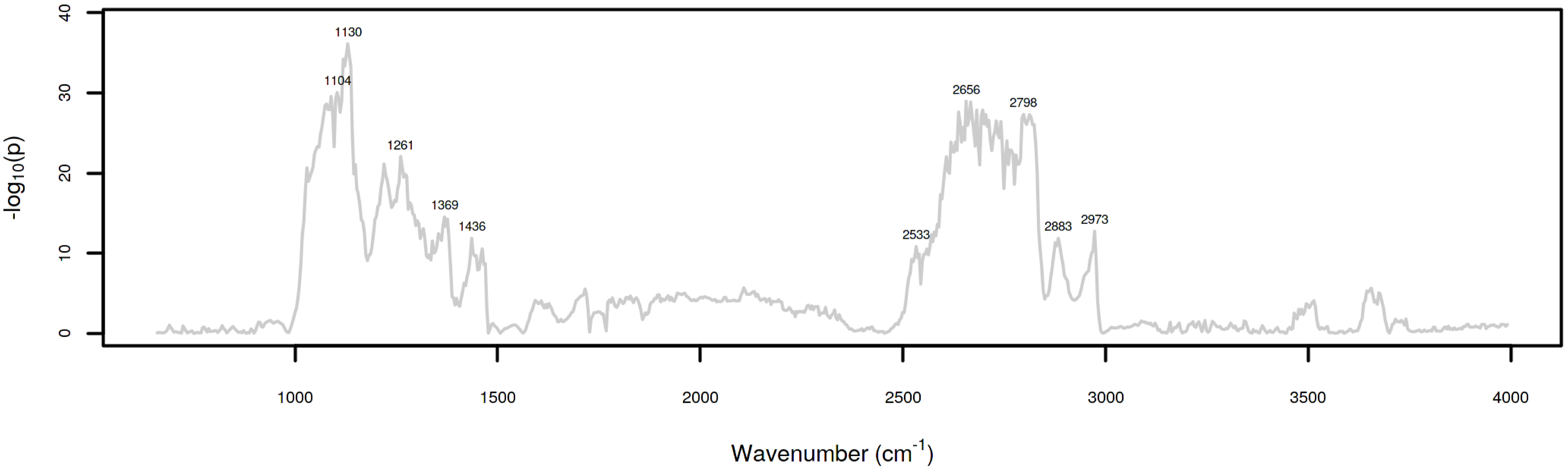

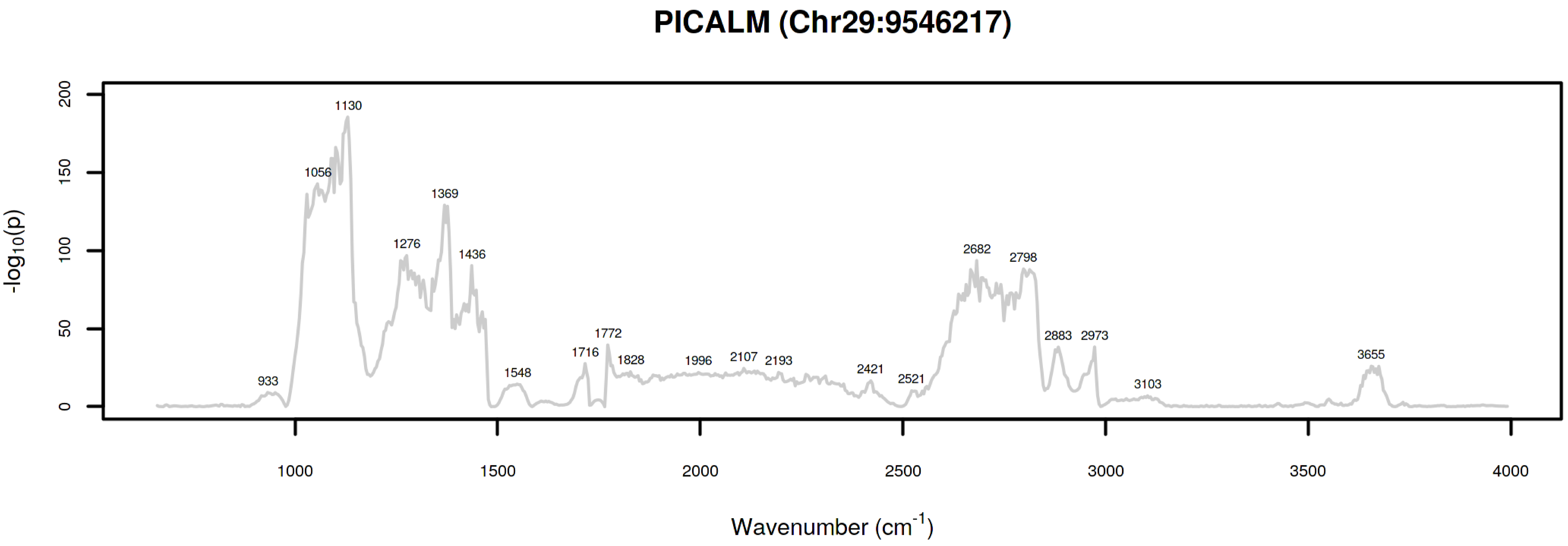
PICALM (Chr29:9546217)
-log10(p)
0
50
100
150
200
1000
1500
2000
2500
3000
3500
4000
Wavenumber (cm-1)
933
1056
1130
1276
1369
1436
1548
1716
1772
1828
1996
2107
2193
2421
2521
2682
2798
2883
2973
3103
3655

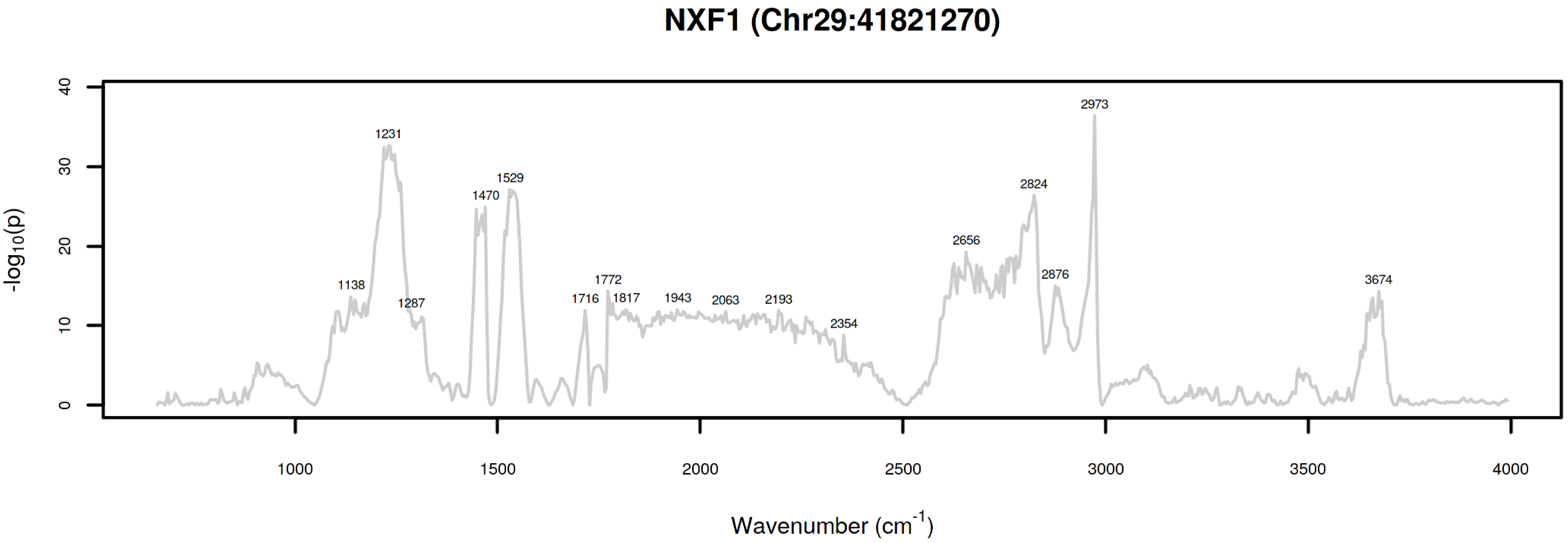
NXF1 (Chr29:41821270)
-log10(p)
0
10
20
30
40
1000
1500
2000
2500
3000
3500
4000
Wavenumber (cm-1)
1138
1231
1287
1470
1529
1716
1772
1817
1943
2063
2193
2354
2656
2824
2876
2973
3674

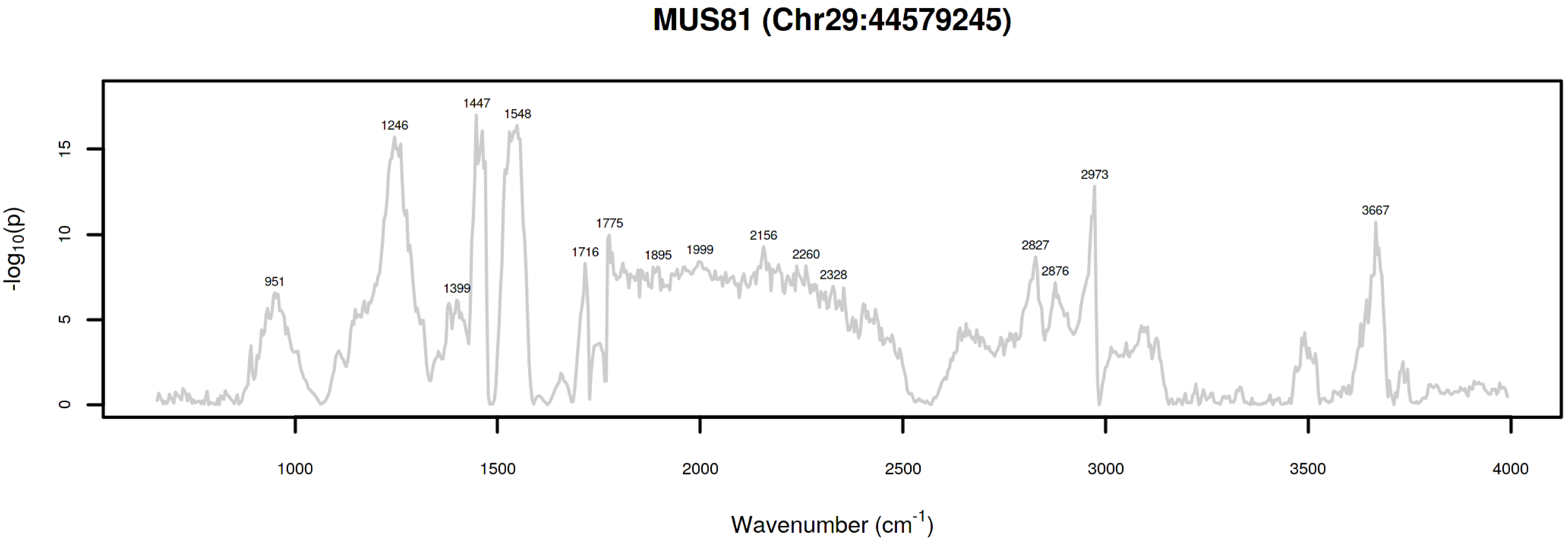
MUS81 (Chr29:44579245)
-log10(p)
0
5
10
15
1000
1500
2000
2500
3000
3500
4000
Wavenumber (cm-1)
951
1246
1399
1447
1548
1716
1775
1895
1999
2156
2260
2328
2827
2876
2973
3667
